# Perceptual Novelty Drives Early Exploration in a Bottom‐Up Manner

**DOI:** 10.1111/desc.70002

**Published:** 2025-03-03

**Authors:** Mengcun Gao, Vladimir M. Sloutsky

**Affiliations:** ^1^ Department of Psychology The Ohio State University Columbus Ohio USA

**Keywords:** cognitive development, computational modeling, decision‐making, exploration‐exploitation dilemma, stimulus‐driven attention

## Abstract

Children are more likely than adults to explore new options, but is this due to a top‐down epistemic‐uncertainty‐driven process or a bottom‐up novelty‐driven process? Given immature cognitive control, children may choose a new option because they are more susceptible to the automatic attraction of perceptual novelty and have difficulty disengaging from it. This hypothesis is difficult to test because perceptual novelty is intertwined with epistemic uncertainty. To address this problem, we designed a new n‐armed bandit task to fully decouple novelty and epistemic uncertainty. By having adults and 4‐ to 6‐year‐olds perform the task, we found that perceptual novelty predominated 4‐year‐olds’ (but not adults’ or older children's) decisions even when it had no epistemic uncertainty and had the lowest reward value. Additionally, 4‐year‐olds showed such a novelty preference only when the option's novelty was directly observable, but not when it could only be anticipated, providing new evidence that perceptual novelty alone can drive elevated exploration in early development in a bottom‐up manner.

## 1 Introduction

When choosing between the tried and true and the novel, young children (particularly preschoolers) are more likely than adults to explore the novel option. For example, when choosing a bet in a multi‐arm bandit, adults tend to exploit the most rewarding option, whereas young children are more likely to explore novel options (Blanco and Sloutsky 2020, 2021; Cockburn et al. 2022; Dubois et al. 2022; Nussenbaum et al. 2023; E. Schulz et al. 2018). Prior research has suggested that young children's heightened exploration arises from greater curiosity—an intrinsic drive to gather information.

**Summary**

- Due to immature attentional systems, children's novelty‐driven decisions may result from their susceptibility to automatic attentional capture by perceptual novelty.
- One barrier to examining this hypothesis is that perceptual novelty is intertwined with epistemic uncertainty, making it challenging to investigate its independent effects.
- To examine whether perceptual novelty alone is sufficient to drive early exploration, we adopted a new paradigm to fully decouple perceptual novelty and epistemic uncertainty.
- The results demonstrated that perceptual novelty alone could drive elevated exploration in early development, presumably in a bottom‐up manner.

Specifically, curiosity may drive people to glean information that is new, uncertain, and potentially valuable to them (Berlyne 1954; Blanco and Sloutsky 2021; Liquin, Callaway, and Lombrozo 2021; Molinaro et al. 2023; Poli et al. 2020, 2022; Ruggeri et al. 2024; Wilson et al. 2021). Given that childhood is a special period characterized by resource abundance (due to parental care), limited knowledge, and an expanded temporal horizon for learning, it is both natural and beneficial for young children to prioritize information‐seeking (exploration) over utilizing their current knowledge to maximize rewards (exploitation).

However, findings on young children's information‐driven exploration are mixed. Some research found that young children displayed higher levels of *systematic* exploration (explore to gain information and reduce uncertainty) compared to adults (Blanco and Sloutsky 2021; Giron et al. 2023; Gopnik 2020; Poli et al. 2024; Ruggeri et al. 2024; Schulz et al. 2018). However, other research found that young children continued to sample information when there was no information to gain (Ruggeri et al. 2016; Wan and Sloutsky 2024), indicating that information‐seeking is not the only driver of children's choices.

Moreover, even when found, young children's systematic exploration is unlikely to be *goal‐directed* and *information‐driven*. This is because if young children's exploration is driven by their quest for information, they should be able to estimate their own state of knowledge and the potential information gain, the estimation requiring the levels of metacognitive abilities that they may not be capable of (Coughlin et al. 2015; Hembacher and Ghetti 2014; Kim, Berry, and Carlson 2025; Lockl and Schneider 2004; Niebaum et al. 2021; O'Leary and Sloutsky 2017, 2019; Schulz et al. 2023). More importantly, the fact that making one option perceptually salient could disrupt young children's exploration (Blanco and Sloutsky 2020) suggests that such exploration may not be goal‐directed. These findings suggest that a potentially stimulus‐driven attention mechanism, rather than the intrinsic value of information, may underlie young children's exploration.

Following up on this idea, we propose that young children's elevated exploration can result from the interaction between a bottom‐up novelty effect and immature cognitive control. Specifically, early in development, perceptually novel stimuli can automatically capture attention, with cognitive control not being mature enough to inhibit or disengage this attentional capture. The primary goal of the paper is to test this hypothesis. However, although this hypothesis presents a compelling framework for understanding heightened exploration in early development, as we explain below, testing it is quite challenging. In what follows, we will first discuss how perceptual novelty can drive children's exploration in a bottom‐up manner. We then identify critical challenges in testing this hypothesis and provide an overview of how our approach meets this challenge.

### 1.1 Perceptual Novelty May Drive Young Children's Exploration in a Bottom‐Up Manner

Human attention can be controlled in a top‐down way that is initiated voluntarily and based on task demands and agents’ goals, or in a bottom‐up way that is automatically triggered by stimulus properties (Theeuwes 2005). It is broadly recognized that stimuli that are distinct from surrounding ones (e.g., a red circle among green circles) and/or violate expectations can automatically capture people's attention in a bottom‐up manner (Fecteau and Munoz 2006; Horstmann and Herwig 2016; Theeuwes 2005).

Perceptual novelty, with its inherent contrast to familiarity and expectations, has been shown to automatically capture attention (Hillstrom and Yantis 1994; Horstmann 2002; Horstmann and Herwig 2016; Neo and Chua 2006; Ranganath and Rainer 2003; Retell et al. 2016). When involuntary attention to task‐irrelevant stimuli is counterproductive (e.g., leading to poor decision‐making), adults with mature cognitive control, can inhibit automatic reactions to such stimuli and redirect attention to task‐relevant stimuli (Gaspelin et al. 2015). However, young children, with immature cognitive control, are more susceptible to the attraction of novel stimuli (Turoman et al. 2021) and have more difficulty suppressing spontaneous responses to such stimuli and disengaging attention from them (Schul et al. 2003; Wetzel et al. 2009).

Previous studies have provided initial evidence that the exploratory behaviors of people with immature or impaired cognitive control can be driven by stimulus perceptual novelty. For example, introducing a highly salient novel option disrupted young children's systematic exploration (Blanco and Sloutsky 2020). In addition, populations with impulse control problems, such as those with ADHD or Parkinson's disease exhibiting impulsive compulsive behaviors, were more prone to choose novel options regardless of rewards (Djamshidian et al. 2011; Sethi et al. 2018). These studies highlighted an association between attention and novelty‐driven exploration, implying that novelty can impact decision‐making in a bottom‐up way when top‐down cognitive control is less effective.

### 1.2 A Challenge in Examining the Effects of Perceptual Novelty

One significant challenge in examining the effects of perceptual novelty is that perceptual novelty is intertwined with epistemic uncertainty. Epistemic uncertainty, in contrast with objective uncertainty that refers to the variability or unpredictability of the outcomes, results from people's lack of knowledge about an outcome. The distinction is important because objective uncertainty cannot be fully resolved by gathering information (e.g., repeatedly rolling a die won't improve the prediction of the next roll), whereas epistemic uncertainty can (e.g., reading a fiction novel to discover its ending).

Previous research has tried to tease apart novelty and epistemic uncertainty and provided initial evidence that novelty has dissociable impacts from uncertainty (Blanco and Sloutsky 2021; Brown et al. 2022; Cockburn et al. 2022; Costa et al. 2014; Dubois et al. 2022; Houillon et al. 2013; Nussenbaum et al. 2023; Poli et al. 2022; Wittmann et al. 2008). However, despite substantial progress, novelty and epistemic uncertainty have not been fully decoupled. For example, many prior studies examined the effects of novelty by comparing participants’ choice decisions between novel and familiar options. However, when a novel option is introduced, it by definition carries epistemic uncertainty because its attributes are unknown (e.g., Cockburn et al. 2022; Dubois et al. 2022; Wittmann et al. 2008). Alternatively, if novelty is construed as the infrequency of encounters rather than the initial encounter, such a construal might underestimate the impact of novelty (Blanco and Sloutsky 2020; Nussenbaum et al. 2023; Poli et al. 2022).

As a result, it remains unclear whether (a) perceptual novelty, independent of epistemic uncertainty, can drive exploration in early development by automatically capturing attention or (b) children explore novel options because of their epistemic uncertainty. This distinction is consequential because it points to drastically different mechanisms underlying exploration: a bottom‐up novelty‐driven process versus a top‐down uncertainty‐driven process. The goal of the present research is to address this issue.

To achieve this goal, we introduce a new paradigm that disentangles novelty and epistemic uncertainty, thus allowing us to examine whether perceptual novelty without epistemic uncertainty is sufficient to drive exploration in early development.

### 1.3 Current Study

The current study consisted of two experiments in which participants (4‐to‐6‐year‐old children and adults) chose from multiple options, each yielding different rewards and having different latent properties (i.e., uncertainty, novelty). Computational models were developed to evaluate the factors driving participants’ decisions.

Experiment 1 was conducted to examine (1) whether the paradigm used in our study replicates the exploratory patterns previously observed in young children, and (2) whether young children prioritize rewards when novelty and uncertainty are absent. This is important to rule out the possibility that novelty‐driven decisions found in our study were due to children's misunderstanding of the task and/or their insensitivity to task‐defined rewards.

Building on the initial evidence of children's novelty‐driven decisions in Experiment 1, we conducted Experiment 2 to fully decouple epistemic uncertainty and perceptual novelty as potential drivers of young children's exploratory behaviors. Experiment 2 provided novel evidence for the influence of perceptual novelty independent of any sources of uncertainty.

Moreover, to further examine potential mechanisms of novelty‐driven decisions, we introduced a distinction between observed and anticipated perceptual novelty: whereas the former is experienced directly, the latter is not. For example, perceptual novelty is experienced directly when people can observe it, such as when they see a new toy. In contrast, novelty is *signaled* (i.e., predicted or anticipated), when a distinct symbol (e.g., a red triangle) indicates that the toy is novel, without the toy being visible. If novelty influences exploration‐related decisions in a goal‐directed manner, then both directly experienced and anticipated novelty of an option should motivate participants to explore it. By contrast, if novelty drives exploration in a bottom‐up (i.e., stimulus‐driven) manner, then directly experienced novelty should be a substantially stronger driver of exploration. To foreshadow the results, we found that perceptual novelty drives children's choices in a bottom‐up manner.

## 2 Experiment 1

### 2.1 Participants

Experiment 1 included 30, 4‐year‐olds (*M_age_* = 4.48 years, *SD_age_* = 0.29, 13 girls), 32, 5‐ to 6‐year‐olds (*M_age_* = 5.60 years, *SD_age_* = 0.54, 17 girls), and 32 adults (*M_age_* = 25.49 years, *SD_age_* = 11.37, 26 females). Children of these ages were selected because 4‐year‐olds have immature cognitive control, with 5‐ to 6‐year‐olds exhibiting substantial developmental improvements (Deng and Sloutsky 2015, 2016; Ikeda et al. 2014; Plebanek and Sloutsky 2019; Van ’T Wout et al. 2019).

The sample size was determined based on a prior study with a similar experimental design (Blanco and Sloutsky 2020), which found age differences between 4‐ and 5‐year‐olds and adults in selecting a perceptually different and uncertain option with the lowest value and reported an effect size of 𝑑 = 1.24. The power (1−𝛽) to detect such an effect with *N* = 30 for each group at 0.05 significance level (𝛼) is 0.997. Therefore, we recruited *N* = 30 participants for each age group.

For both experiments, adult participants were undergraduate students at Ohio State University who participated in research for course credit. Child participants were recruited from the Columbus metropolitan area. The study was approved by the Ohio State University Institutional Review Board, and informed consent was obtained from all adult participants and from the parents of child participants before their participation.

### 2.2 Stimuli and Procedure

In Experiment 1, stimuli included 63 distinct pixel art images of cartoon animals from Pinterest. These pictures were presented on different colored squares on each trial, and each unique pair of an animal image and a colored square formed an “option” in the experiment.

Experiment 1 contained a training phase followed by a testing phase (30 trials for each phase). Both phases employed a simplified n‐armed bandit task in which participants collected rewards (framed as gold coins) from one of multiple options. In the training phase, participants chose among three options, and in testing, a fourth option was introduced. Each option was placed in the same location throughout the experiment.

The reward value associated with each option on each trial was initially drawn from its predetermined value distribution and rounded to the nearest whole number. Then, values exceeding a predetermined upper bound or falling below a predetermined lower bound were adjusted to the boundaries. The simulated reward distributions for all the options are shown in Figure S1. Based on the value distribution, the three training options were referred to as the high‐reward option (*µ* = 20, *σ* = 1, lower bound = 18, upper bound = 22), high‐uncertainty option (*µ* = 11, *σ* = 10, lower bound = 0, upper bound = 22), and low‐all option (*µ* = 2, σ = 1, lower bound = 0, upper bound = 4). Importantly, during training, the reward values carried no epistemic uncertainty, as the reward value for each option was shown on every trial, prior to the participant making their choice. The three training options were designated as “familiar” (or familiarized), so that the associated animal images and colored squares remained the same throughout the experiment.

**FIGURE 1:**
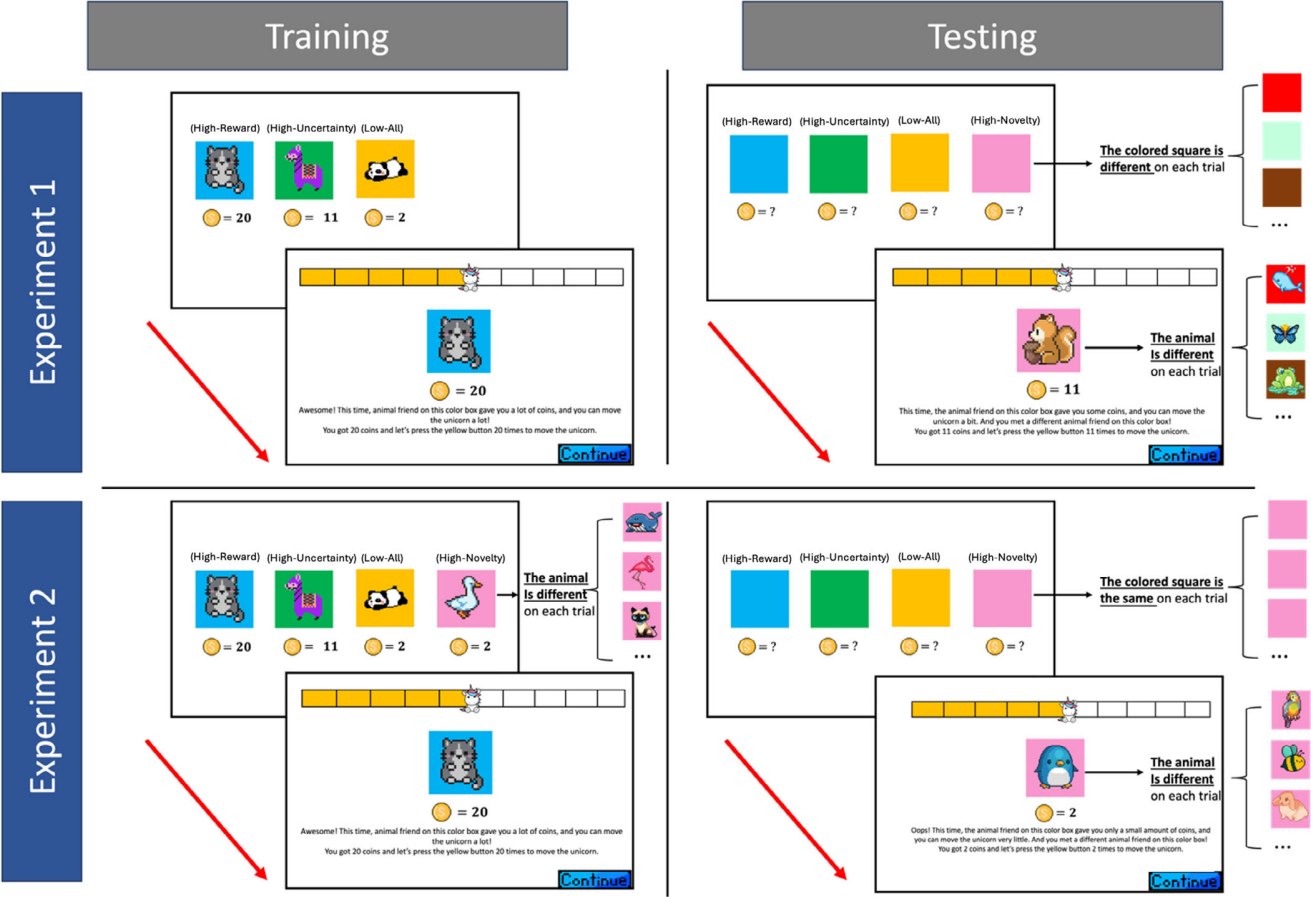
Example trials and feedback for training (left) and testing (right) phases in the child version of Experiment 1 (top) and Experiment 2 (bottom). During training (the left column), in both experiments, the reward information (i.e., the number of coins shown below animal figures and colored squares) was directly shown to participants before they made a choice. By contrast, the reward information was concealed during testing (the right column) and was shown for the chosen option after the participants made their choices. In each quadrant, the sequence represents the choice followed by the feedback screen. In the figure, the blue‐chinchilla, green‐alpaca, yellow‐panda, and pink (or other colors)‐different‐animal combinations denote the high‐reward, high‐uncertainty, low‐all, and high‐novelty options (the labels above options were added to help with the understanding of the task for readers, but were not shown to participants during the experiments). In the real experiments, however, except that the high‐novelty option was always presented on the rightmost in Experiment 1, the colors, animals, and locations of all other options were randomized for each participant.

In testing, in addition to the three options above, a high‐novelty option (*µ* = 11, *σ* = 10, lower bound = 0, upper bound = 22) was introduced. The presented animal and colored square for the high‐novelty option were different on each trial (as shown in Figure 1, top‐right), with a changing color providing directly experienced perceptual novelty. In addition, the amount of information provided differed between training and testing. As mentioned above, during training, participants saw the number of rewards, colored squares, and animal images for all options on each trial. However, in testing, only the colored squares were displayed, with reward values and animal pictures hidden from participants. Because the colored square for the high‐novelty option was different on each testing trial but remained unchanged for the other three options, only the high‐novelty option had elevated perceptual novelty.

In both phases, participants were asked to maximize rewards (the total number of coins collected). They received feedback after making choices. During the feedback, the number of rewards, the colored square, and the animal image associated with the chosen option were all displayed on the screen.

The procedure was similar for adults and children, with several differences introduced to help children better understand the instructions and be more engaged with the task. Detailed procedural differences between adults and children are described in Supplemental Materials.

After the completion of the task, two follow‐up memory questions were administered to children, asking them to point to the option that always gave them a lot of coins (high‐reward question), and the option that showed them a different animal friend on every trial (high‐novelty question).

### 2.3 Computational Modeling

The primary goal of computational modeling was to examine the differential impacts of option attributes on children's and adults’ choice decisions. We assumed that participants’ choices in the current task could be influenced by (expected) reward values, reward variability, perceptual novelty, and choice lag. Choice lag tracked the time elapsed since the option was last selected, capturing participants’ lag‐based switching exploration pattern. Lag‐based switching exploration patterns could reflect participants’ anti‐preservation bias or indicate epistemic‐uncertainty‐driven exploration, with longer lag times resulting in greater epistemic uncertainty of an option on a given trial (Blanco et al. 2023; Blanco and Sloutsky 2020, 2021). However, given that Experiment 1 was unable to differentiate these two interpretations and that option rewards were directly shown to participants only in training but not testing (thus probably requiring different interpretations), we chose to not over‐interpret this parameter. Instead, we treated it as an indicator of one type of exploration pattern.

By incorporating the impacts of all choice attributes, each option *i*’s relative utility on trial *t* (*V*_*i*,*t*_), is calculated by Equations (1a) and (1b) in the training phase for Experiments 1 and 2, respectively:

(1a) $$V_{i,t}\;\;=w_{v\_train}\;R_{i,t}+w_{l\_train}L_{i,t}+w_{u\_train}\sigma_{i,t}\;$$

(1b) $$\begin{array}{c} V_{i,t}\;\;=w_{v\_train}\;R_{i,t}+w_{l\_train}L_{i,t}+w_{u\_train}\sigma_{i,t}+w_{n\_train}\delta_{\left[i=novel\right]} \end{array}$$

and calculated by Equation (2) in the testing phase for both experiments:

(2) $$V_{i,t}\;\;=w_{v\_test}\;\mu_{i,t}^{\prime }+w_{u\_test}\sigma_{i,t}+w_{l\_test}L_{i,t}+w_{n\_test}\delta_{\left[i=novel\right]}$$

In training, *V*_*i*,*t*_ is determined by each option's observed reward value (*R*_*i*,*t*_), choice lag (*L*_*i*,*t*_), and expected objective uncertainty (indexed by standard deviation, σ_*i*,*t*_) in Experiment 1, and reward value, choice lag, expected objective uncertainty, and perceptual novelty in Experiment 2 (δ_[*i*  =  *novel*]_ = 1, for high‐novelty option, and δ_[*i*  =  *novel*]_ = 0, for other options). In testing, *V*_*i*,*t*_ is determined by each option's expected reward value ($\mu_{i,t}^{\prime }$), choice lag, expected objective uncertainty, and perceptual novelty.

Specifically, $w_{v\_train},w_{l\_train},\;w_{u\_train},w_{n\_train}$ are free parameters that represent the relative weights of reward value, choice lag, expected objective uncertainty, and perceptual novelty in the training phase. Similarly, $w_{v\_test},w_{l\_test},\;w_{u\_test},w_{n\_test}$ are relative weights of expected reward values, choice lag, expected objective uncertainty, and novelty in the testing phase. All the weights take values between 0 and 1, and weights contributing to the same relative utility calculation sum to 1. Min‐max normalization was applied to *R*_*i*,*t*_, µ_*i*,*t*_, and σ_*i*,*t*_ at the trial level, and to *L*_*i*,*t*_ at the phase level.

The relative utilities are then mapped onto choice probabilities by the following softmax function:

(3) $$P\;\left(i,t\right)=\frac{e^{\gamma V_{i,t}}}{\sum_{x}e^{\gamma V_{i,t}}}\;$$

where *P*(*i*, *t*) is the probability of choosing option *i* on trial *t*, and γ > 0 is the inverse temperature parameter that reflects the randomness of individuals’ exploration, with lower γ indicating more random exploration.

Moreover, given that the reward distributions were unknown to the participants, we assumed that participants updated their beliefs about the mean and variance of each reward distribution as they accumulated evidence either through direct observation (in training) or through their choices (in testing), with the mean and variance of each option not reset between training and testing. Accordingly, the model adopted a Bayesian updating mechanism for learning mean reward values and their variance (Dearden et al. 1998). Technical details of the Bayesian updating mechanism were provided in the Supplemental Materials.

### 2.4 Behavioral Results

#### 2.4.1 Memory Test

Preliminary analysis evaluated the accuracy of children's recognition of the high‐reward and high‐novelty options. Binomial tests were conducted to determine if children's correct responses to the high‐reward and high‐novelty memory questions were significantly above the chance level of 0.25. Detailed binomial test results are reported in Table S2. Importantly, we reported above‐chance accurate memory for high‐reward and high‐novelty options in both child age groups (*p*s < 0.003), indicating that children as a group, reliably identified the two options.

#### 2.4.2 Choice Decision Results

**FIGURE 2:**
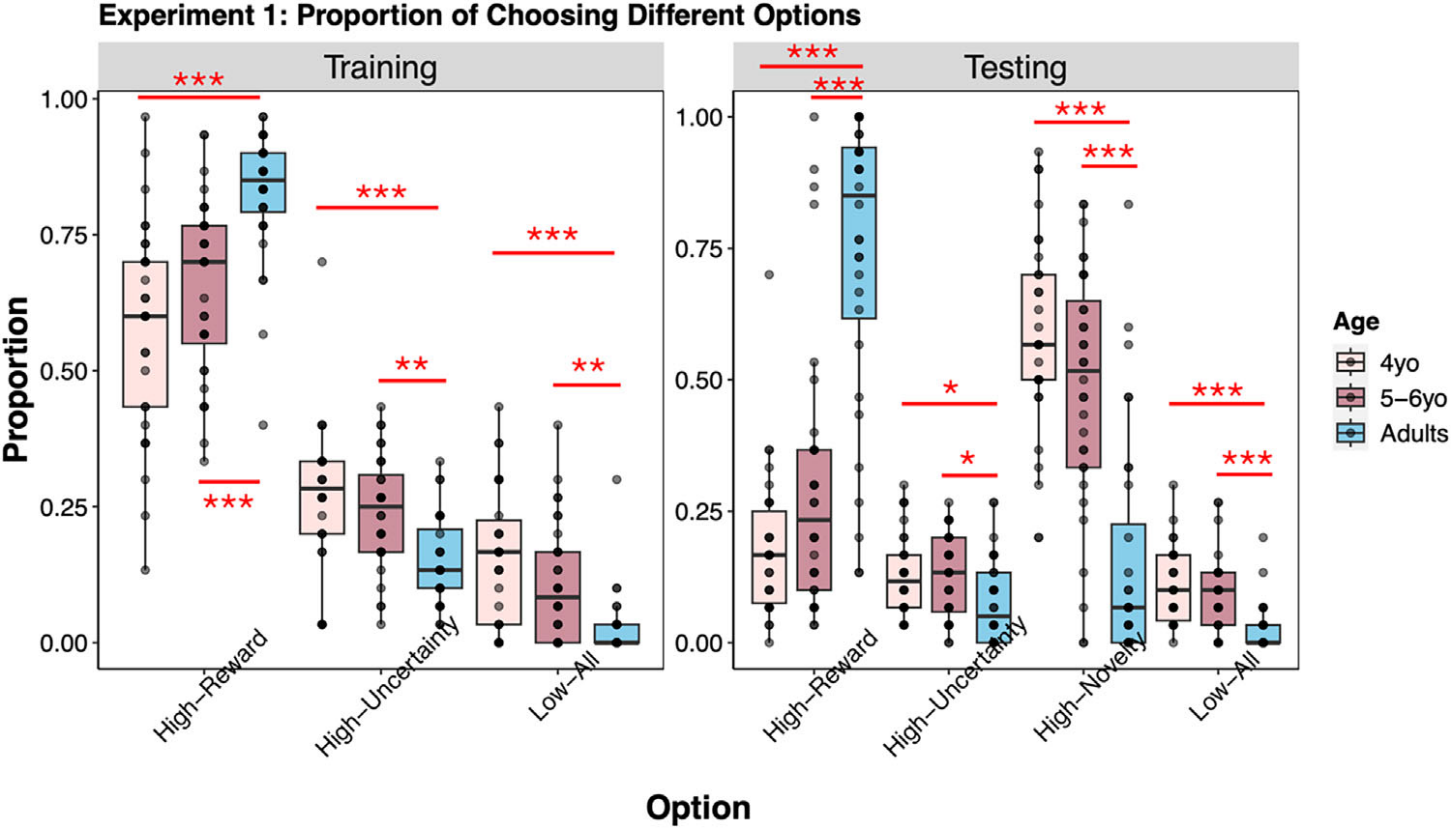
The proportion of choosing different options during the training (left) and testing (right) phase for 4‐year‐olds, 5‐ to 6‐year‐olds, and adults in Experiment 1. For this and all the boxplots in the paper, boxes represent the interquartile range (IQR) of data points, with lines within each box representing the median value. The whiskers extend from each box to the furthest data point that is within 1.5 times the IQR away from the box. Dots represent individual participants. Darker dots represent that more people share the same value. Statistical significance is annotated as *p* < 0.05 (^*^), *p* < 0.01 (^**^), and *p* < 0.001 (^***^).

Participants' choice proportions in training (left panel) and testing (right panel) in Experiment 1 are presented in Figure 2. As shown in the figure, all age groups exhibited a predominant preference for the high‐reward option in training, indicating that participants understood the task and maximized rewards in the absence of epistemic uncertainty or novelty. However, remarkable age‐related differences emerged during the testing phase. We focus on age differences in value‐based and novelty‐based decisions by analyzing the choice patterns of 4‐year‐olds, 5‐ to 6‐year‐olds, and adults (all other contrasts can be seen in Figure 2). Specifically, we conducted one‐way ANOVAs to compare proportions of choosing the high‐reward Option by age in both phases and the high‐novelty option in the testing phase^1^.

The results for training revealed a significant main effect of age for the high‐reward option, *F*(2, 91) = 18.68, *p* < 0.001, $\eta_{p}^{2}$  = 0.29. According to post hoc analysis with Tukey's HSD Test, adults (*M_adults_* = 0.83, *SD_adults_* = 0.12) were more likely to choose the high‐reward option compared to both age groups of children (*M*_4*yo*_ = 0.58, *SD*_4*yo*_ = 0.20; *M*_5 − 6*yo*_ = 0.65, *SD*_5 − 6*yo*_ = 0.16), *p*s < 0.001, with no significant difference between the two child groups, *p* = 0.193.

As for testing, the results revealed a significant main effect of age for both high‐reward, *F*(2, 91) = 47.29, *p* < 0.001, $\eta_{p}^{2}$ = 0.51, and high‐novelty: *F*(2, 91) = 33.27, *p* < 0.001, $\eta_{p}^{2}$ = 0.42. Adults were more likely to choose the high‐reward option (*M_adults_* = 0.74, *SD_adults_* = 0.28), and less likely to choose the high‐novelty option (*M_adults_* = 0.16, *SD_adults_* = 0.22), whereas children of both age groups tended to do the opposite (high‐reward: *M*_4*yo*_ = 0.18, *SD*_4*yo*_ = 0.14; *M*_5 − 6*yo*_ = 0.30, *SD*_5 − 6*yo*_ = 0.27; high‐novelty: *M*_4*yo*_ = 0.58, *SD*_4*yo*_ = 0.19; *M*_5 − 6*yo*_ = 0.48, *SD*_5 − 6*yo*_ = 0.23), *p*s < 0.001, with no differences between the two children's groups, *p*s > 0.1.

Together, the results revealed an age‐related decrease in novelty‐based decisions and an increase in value‐based decisions with development, suggesting differential impacts of reward value and perceptual novelty on children's and adults’ decisions. Consistently, a Bayesian multinomial logistic regression model fitted to each participant's choices in testing revealed that most adults predominately chose the high‐reward option, whereas most children clearly preferred the high‐novelty option (detailed results are shown in Supplemental Materials).

However, reward value and perceptual novelty were not the only factors influencing choice decisions. Instead, reward variability and choice lag might also affect participants’ choices. Therefore, to better understand age differences in factors driving their choice decisions, we conducted model fitting and analyses and reported the results in the following section.

### 2.5 Model Fitting and Results

#### 2.5.1 Full Model Fitting and Results

**FIGURE 3:**
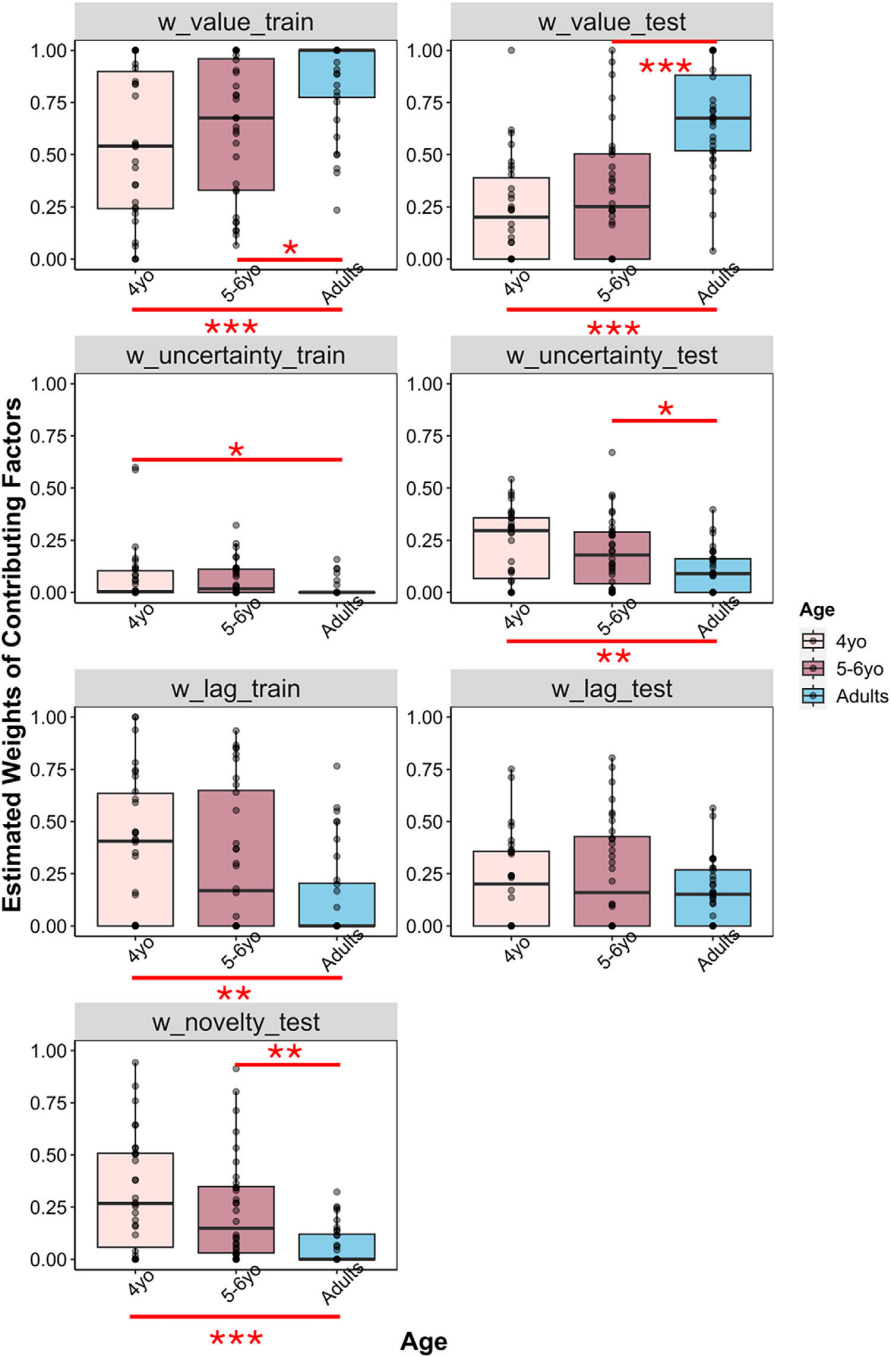
Distribution of best‐fitting values for all freely estimated weight parameters in the full model across age groups in Experiment 1.

The full model (outlined in the Computational Modeling section) was fit independently to each participant's observed data using the Nelder‐Mead method in R's optim function. The model was fit five times with 2000 iterations each time using different initial values to find the parameter values that minimized the negative log‐likelihood of the model. All parameter estimates are presented in Figure 3.

To investigate age differences in the weights of contributing factors, we conducted one‐way ANOVAs on best‐fitting parameter values of all contributing factors in each phase and ran pairwise comparisons between each two age groups. Given the primary focus of the paper, we reported only the pairwise comparison results for the weights of value and novelty in the main text, with complete results reported in the Supplemental Materials.

For training, we found significant age differences in the weight of value, *F*(2,91) = 8.131, *p* < 0.001, $\eta_{p}^{2}$ = 0.15, objective uncertainty, *F*(2,91) = 3.146, *p* = 0.048, $\eta_{p}^{2}$ = 0.06, and choice lag, *F*(2,91) = 5.175, *p* < 0.01, $\eta_{p}^{2}$ = 0.10. Particularly, the weight of value was higher for adults (*M_adults_* = 0.85, *SD_adults_* = 0.22) than for both 4‐year‐olds (*M*_4*yo*_ = 0.54, *SD*_4*yo*_ = 0.36) and 5‐ to 6‐year‐olds (*M*_5 − 6*yo*_ = 0.63, *SD*_5 − 6*yo*_ = 0.33), *p*s *<* 0.05, with no significant differences between the two child groups, *p =* 0.50.

For testing, we found a significant age difference in value, *F*(2,91) = 23.8, *p* < 0.001, $\eta_{p}^{2}$ = 0.34, novelty, *F*(2,91) = 10.92, *p* < 0.001, $\eta_{p}^{2}$ = 0.19, and objective uncertainty, *F*(2,91) = 6.514, *p* < 0.01, $\eta_{p}^{2}$ = 0.13, with no significant age effect on choice lag, *p* = 0.304. The weight of value was higher for adults (*M_adults_* = 0.67, *SD_adults_* = 0.25) than 5‐ to 6‐year‐olds (*M*_5 − 6*yo*_ = 0.32, *SD*_5 − 6*yo*_ = 0.30) and 4‐year‐olds (*M*_4*yo*_ = 0.23, *SD*_4*yo*_ = 0.25), *p*s *<* 0.001. More importantly, the weight of novelty was higher for 4‐year‐olds (*M*_4*yo*_ = 0.32, *SD*_4*yo*_ = 0.27) and 5‐ to 6‐year‐olds (*M*_5 − 6*yo*_ = 0.24, *SD*_5 − 6*yo*_ = 0.25) than adults (*M_adults_* = 0.06, *SD_adults_* = 0.10), *p*s *<* 0.01. The two child groups did not differ significantly from each other in both cases, *p*s > 0.34.

Additionally, we found age differences in the randomness of exploration (log‐transformed λ), with children exploring less deterministically than adults (we report the full analysis in Supplementary Materials). However, given that the majority of participants in all age groups were very deterministic, we chose not to over‐interpret these age differences.

In sum, significant age differences were found for all but the choice lag factor at test, indicating that children and adults made decisions based on different latent properties of options. Unlike adults who were primarily reward‐driven, children's decisions seemed to be driven by multiple factors. Importantly, 4‐year‐olds had a higher weight of perceptual novelty than all the other factors, suggesting that perceptual novelty might be the major driver of their decisions. Moreover, large individual differences in the weights of factors suggest that even in participants of the same age group, decisions may be driven by different factors. To examine individual differences in predominant factors driving their decisions, similar to the previous research (Blanco and Sloutsky 2021), we fit “single‐process” models to each individual participant.

#### 2.5.2 Single‐Process Model Fitting and Results

Four single‐process models (i.e., value‐based, uncertainty‐based, lag‐based, and random) were fit to participants’ training data, and five single‐process models (i.e., value‐based, uncertainty‐based, lag‐based, random, and novelty‐based) were fit to testing data. This was done by setting the weight of the primary factor at 1 and the weights of all the other factors at 0 for each model. Bayesian information criterion (BIC) was used to determine each participant's best‐fit single‐process model for the training and testing phase separately. Models except the random model assumed that participants’ exploration was primarily driven by one factor, whereas the random model assumed random responses without any trial‐to‐trial dependency.

**FIGURE 4:**
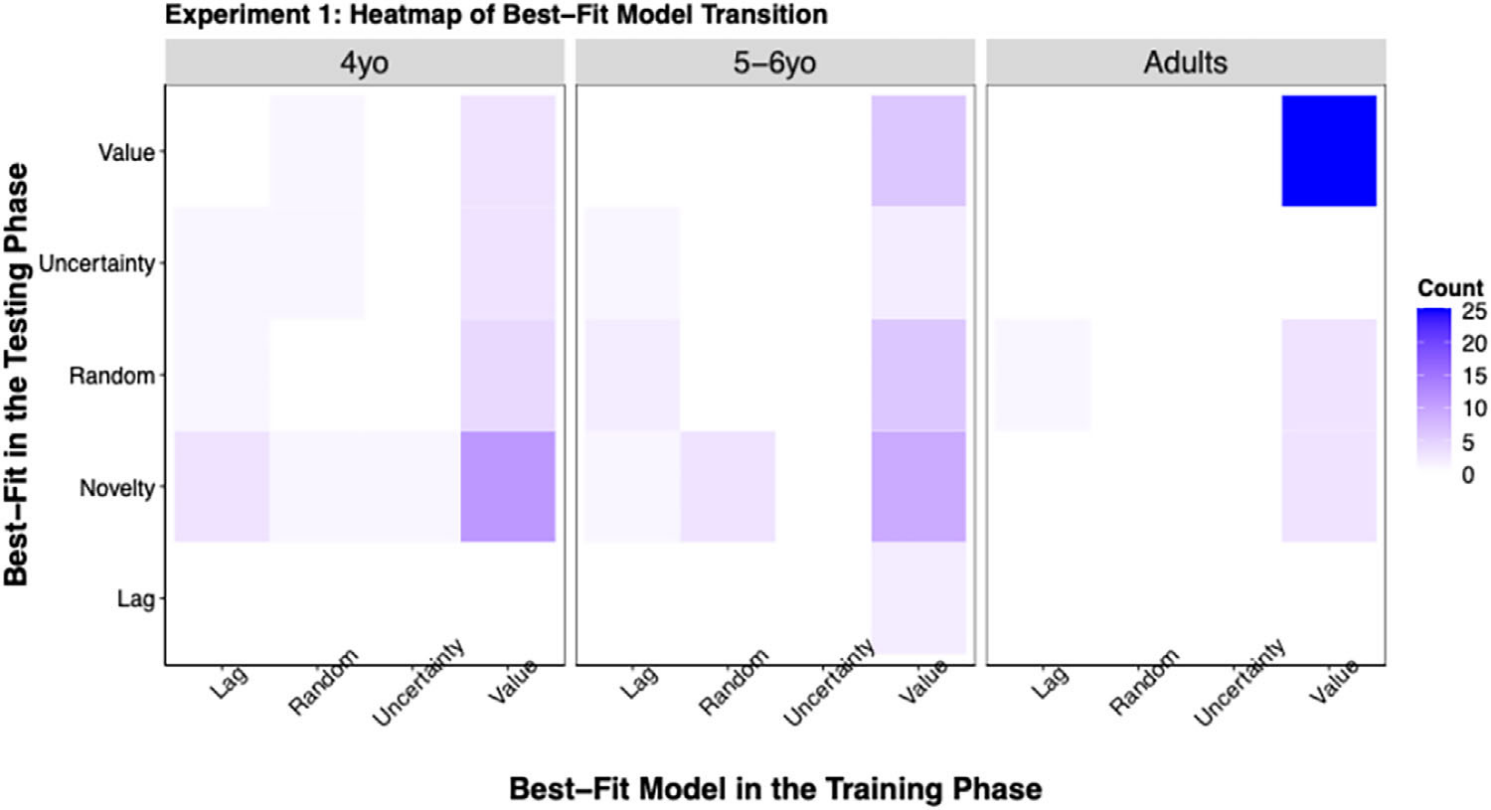
Heatmap of best‐fit model transition matrix in Experiment 1. The figure illustrates the transition from the best‐fit model in training to the best‐fit model in testing. Darker colors represent a higher frequency of transition.

The frequencies of the best‐fit model in the training and testing phases and the transition between them are shown in Figure 4. Given that most participants were best fit by the value‐ and the novelty‐based models, and in conjunction with the primary interest of the paper, our analyses focused on these two models.

During training, most participants (21 out of 30 4‐year‐olds, 25 out of 32 5‐ to 6‐year‐olds, and 31 out of 32 adults) were best fit by the value‐based model. Pairwise Fisher's Exact tests conducted on these proportions revealed significant differences between 4‐year‐olds and adults, *p* < 0.01. However, no significant differences were found between 4‐year‐olds and 5‐ to 6‐year‐olds, or between 5‐ and 6‐year‐olds and adults, *p*s > 0.16.

By contrast, at test, only a minority of children (4 out of 30 4‐year‐olds, 6 out of 32 5‐ to 6‐year‐olds), and the majority of adults (25 out of 32) were best fit by the value‐based model. Both child age groups were significantly different from adults, *p*s < 0.001, with no difference from each other, *p* > 0.99.

Importantly, across‐phase analyses revealed that more value‐based adults (25 out of 31) than 4‐year‐olds (3 out of 21) and 5‐ to 6‐year‐olds (6 out of 25) in training remained value‐based in testing, *p*s < 0.0001. For those who transitioned from the value‐based model to other models at test, more 4‐year‐olds (11 out of 21) transitioned to the novelty‐based model than adults (3 out of 31), *p* < 0.01. Additionally, more 5‐ to 6‐year‐olds (9 out of 25) than adults made this transition, although the difference was marginal, *p* = 0.07. Finally, in general, more 4‐year‐olds (16 out of 30) and 5‐ to 6‐year‐olds (13 out of 32) were best fit by the novelty‐based model than adults (3 out of 32) at test, *p*s < 0.03, with no significant differences between the child groups, *p* > 0.99.

### 2.6 Discussion

In Experiment 1, adults’ decisions were primarily driven by the expected value of the reward, whereas young children's decisions were largely driven by perceptual novelty. Particularly, even for children who were highly value‐based in training, the presence of a perceptually novel option affected their decision patterns by turning many of them into novelty‐based decision‐makers. Moreover, model‐fitting results suggested that perceptual novelty alone, independent of uncertainty, might serve as a primary drive for early exploration. However, such a conclusion requires further testing due to the reasons below.

First, given that children received additional feedback for the high‐novelty option (i.e., “And you met a different animal friend on this color box,” see Supplemental Materials), they might have learned that the high‐novelty option would always present them with a new animal. Thus, their decisions might be driven by their epistemic uncertainty of novel animals instead of the perceptual novelty of colored squares.

Moreover, although the reward distribution for the high‐novelty option remained unchanged, this information was unknown to participants. Particularly, since the high‐novelty option always appeared as a novel‐colored square, participants might assume a different reward distribution underlying each uniquely colored square. Consequently, children's novelty‐based decisions could be confounded by epistemic uncertainty about expected rewards.

To rule out these possibilities, we fully decoupled perceptual novelty and all sources of epistemic uncertainty in Experiment 2. In addition, the design of Experiment 2 provided an opportunity to examine whether perceptual novelty was strategic or affected children's decisions in a bottom‐up manner.

## 3 Experiment 2

### 3.1 Participants

Experiment 2 included 30 4‐year‐olds (*M_age_* = 4.51 years, *SD_age_* = 0.32, 13 girls), 30 5‐ to 6‐year‐olds (*M_age_* = 5.72 years, *SD_age_* = 0.51, 10 girls), and 34 adults (*M_age_* = 20.83 years, *SD_age_* = 3.27, 16 females).

### 3.2 Stimuli and Procedure

Experiment 2 included a training and a testing phase and three follow‐up memory questions that probed participants’ recognition of the high‐reward, high‐novelty, and high‐uncertainty options.

The training and testing procedures of Experiment 2 were similar to Experiment 1, except for two major differences. First, the high‐novelty option was introduced during training in Experiment 2 (as shown in Figure 1, bottom‐left), with the same reward distribution as the low‐all option (*µ* = 2, *σ* = 1, lower bound = 0, upper bound = 4).

Same as in Experiment 1, both the colored squares and animal images remained unchanged throughout the experiment for the high‐reward, high‐uncertainty, and low‐all options, whereas the high‐novelty option was accompanied by a different animal figure, but an unchanging colored square (the second difference from Experiment 1) on each trial in both phases. Importantly, the (new on every trial) animal was fully exposed in the training phase, thus allowing direct access to novelty, whereas novelty was signaled by location and color in testing, as the animal was hidden. To highlight, the unchanging colored square for the high‐novelty option was critically important as it made novelty in testing to be assumed or anticipated, rather than experienced directly.

By implementing these two changes, we were able to (1) decouple novelty and epistemic uncertainty and (2) examine whether perceptual novelty drives early decisions in a bottom‐up or top‐down manner. Given that all the information was presented during training (therefore no epistemic uncertainty), and that the high‐novelty option was associated with the lowest number of rewards, if children still favor this option, it would indicate that their choices are driven purely by *perceptual* novelty. Moreover, if children prioritize the high‐novelty option only during training, when perceptual novelty is directly observable, but not during testing, when novelty is only signaled, this would suggest that such novelty‐based decision is bottom‐up, stimulus‐driven, rather than goal‐driven.

### 3.3 Behavioral Results

#### 3.3.1 Memory Test

The detailed results of the binomial test on children's memories were reported in Table S3. The tests yielded *p*s < 0.05 for high‐reward and high‐novelty questions, but *p*s > 0.19 for high‐uncertainty questions for both child groups. The results indicated that children, as a group, correctly identified the high‐reward and the high‐novelty options, but not the high‐uncertainty option.

#### 3.3.2 Choice Decision Results

**FIGURE 5:**
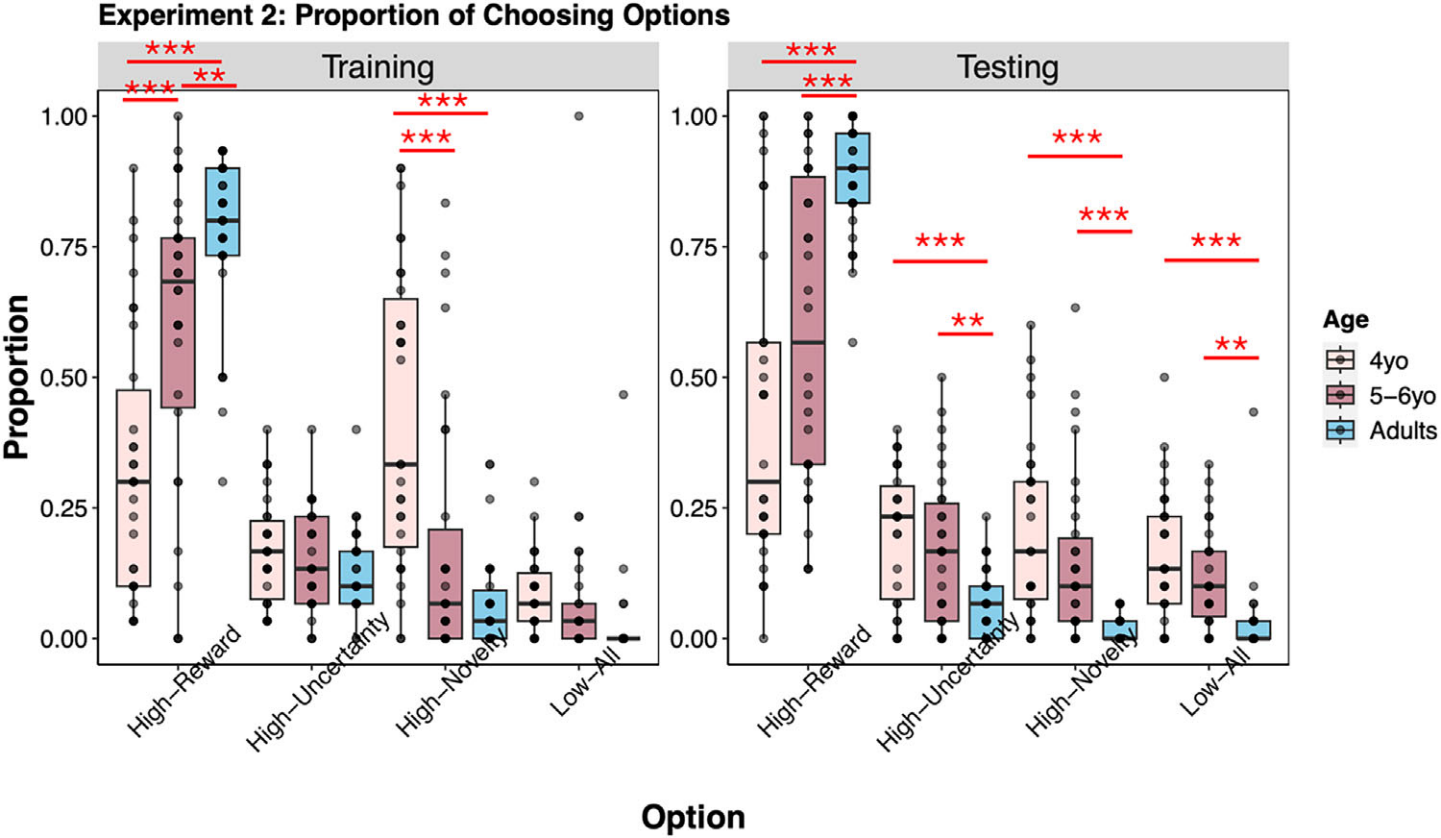
The proportion of choosing different options during the training (left) and testing (right) phase for 4‐year‐olds, 5‐ to 6‐year‐olds, and adults in Experiment 2.

To examine developmental differences in choice patterns, we conducted one‐way ANOVAs (with age as a factor) on the proportions of choosing the high‐reward option and the high‐novelty option in both training and testing phases^2^. Choice proportions for all four options are shown in Figure 5.

For training, we found a significant main effect of age for the high‐reward option, *F*(2, 91) = 27.65, *p* < 0.001, $\eta_{p}^{2}=$0.38. The post hoc analysis revealed that adults (*M_adults_* = 0.77, *SD_adults_* = 0.16) were more likely to choose the high‐reward option compared to both age groups of children (*M*_4*yo*_ = 0.33, *SD*_4*yo*_ = 0.26; *M*_5 − 6*yo*_ = 0.59, *SD*_5 − 6*yo*_ = 0.30), *p*s < 0.01, and 5‐ to 6‐year‐olds were more likely to choose the high‐reward option than 4‐year‐olds, *p* < 0.001. A significant age effect was also found for the high‐novelty option, *F*(2, 91) = 20.4, *p* < 0.001, $\eta_{p}^{2}$ = 0.31, with 4‐year‐olds (*M*_4*yo*_ = 0.42, *SD*_4*yo*_ = 0.28) being more likely to choose the high‐novelty option than both 5‐ to 6‐year‐olds (*M*_5 − 6*yo*_ = 0.18, *SD*_5 − 6*yo*_ = 0.25) and adults (*M_adults_* = 0.07, *SD_adults_* = 0.09), *p*s < 0.001. However, 5‐ to 6‐year‐olds did not significantly differ from adults, *p* = 0.135.

At the test, we also found a significant main effect of age for the high‐reward option, *F*(2, 91) = 27.46, *p* < 0.001, $\eta_{p}^{2}$ = 0.38, and for the high‐novelty option, *F*(2, 91) = 17.41, *p* < 0.001, $\eta_{p}^{2}$ = 0.28. According to the post hoc analysis, adults (*M_adults_* = 0.89, *SD_adults_* = 0.11) were more likely to choose the high‐reward option compared to both child groups (*M*_4*yo*_ = 0.43, *SD*_4*yo*_ = 0.31; *M*_5 − 6*yo*_ = 0.58, *SD*_5 − 6*yo*_ = 0.30), *p*s < 0.001. However, the difference between 5‐ and 6‐year‐olds and 4‐year‐olds was only marginally significant, *p* = 0.064. Meanwhile, adults (*M_adults_* = 0.02, *SD_adults_* = 0.02) were less likely to choose the high‐novelty option than 4‐year‐olds (*M*_4*yo*_ = 0.21, *SD*_4*yo*_ = 0.17) and 5‐ to 6‐year‐olds (*M*_5 − 6*yo*_ = 0.15, *SD*_5 − 6*yo*_ = 0.16), *p*s < 0.001, with no significant difference between the two child groups, *p* = 0.171.

Moreover, to directly examine the differential effects of the presence versus absence of visible perceptual novelty on children's and adults’ decisions, we conducted mixed ANOVAs on participants’ proportions of choosing the high‐novelty option, with age as a between‐subject variable, and phase as a within‐subject variable. The results revealed significant main effects of age, *F*(2,91) = 25.88, *p* < 0.001, $\eta_{p}^{2}$ = 0.36, phase, *F*(1,91) = 21.14, *p* < 0.001, $\eta_{p}^{2}$ = 0.19, and a significant interaction between them, *F*(2,91) = 6.66, *p* = 0.002, $\eta_{p}^{2}$ = 0.13. Importantly, the proportion of choosing the high‐novelty option dropped significantly from training to testing in 4‐year‐olds, *p* < 0.001, but not in adults or 5‐ to 6‐year‐olds, *p*s > 0.661. The results indicated that eliminating direct access to perceptual novelty substantially reduced younger children's novelty preference.

Results from Bayesian multinomial logistic regression model also revealed a consistent preference for the high‐reward option in both adults and 5‐ to 6‐year‐olds in both training and testing. However, consistent with the ANOVA results, 4‐year‐olds showed a clear preference for the high‐novelty option only during training (as shown in the Supplemental Materials). This finding provided additional evidence that perceptual novelty alone could drive decisions in early development. However, such preference attenuated at test, when the option's perceptual novelty was only anticipated but not overtly displayed, suggesting that perceptual novelty might influence young children's choice decisions in a bottom‐up manner.

### 3.4 Model Fitting and Results

#### 3.4.1 Full Model Fitting and Results

The full model was fit independently to each participant's observed data with one more free parameter, $w_{n\_train}$, that captured the contribution of visible perceptual novelty in training.

We first conducted one‐way ANOVAs on the weights of all contributing factors, with age being the main effect for training and testing phases separately. We found significant age differences for all contributing factors in training: reward value, *F*(2,91) = 16.11, *p* < 0.001, $\eta_{p}^{2}$ = 0.26; objective uncertainty, *F*(2,91) = 3.683, *p* = 0.029, $\eta_{p}^{2}$ = 0.07; perceptual novelty, *F*(2,91) = 6.77, *p* < 0.01, $\eta_{p}^{2}$ = 0.13; and choice lag, *F*(2,91) = 3.562, *p* = 0.0324, $\eta_{p}^{2}$ = 0.07. Importantly, the weight of perceptual novelty was higher for 4‐year‐olds (*M*_4*yo*_ = 0.47, *SD*_4*yo*_ = 0.31) than 5‐ to 6‐year‐olds (*M*_5 − 6*yo*_ = 0.27, *SD*_5 − 6*yo*_ = 0.28) and adults (*M_adults_* = 0.25, *SD_adults_* = 0.19), *p*s *<* 0.01. No significant differences were found between adults and 5‐ to 6‐year‐olds, *p* > 0.95.

**FIGURE 6:**
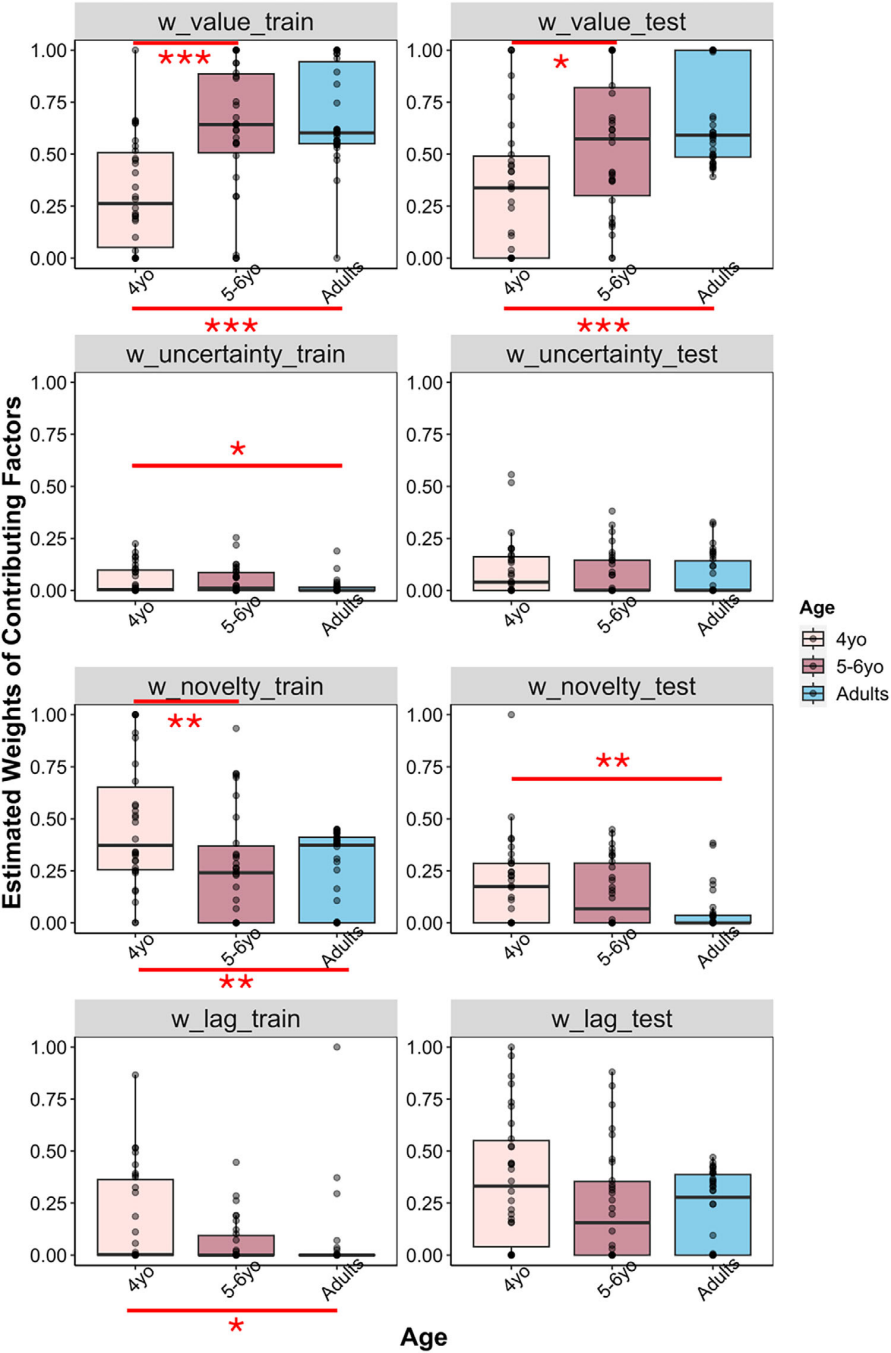
Distribution of best‐fitting values for all freely estimated factor weight parameters in the full model across age groups in Experiment 2.

For contributing factors in testing, we found a significant main effect of age for the weight of value, *F*(2,91) = 9.81, *p* < 0.001, $\eta_{p}^{2}$ = 0.18, anticipated perceptual novelty, *F*(2,91) = 6.385, *p* < 0.01, $\eta_{p}^{2}$ = 0.12, and choice lag, *F*(2,91) = 3.122, *p* = 0.0488, $\eta_{p}^{2}$ = 0.06. No significant age effect was found for the weight of objective uncertainty, *p* = 0.485. The weight of perceptual novelty was higher for 4‐year‐olds (*M*_4*yo*_ = 0.19, *SD*_4*yo*_ = 0.22) than adults (*M_adults_* = 0.05, *SD_adults_* = 0.10), *p <* 0.01, with no significant difference between 4‐year‐olds and 5‐ to 6‐year‐olds (*M*_5 − 6*yo*_ = 0.14, *SD*_5 − 6*yo*_ = 0.16) or between 5‐ and 6‐year‐olds and adults, *p*s > 0.06. Post hoc pairwise comparison results for other contributing factors in both phases are annotated in Figure 6 and reported in Supplemental Materials.

Critically, to examine differential impacts of observable versus anticipated perceptual novelty on participants’ choices, we conducted mixed ANOVA on the weight of perceptual novelty, with age being a between‐subject variable, and phase (or type of perceptual novelty) being a within‐subject variable. The results revealed a significant main effect of age, *F*(2,91) = 10.159, *p* < 0.001, $\eta_{p}^{2}$ = 0.18, and a significant main effect of phase, *F*(1,91) = 48.548, *p* < 0.001, $\eta_{p}^{2}$ = 0.35. The interaction did not reach significance, *p* = 0.127. The results demonstrated an overall important role of perceptual novelty in participants’ novelty‐based decisions across all age groups. Finally, similar to Experiment 1, we found age differences in log‐transformed λ (see Supplementary Materials for full analysis), with participants across all age groups being highly deterministic.

#### 3.4.2 Single‐Process Model Fitting and Results

**FIGURE 7:**
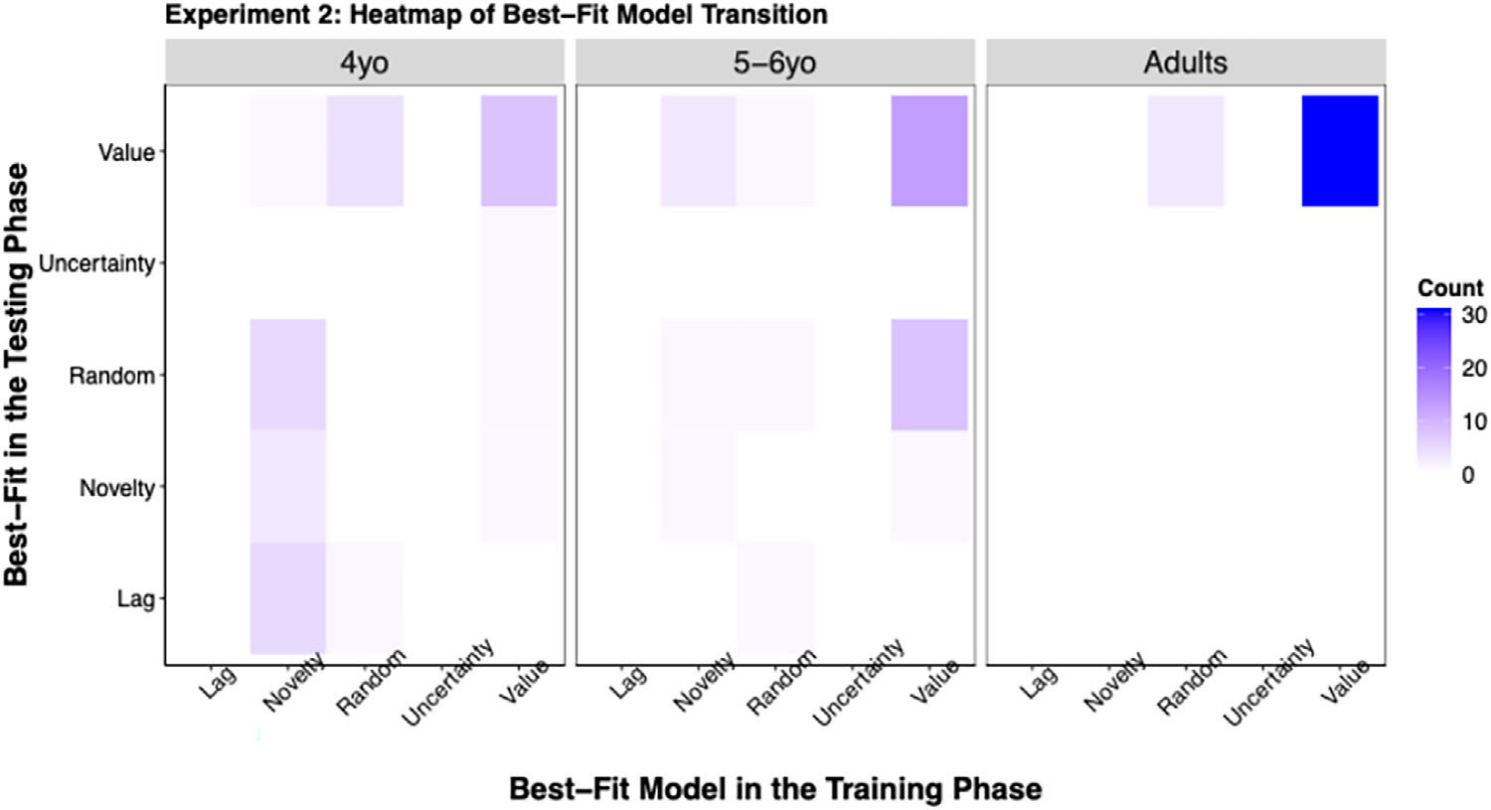
Heatmap of best‐fit model transition matrix in Experiment 2. The figure illustrates the transition from the best‐fit model in training to the best‐fit model in testing. Darker colors represent a higher frequency of transition.

We fit five single‐process models (i.e., value‐based, uncertainty‐based, novelty‐based, lag‐based, and random) for both training and testing. The frequencies of the best‐fit model in the training and testing phases, and the transition between them, are shown in Figure 7. Consistent with the goals of Experiment 2, we reported the analyses for only the novelty‐based Model in the main text. Results for the value‐based model can be found in Supplemental Materials.

The analyses on the novelty‐based model revealed that 14 out of 30 4‐year‐olds, 5 out of 30 5‐ to 6‐year‐olds, and none of the adults were best fit by the novelty‐based model in training. Pairwise Fisher's exact tests yielded significant differences between 4‐year‐olds and adults, *p* < 0.001, but no significant differences between 4‐year‐olds and 5‐ to 6‐year‐olds, *p* = 0.08, or between 5‐ and 6‐year‐olds and adults, *p* = 0.06.

As for testing, 4 out of 30 4‐year‐olds, 2 out of 30 5‐ to 6‐year‐olds, and none of the adults were best fit by the novelty‐based model. Pairwise Fisher's Exact tests found no significant differences between any two age groups, *p*s > 0.12. Consistent with our other findings in Experiment 2, the model comparison results demonstrated that perceptual novelty played a greater role in children's decisions when it could be directly observable.

Finally, most 4‐year‐olds who were best fit by the value‐based model in training continued to be best fit by the value‐based model in testing (8 out of 11). However, only a small fraction of 4‐year‐olds (3 out of 14) who were best fit by the novelty‐based model in training maintained novelty‐based in testing. This pattern suggested different underlying mechanisms for value‐based and novelty‐based decisions for 4‐year‐olds. Value‐based decision‐making appears to be controlled by a goal‐driven (or top‐down) mechanism, enabling children to maintain their strategy even when the reward information was obscured. By contrast, novelty‐based decision‐making seems to be driven by a stimulus‐driven (or bottom‐up) mechanism. Therefore, in the absence of perceptual novelty, children's tendency to utilize a novelty‐based strategy significantly decreased, indicating a reliance on external cues for this type of exploration.

### 3.5 Discussion

In Experiment 2, we fully decoupled perceptual novelty from all sources of epistemic uncertainty during training and found that perceptual novelty alone could sufficiently and independently drive 4‐year‐olds’, but not 5‐ to 6‐year‐olds’ or adults’ decisions. Particularly, although the high‐novelty option carried increased epistemic uncertainty in testing due to hidden information, 4‐year‐olds reduced their exploration of that option. This finding suggested that 4‐year‐olds were more driven by perceptual novelty than epistemic uncertainty.

Moreover, according to the single‐process model results, there is a critical distinction between value‐based and novelty‐based decisions: while the anticipation of high rewards can drive choices even in young children, the anticipation of novelty does not. This finding indicates that value‐based decision‐making is likely to be a strategic, goal‐directed, top‐down process, whereas novelty‐based decision is likely to be a stimulus‐driven bottom‐up process, as it was highly dependent on the presence of direct perceptual novelty.

Furthermore, developmental findings in Experiment 2 differed somewhat from those in Experiment 1: in Experiment 1, the two child age groups were similar to each other, whereas in Experiment 2, 5‐ to 6‐year‐olds’ decision‐making was more similar to that of adults. In the General discussion, we discussed this further and provided some potential explanations.

## 4 General Discussion

The reported research examined the role of perceptual novelty in decision‐making by fully decoupling perceptual novelty from epistemic uncertainty. The results indicate that perceptual novelty is critical, and, by itself, sufficient to drive choices in early development, likely in a bottom‐up manner.

Previous research has suggested that novelty can drive choices. For example, studies have shown that novelty is inherently rewarding as dopaminergic neurons that code reward‐prediction errors also respond to stimulus novelty (Düzel et al. 2010; Gottlieb 2012; Kakade and Dayan 2002; Wittmann et al. 2008), and novel stimuli can activate dopamine cells even when they are not objectively uncertain or predictive of rewards (Düzel et al. 2010; Kakade and Dayan 2002). This suggests that perceptual novelty may serve as an independent driver of exploration unrelated to other choice attributes. However, in most previous studies, novelty was confounded with epistemic uncertainty. The current study extends these findings by decoupling novelty and uncertainty and providing new evidence that, in early development, perceptual novelty alone can drive choices.

Given that perceptual novelty drives young children's decisions even in the absence of epistemic uncertainty, and that its impact is contingent on its being explicitly present, children's novelty‐based decisions are presumably a stimulus‐driven, bottom‐up process. Such results aligned with previous findings that the presence of a salient choice disrupted children's systematic exploration, implying an important role of attention in early exploration (Blanco and Sloutsky 2020).

If a perceptually novel stimulus automatically captures attention, why does it predominate only in young children? The protracted development of cognitive control may explain this difference. Adults, with mature cognitive control, can suppress the attractiveness of the novel option, and direct their attention to the most rewarding option. In contrast, young children with immature cognitive control, are more prone to attention being automatically captured by perceptually novel stimuli, and experiencing more difficulty disengaging from them (Gaspelin et al. 2015; Hayre et al. 2022).

To note, our findings that perceptual novelty drives young children's decisions in a bottom‐up manner do not contradict the notion that novelty‐based decisions can be guided by a top‐down process. Due to the common confound of novelty and epistemic uncertainty in real‐world situations, children's novelty‐seeking behaviors are very likely to stem from both a top‐down process to reduce uncertainty and a bottom‐up attentional capture. Additionally, children may simply pursue their self‐established goals to find enjoyment in novel experiences. Our results are not incompatible with these possibilities. Rather, they complement these perspectives by providing new evidence that young children's novelty‐based decisions can also arise from a bottom‐up process.

Finally, we noticed that despite evident age differences between 4‐year‐olds and adults in both experiments, 5‐ to 6‐year‐olds’ decision‐making was more comparable to 4‐year‐olds in Experiment 1, but more comparable to adults in Experiment 2. Specifically, 5‐ to 6‐year‐olds were more novelty‐based than adults at the test stage of Experiment 1, but were no different from adults in their high‐novelty choices during the training of Experiment 2. These differences may reflect the interaction between the development of attentional mechanisms and exploration‐exploitation strategies. On the one hand, 5‐ to 6‐year‐olds with relatively more mature attentional control than 4‐year‐olds, were less susceptible to automatic attention capture by the perceptually novel option, resulting in a reduction in selecting the high‐novelty option in Experiment 2. On the other hand, as found in previous studies, 5‐ to 6‐year‐old's decision‐making could be substantially influenced by choices’ epistemic uncertainty (Blanco and Sloutsky 2020, 2021; Meder et al. 2021). Therefore, a novel option in conjunction with some epistemic uncertainty of rewards (as was the case in Experiment 1 testing) likely dominated their choices.

### 4.1 Limitations and Future Directions

One potential limitation of the study is the assumption of nonnegative weights for all factors influencing participants’ choice decisions in the model construction. This assumption may not always hold, particularly given previous findings of uncertainty aversion in adults (Cockburn et al. 2022; Nussenbaum et al. 2023). However, since the primary focus of this study was on young children, who did not exhibit uncertainty aversion, we do not consider this limitation as a critical concern.

Additionally, although the current research provides substantial evidence for a link between attentional mechanisms and exploration in early development, it did not directly measure participants’ attentional orientation during the tasks. Therefore, future research is expected to use eye‐tracking to directly measure children's overt attention to provide more evidence for the role of attention in early exploration and how the development of attention shapes the emergence of an exploitation tendency.

## 5 Conclusion

The current research investigated the contribution of perceptual novelty, in the absence of epistemic uncertainty, to exploration in early development. By fully disentangling perceptual novelty and all forms of epistemic uncertainty, we provided novel evidence that 4‐year‐olds’ decisions, unlike adults’ and older children's, could be driven solely by perceptual novelty even when it was not associated with uncertainty or rewards. Additionally, we found that a novel option predominated in children's choices only when its novelty was directly observable, indicating that perceptual novelty drives early choices in a bottom‐up manner.

## Ethics Statement

Approval was obtained from the ethics committee of the Ohio State University. The authors give permission to reproduce material from other sources.

## Conflicts of Interest

The authors declare no conflicts of interest.

## Supporting information



Supporting Information

## Data Availability

Stimuli, data, and analysis code used for this article are available via the Open Science Framework and can be accessed via the link: https://osf.io/bvdc6/?view_only=17a18d85328941f299a61e8b17a09997.

## Endnotes

1 We ran the same analyses for high‐uncertainty and low‐all options in both phases as well and annotated the statistical significance of the results in Figure 2.

2 Same as in Experiment 1, we ran one‐way ANOVAs for High‐Uncertainty and Low‐All options in both phases as well and annotated the statistical significance of the results in Figure 5.
