# Supplementary material for: Perceptual Novelty Drives Early Exploration in a Bottom‐Up Manner
Source: Dev Sci. 2025 Mar 3;28(3):e70002. doi: 10.1111/desc.70002 (PMC11876794; doi:10.1111/desc.70002)
Supplement: Supplementary file 1 — Supporting Information [file DESC-28-e70002-s001.docx]

**Supplemental Materials**

*Supplemental Table 1*

*Feedback provided for children in Experiments 1 and 2.* $R_{i,t}$ represents the reward for option *i* at trial *t.*

| Reward Value | High-Novelty Option? | Feedback |
| --- | --- | --- |
| $R_{i,t}=0$ | No | Oops! You did not get coin this time from the animal friend on this color box, and you cannot move the unicorn.  You did not get coins, so you cannot move the unicorn. |
|  | Yes | Oops! You did not get coin this time from the animal friend on this color box, and you cannot move the unicorn. **And you met a different animal friend on this color box!**  You did not get coins, so you cannot move the unicorn. |
| $0<R_{i,t} \leq5$ | No | Oops! This time, the animal friend on this color box gave you only a small amount of coins and you can move the unicorn very little.  You got XX coins and let’s press the yellow button XX times to move the unicorn. |
|  | Yes | Oops! This time, the animal friend on this color box gave you only a small amount of coins and you can move the unicorn very little. **And you met a different animal friend on this color box!**  You got XX coins and let’s press the yellow button XX times to move the unicorn. |
| $5<R_{i,t} \leq15$ | No | This time, the animal friend on this color tox gave you some coins and you can move the unicorn a bit!  You got XX coins and let’s press the yellow button XX times to move the unicorn. |
|  | Yes | This time, the animal friend on this color tox gave you some coins and you can move the unicorn a bit! **And you met a different animal friend on this color box!**  You got XX coins and let’s press the yellow button XX times to move the unicorn. |
| $R_{i,t}>15$ | No | Awesome! This time, the animal friend on this color box gave you a lot of coins and you can move the unicorn a lot!  You got XX coins and let’s press the yellow button XX times to move the unicorn. |
|  | Yes | Awesome! This time, the animal friend on this color box gave you a lot of coins and you can move the unicorn a lot! **And you met a different animal friend on this color box!**  You got XX coins and let’s press the yellow button XX times to move the unicorn. |

*Supplemental Table 2. Binomial Test Results for Correct Responses to High-Reward and High-Novelty Memory Questions Across Age Groups in Experiment 1. The table displays the percentage of correct responses, chance-level proportions, p-values, and 95% confidence intervals for High-reward and High-novelty memory questions among 4-year-olds and 5- to 6-year-olds.*

| **Memory Question** | **Age Group** | **Percentage of Correct Responses** | **Chance-Level Proportion** | ***p*-value** | **95% Confidence Interval** |
| --- | --- | --- | --- | --- | --- |
| High-Reward | 4-year-olds | 0.533 (16 out of 30) | 0.25 | < 0.001 | (0.370, 1) |
|  | 5- to 6-year-olds | 0.75 (24 out of 32) | 0.25 | < 0.001 | (0.594, 1) |
| High-Novelty | 4-year-olds | 0.5 (15 out of 30) | 0.25 | < 0.003 | (0.339, 1) |
|  | 5- to 6-year-olds | 0.719 (23 out of 32) | 0.25 | < 0.001 | (0.561, 1) |

*Supplemental Table 3. Binomial Test Results for Correct Responses to High-Reward, High-Novelty, and High-Uncertainty Memory Questions Across Age Groups in Experiment 2. The table displays the percentage of correct responses, chance-level proportions, p-values, and 95% confidence intervals for High-Reward, High-Novelty, and High-Uncertainty memory questions among 4-year-olds and 5- to 6-year-olds.*

| **Memory Question** | **Age Group** | **Percentage of Correct Responses** | **Chance-Level Proportion** | ***p*-value** | **95% Confidence Interval** |
| --- | --- | --- | --- | --- | --- |
| High-Reward | 4-year-olds | 0.5 (15 out of 30) | 0.25 | < 0.003 | (0.339, 1) |
|  | 5- to 6-year-olds | 0.767 (23 out of 30) | 0.25 | < 0.001 | (0.606, 1) |
| High-Novelty | 4-year-olds | 0.433 (13 out of 30) | 0.25 | < 0.05 | (0.279, 1) |
|  | 5- to 6-year-olds | 0.633 (19 out of 30) | 0.25 | < 0.001 | (0.467, 1) |
| High-Uncertainty | 4-year-olds | 0.333 (10 out of 30) | 0.25 | = 0.197 | (0.193, 1) |
|  | 5- to 6-year-olds | 0.3 (9 out of 30) | 0.25 | = 0.326 | (0.167, 1) |

*Supplemental Table 4. Proportion of choosing the "best" presented option and the High-Reward option across age groups during the training phase. The table also includes p-values from paired t-tests comparing the proportions of the "best" presented option and the High-Reward option for each age group. The results (all ps < 0.001) indicated that participants across all age groups were maximizing rewards based on presented information, instead of simply pressing the same button repeatedly.*

| **Experiment** | **Age Group** | **“Best” Option Proportion** | **High-Reward Option Proportion** | ***p-*value** |
| --- | --- | --- | --- | --- |
| Experiment 1 | Adults | 0.9396 (0.0857) | 0.8250 (0.1194) | < 0.001 |
|  | 5- to 6-year-olds | 0.7427 (0.1977) | 0.6531 (0.1630) | < 0.001 |
|  | 4-year-olds | 0.6356 (0.1811) | 0.5811 (0.1970) | < 0.001 |
| Experiment 2 | Adults | 0.8814 (0.1670) | 0.7745 (0.1574) | < 0.001 |
|  | 5- to 6-year-olds | 0.6589 (0.3323) | 0.5889 (0.2967) | < 0.001 |
|  | 4-year-olds | 0.3856 (0.2818) | 0.3267 (0.2566) | < 0.001 |


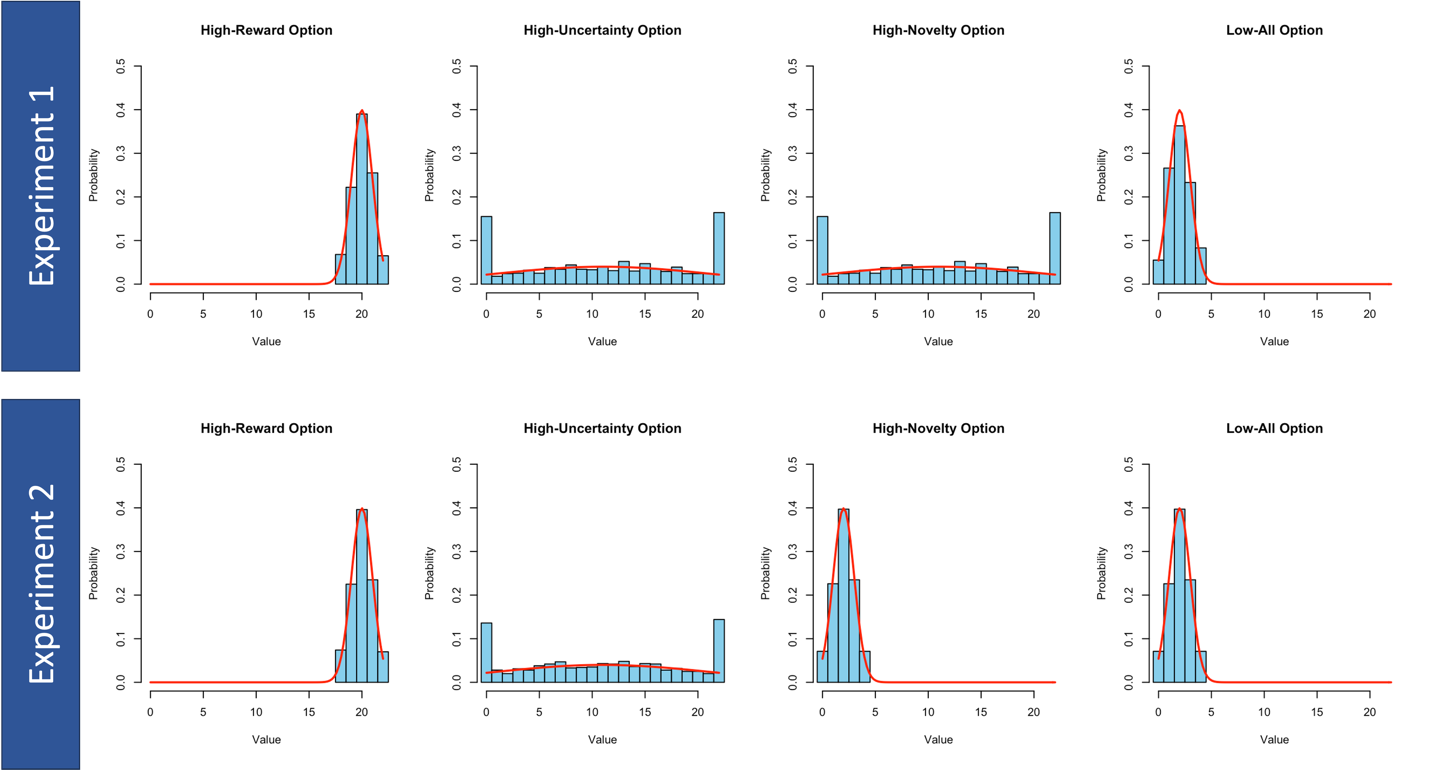


*Supplemental Figure 1*. Approximate reward distribution for four options in Experiment 1 (top row) and Experiment 2 (bottom row). The reward distribution for each option was plotted by simulating 1000 values based on the pre-determined distributions and upper and lower bounds of each option (as described in the text).

**Additional Procedural Details for Experiment 1 and Experiment 2**

The experiments were programmed in Matlab (The MathWorks, Inc.) using the Psychophysics Toolbox Version 3. In Experiment 1, we pre-selected three animal images (chinchilla, alpaca, and panda), and three colors (blue, green, and yellow) to be associated with familiar options across all participants. In Experiment 2, we pre-selected one more color, pink. Same as Experiment 1, the assignment of colors and animal images to options were randomized across participants, and the color-animal pairs remained constant for non-novelty options throughout the experiment.

Moreover, we introduced several procedural changes to the child version of the experiment to help them better understand the instructions and be more engaged with the task. First, to help children to link virtual coins to more tangible rewards, we instructed them to press a yellow button on a custom-made game box to "collect" coins following each choice, and the number of button presses matched the number of coins they gained (e.g., if they pressed the button 20 times they would gain 20 coins). Children were told that the collected coins could make a unicorn (as shown in Figure 1 in the main text) move forward, and as long as the unicorn passed each checkpoint, they could gain one sticker (a tangible reward), and their goal was to collect as many stickers as possible. Although we checked each child’s numerical understanding at the beginning of the experiment (i.e., pointed to a number of ‘11’ on the screen and asked children what the number was) and had experimenters read the numbers to children on each trial if they had difficulty with number reading, instructing them to press buttons could further help them learn rewards associated with each choice.

Additionally, the verbal feedback displayed beneath the animal images during the feedback was different for adults and children. Adults received explicit feedback about the exact number of rewards gained from the chosen option (e.g., “You collected 20 coins from the animal friend on this color box”). However, for children, the verbal feedback not only informed them of the precise number of rewards and linked it to the move of the unicorn (e.g., You got 20 coins, and let’s press the yellow button 20 times to move the unicorn) but also used quantifiers (i.e., a lot of, some, a small amount of) based on the number of rewards they collected. Since additional information and actions (button pressing) related to the reward value were given, we also provided additional reminders regarding the stimulus novelty. The detailed feedback was provided in Supplemental Materials Table 1.

Finally, adults completed the experiment on a laboratory computer and read all the instructions and feedback on their own. They responded by clicking animal pictures (or colored squares) using a mouse and proceeded by clicking a “continue” button on the screen. In contrast, children were tested on a touchscreen display, with an experimenter delivering all the instructions and feedback to them. Children responded by directly pressing animal pictures (or colored squares) on the touchscreen using their fingers.

**Technical Details of the Full Model**

As mentioned in the main text, computational modeling was employed to examine the differential impacts of option attributes on children’s and adults’ choice decisions. The model assumed that participants’ choices are influenced by (expected) reward values, reward variability, perceptual novelty, and choice lag, and adopted a Bayesian updating mechanism for learning the mean and variance of reward distributions.

It is important to note that the model was not a cognitive model that attempted to explain or predict participants’ cognitive processes when performing the task. Instead, it was used as an analytical tool to evaluate the contribution of options’ attributes (i.e., novelty, uncertainty, etc.) to participants’ decisions. Possibly, participants did not update their learning of reward distributions via a Bayesian updating mechanism due to cognitive constraints. However, the Bayesian updating mechanism allowed us to investigate whether participants’ choices could be driven by expected reward values and objective uncertainty when they were perfectly learned. If these factors did not influence participants’ choices in our task even when learned perfectly, we would not expect them to play a role when they were learned less than perfectly.

To implement Bayesian updating mechanism, we applied a Normal-Gamma prior, $p\left( \mu, \tau\right)\sim NG(\mu^{'}, \lambda, \alpha, \beta)$, for each option and assumed that participants’ beliefs about the mean and variance of each reward distribution are updated as obtaining new evidence either from direct observation (in the training phase) or through their choices (in the testing phase). Due to the capping approach we used to draw reward values, the resulting distributions for option rewards deviate from true normal distributions. However, we do not consider it as a concern given that the expected means and variances learned by the model were monotonically related to the true means and variances for the four option reward distributions.

The expected reward values for all options except the High-Novelty Option in Experiment 1 were initialized as the observed values on the first training trial and were updated from the second trial. The expected reward value for the High-Novelty Option in Experiment 1 that was introduced in the testing phase was initialized as the average of the expected reward values of the three familiar options at the end of the training phase. The expected reward value ($\mu_{i,t+1}^{'}$) for each option is updated by Supplemental Equation 1:

$\mu_{i,t+1}^{'}= \frac{\lambda_{i,t}\mu_{i,t}^{'}+ R_{i,t}}{\lambda_{i,t}+1}$ 1

where $\lambda_{i,t}$ controls the influence of prior observed rewards on current expectations on each trial, balancing new evidence and established beliefs. In our model, we interpreted $\lambda_{i,t}$ as the number of observations, and therefore, is initialized to 0 at the beginning, and updated whenever a new observation was obtained:

$\lambda_{i,t+1}= \lambda_{i,t}+1$ 2

The objective uncertainty for each option was indexed by the standard deviation of the posterior reward distribution of the option, calculated by Supplemental Equation 3:

$\sigma_{i,t}= \sqrt{\frac{\beta_{i,t}}{{\lambda_{i,t}(\alpha}_{i,t}-1)}}$ 3

where $\alpha_{i,t}$ and $\beta_{i,t}$ are parameters of Gamma distribution, jointly modeling the precision ($\tau$, inverse of variance) of reward values. They were initialized at 1 and updated by Supplemental Equation 4, and Equation 5, respectively.

$\alpha_{i,t+1}= \alpha_{i,t}+ \frac{1}{2}$ 4

$\beta_{i,t+1}= \beta_{i,t}+ \frac{\lambda_{i,t}{(R_{i,t}- \mu_{i,t}^{'})}^{2}}{2(\lambda_{i,t}+1)}$ 5

Furthermore, parameters are updated for all options in the training phase. However, only parameters for the chosen options were updated in the testing phase.

To ensure parameter identifiability and maintain model parsimony, we set the initial values of $\lambda$, $\alpha$, and $\beta$ of the Normal-Gamma distribution priors as 0, 1, and 1, respectively, for all options and all participants ($\lambda_{i,1}$ = 0, $\alpha_{i,1}$ = 1, $\beta_{i,1}$ = 1).

**Supplemental Analyses**

**Experiment 1**

**Bayesian Multinomial Test on Choice Proportion**

A Bayesian multinomial logistic regression model was fit to each individual participant’s choices during test using the rstan package in R to estimate the posterior distributions of the probabilities for each option (Stan Development Team, 2020). The model fitting results for each participant is shown in Supplemental Figure 2 (4-year-olds), Supplement Figure 3 (5- to 6-year-olds), and Supplemental Figure 4 (adults). As shown in the figure, the High-Novelty option dominated the choice for most 4-year-olds (approximately 22 out of 30) and 5- to 6-year-olds (approximately 18 out of 32). On the contrary, most adults showed a markedly higher probability of choosing the High-Reward option compared to all the other options (26 out of 32). The Bayesian multinomial test yielded consistent results with our full model and single process model fitting results, supporting a value-based decision in adults and a novelty-based decision in children.


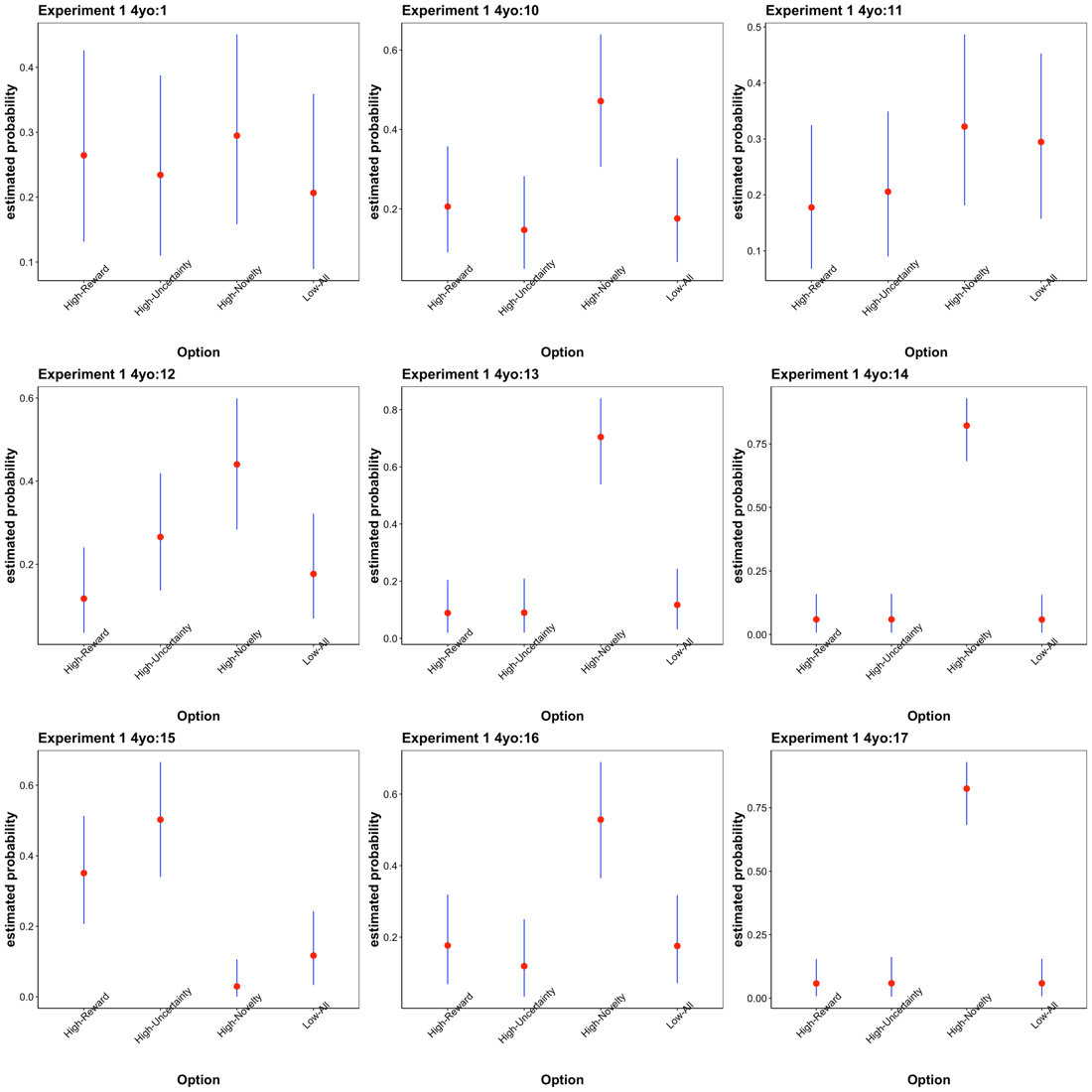

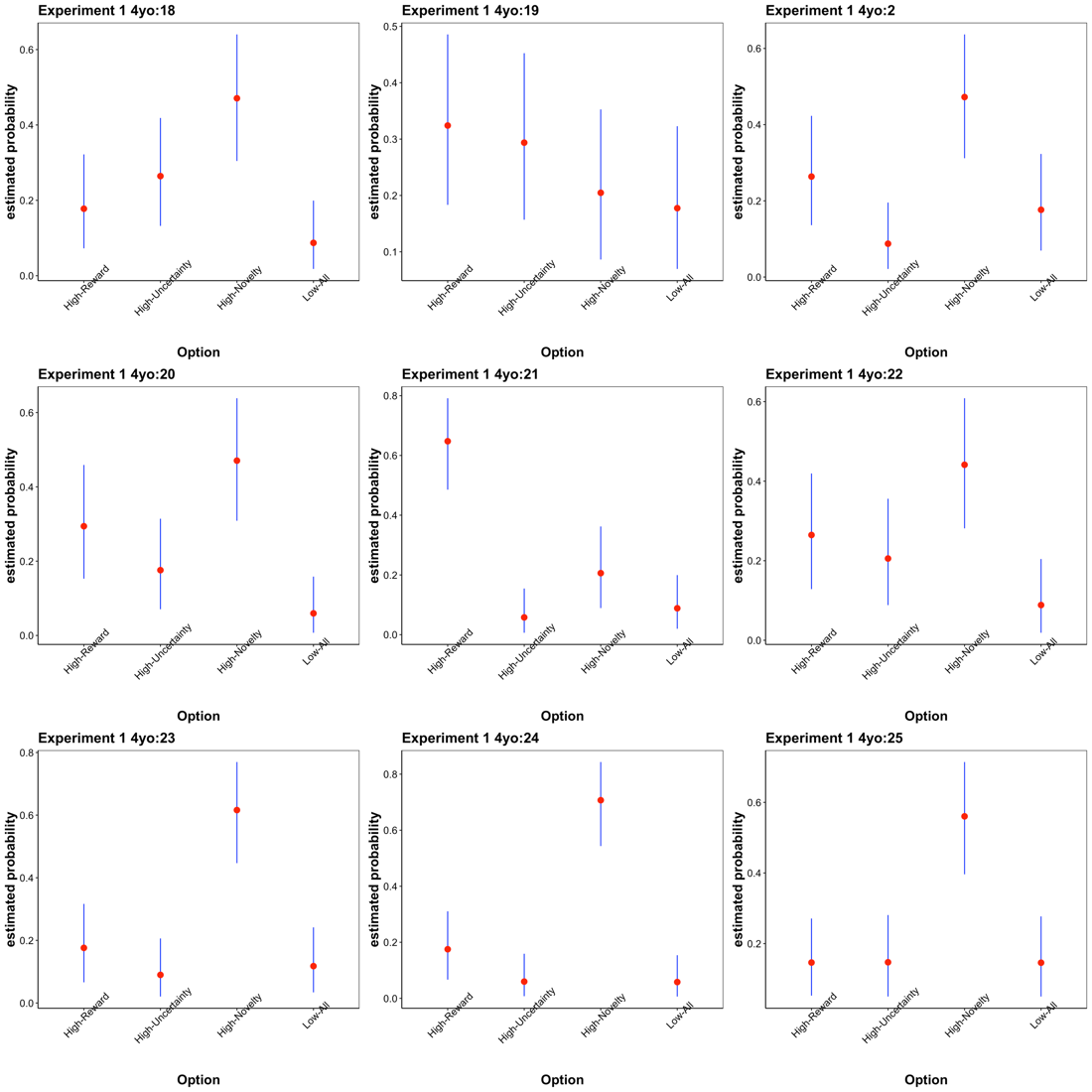

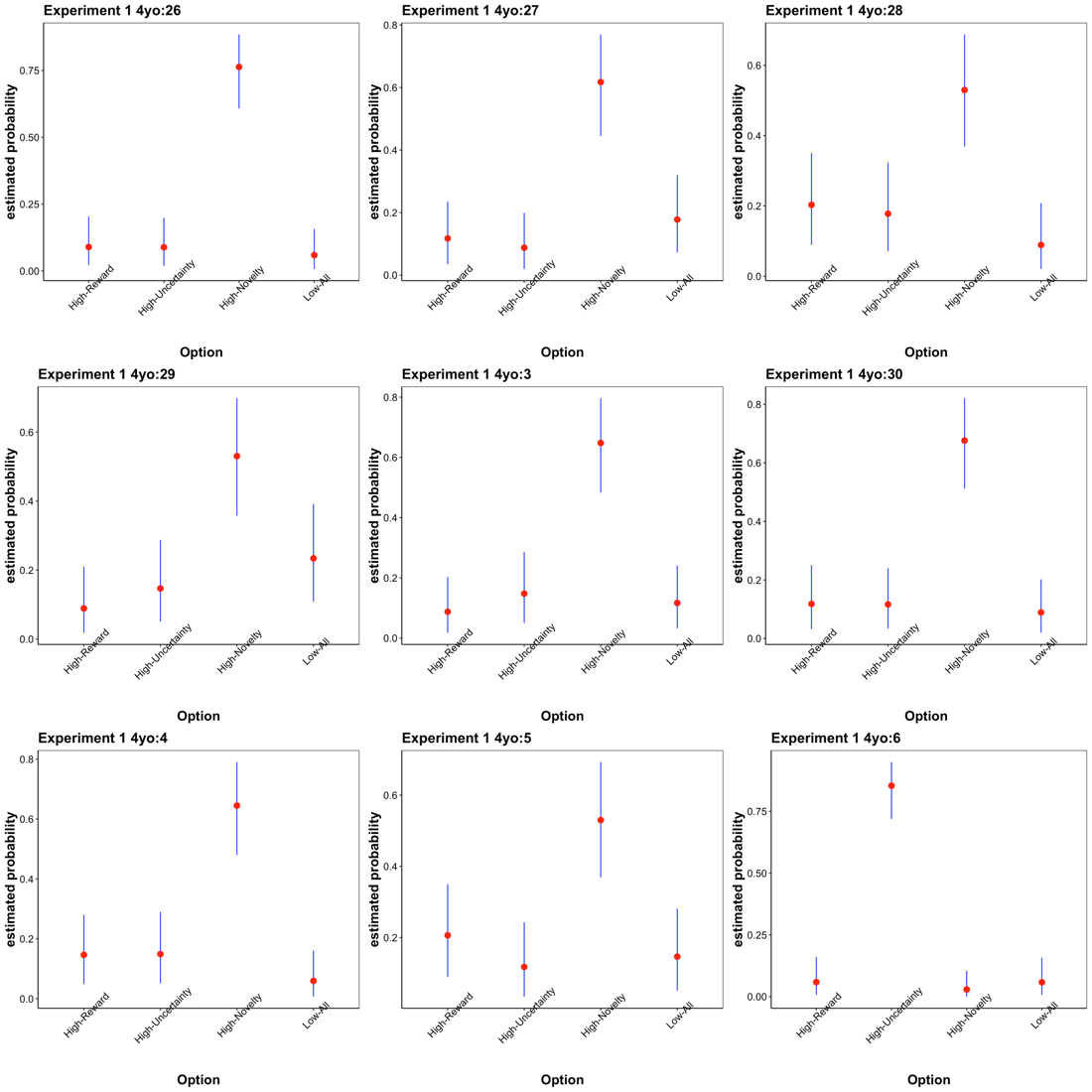

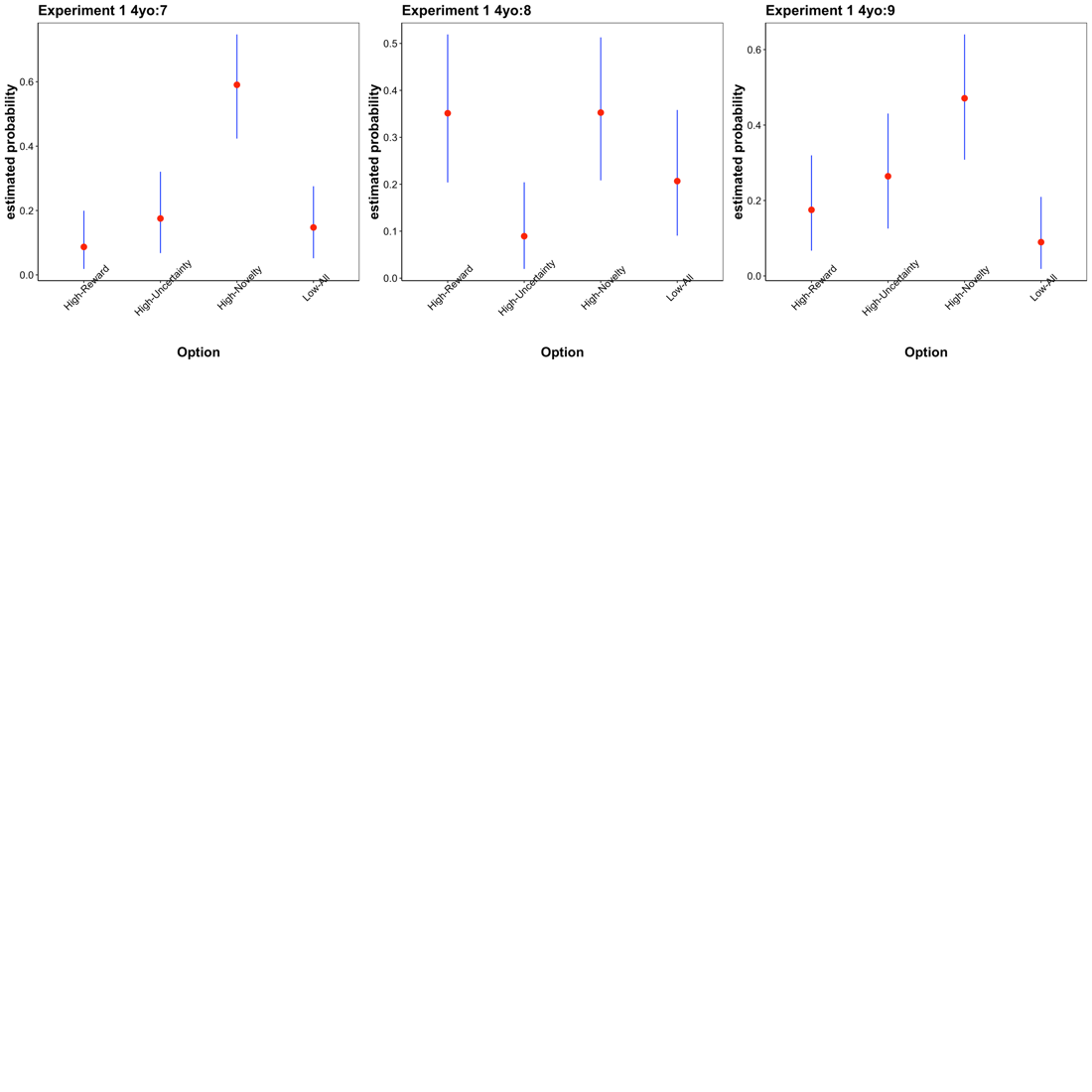


*Supplemental Figure 2*. Posterior Means and 95% credible intervals for the probabilities of choosing each option for 4-year-olds in Experiment 1 testing.


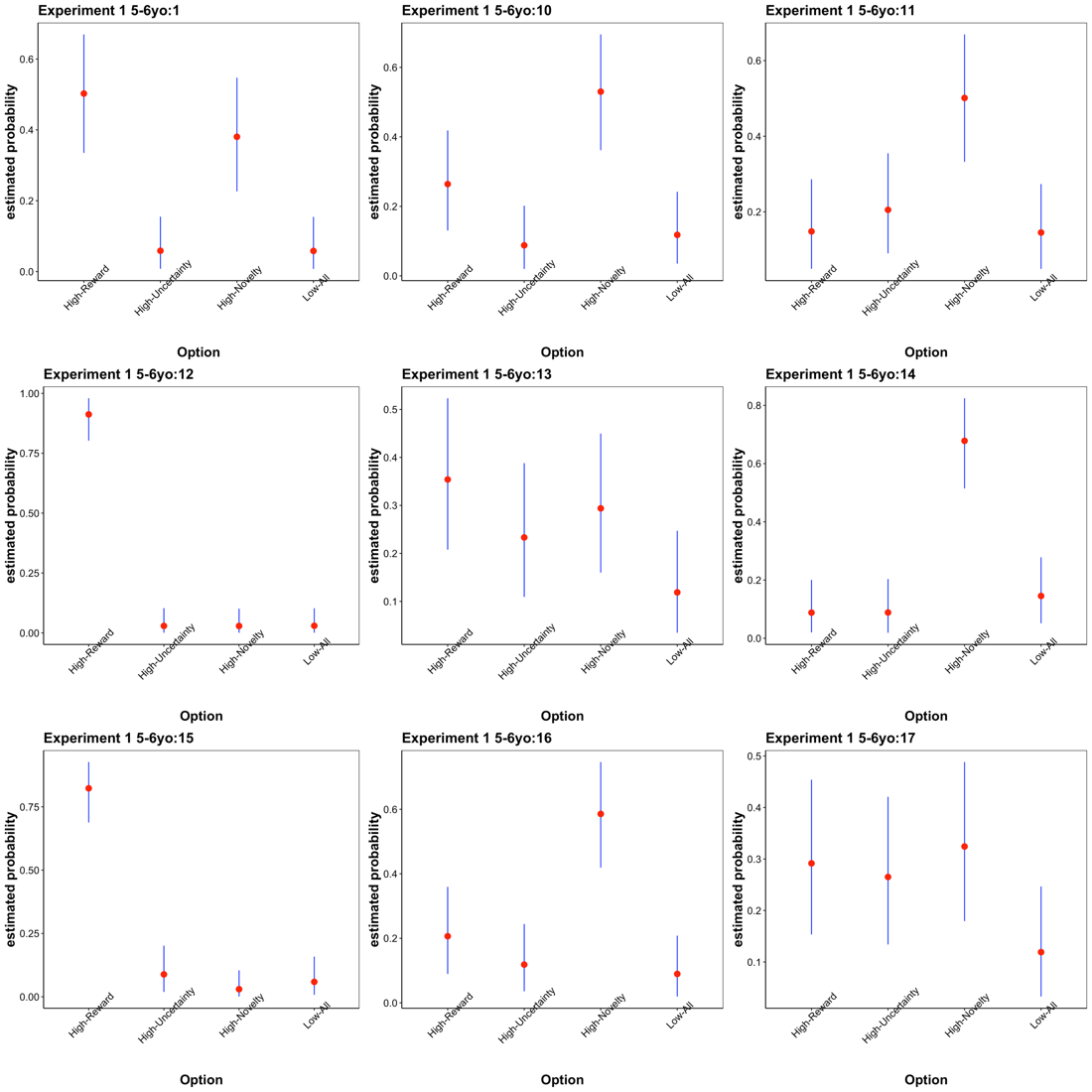

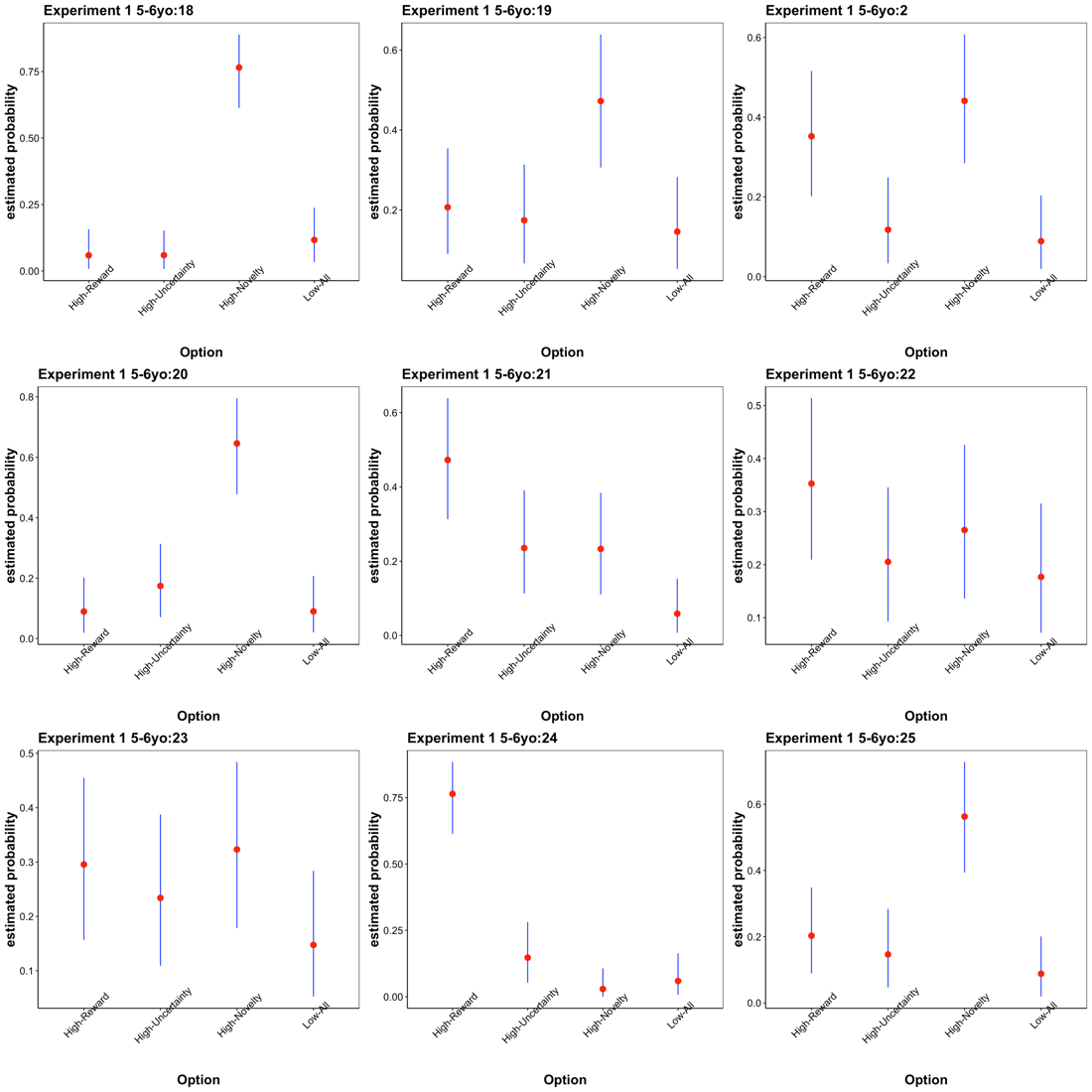

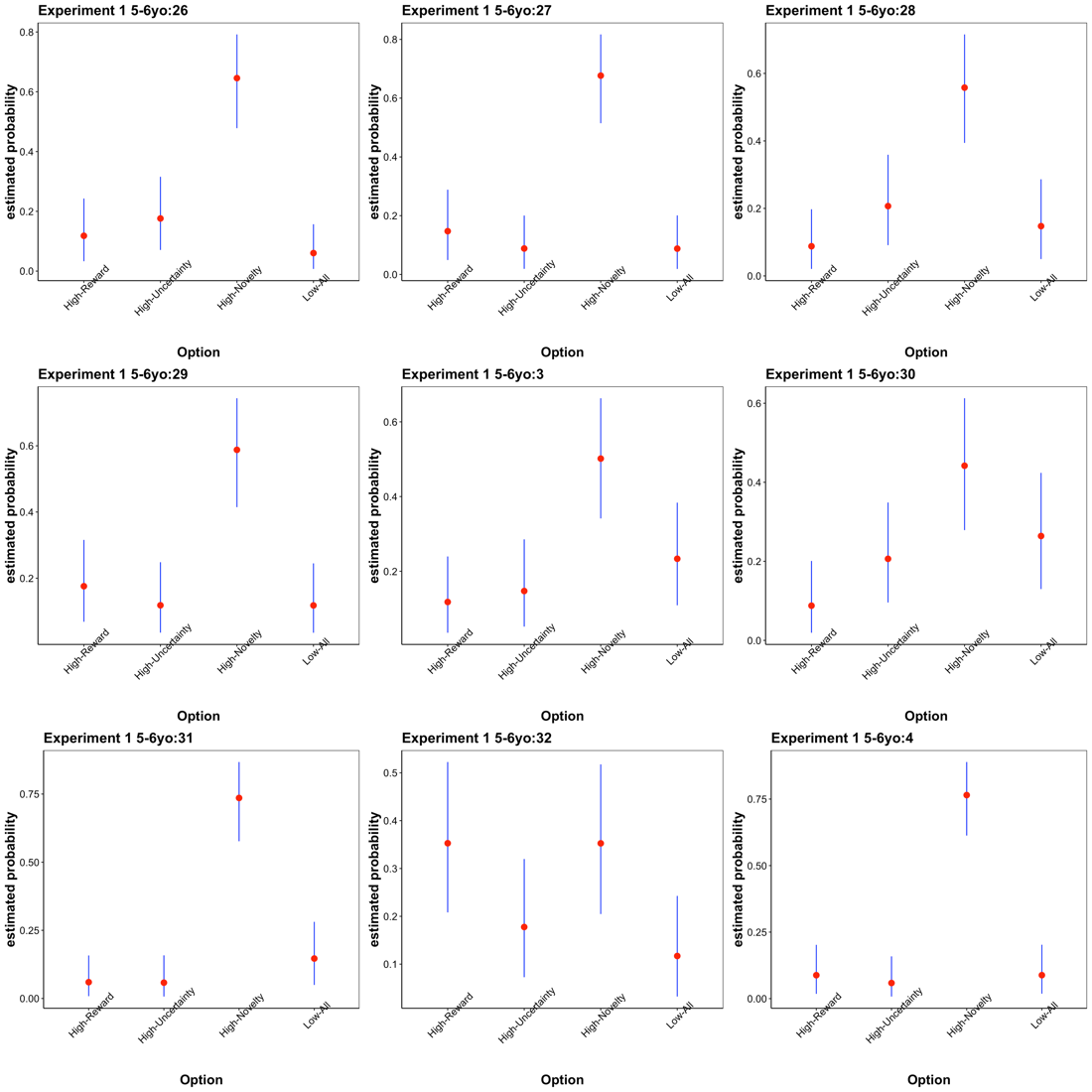

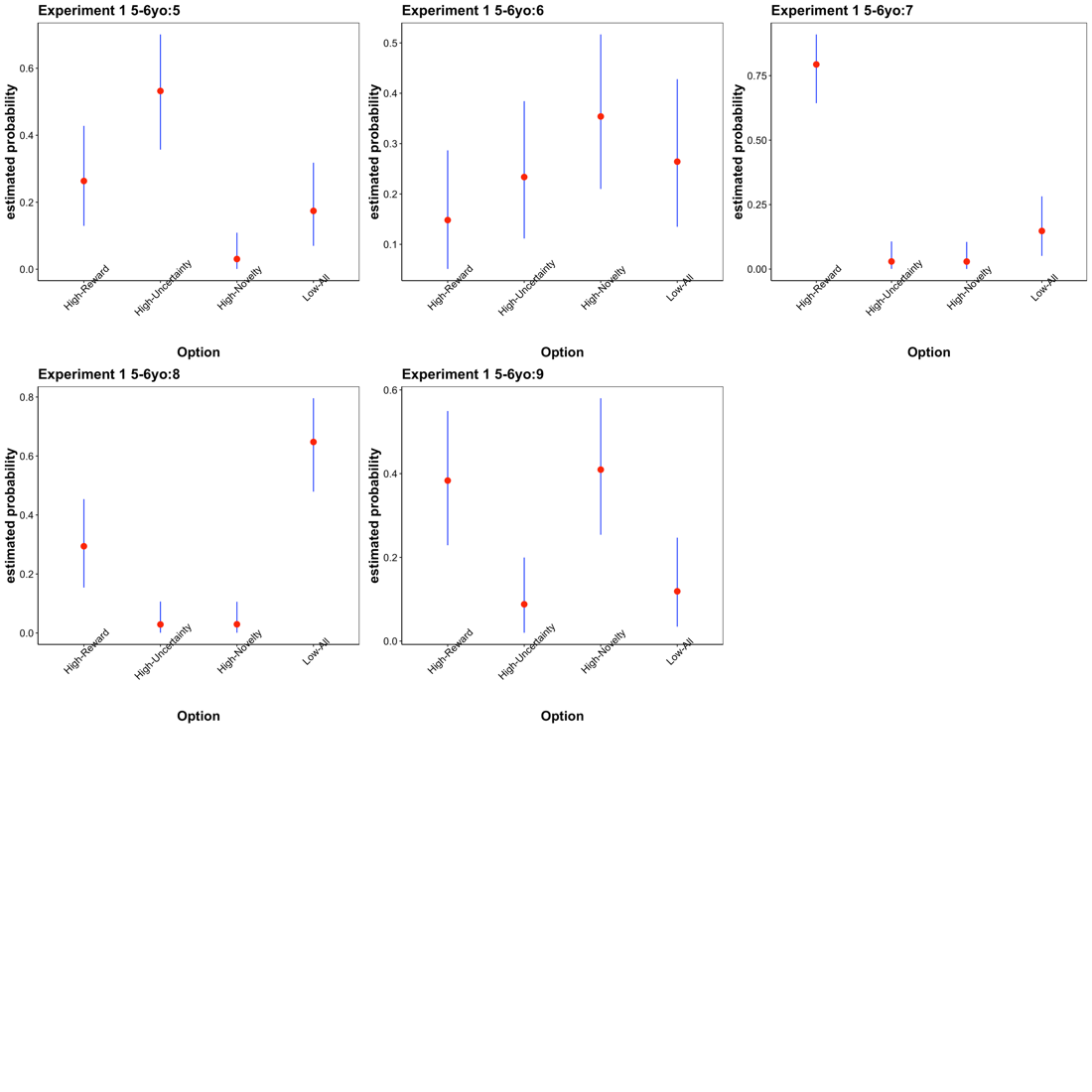


*Supplemental Figure 3*. Posterior Means and 95% credible intervals for the probabilities of choosing each option for 5- to 6-year-olds in Experiment 1 testing.


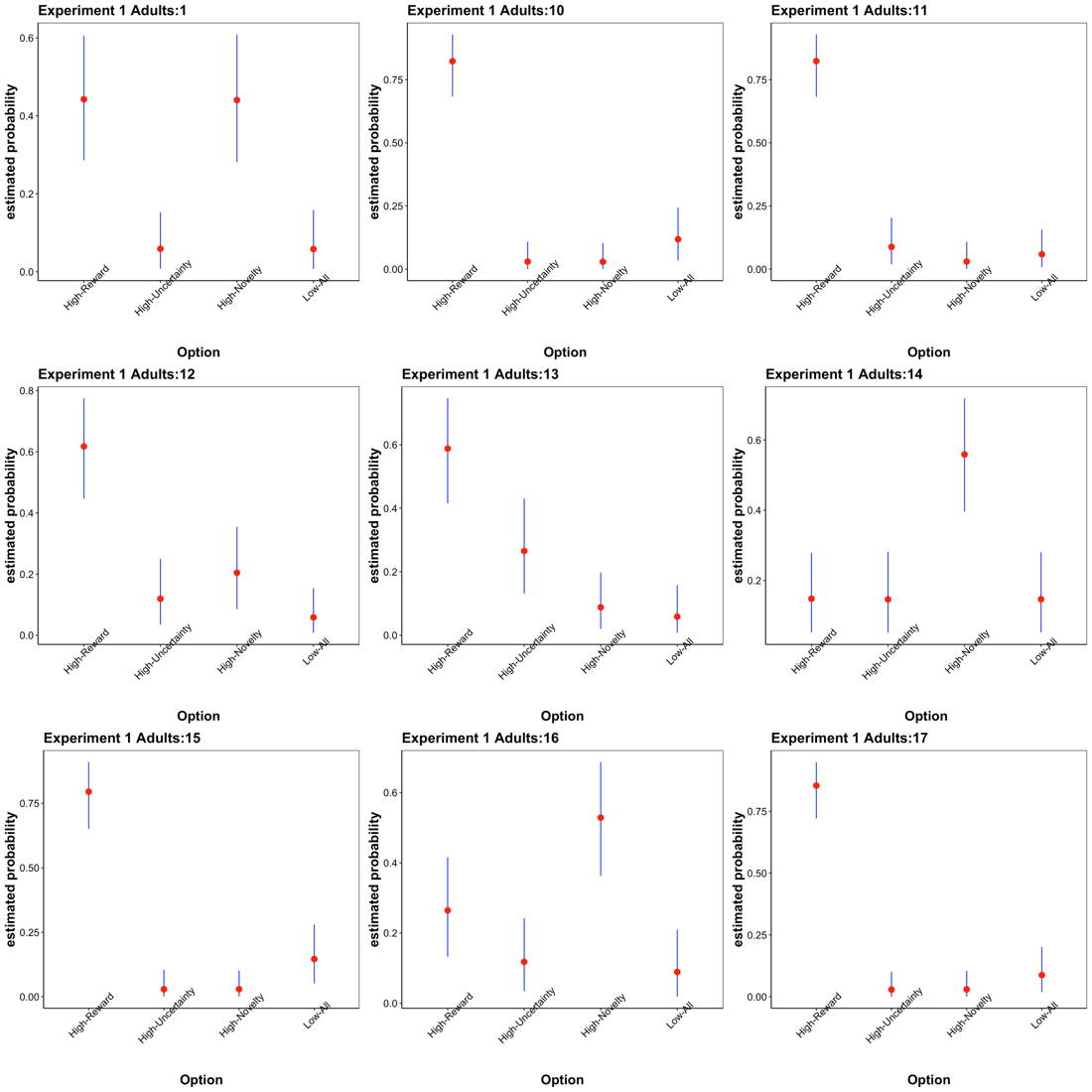


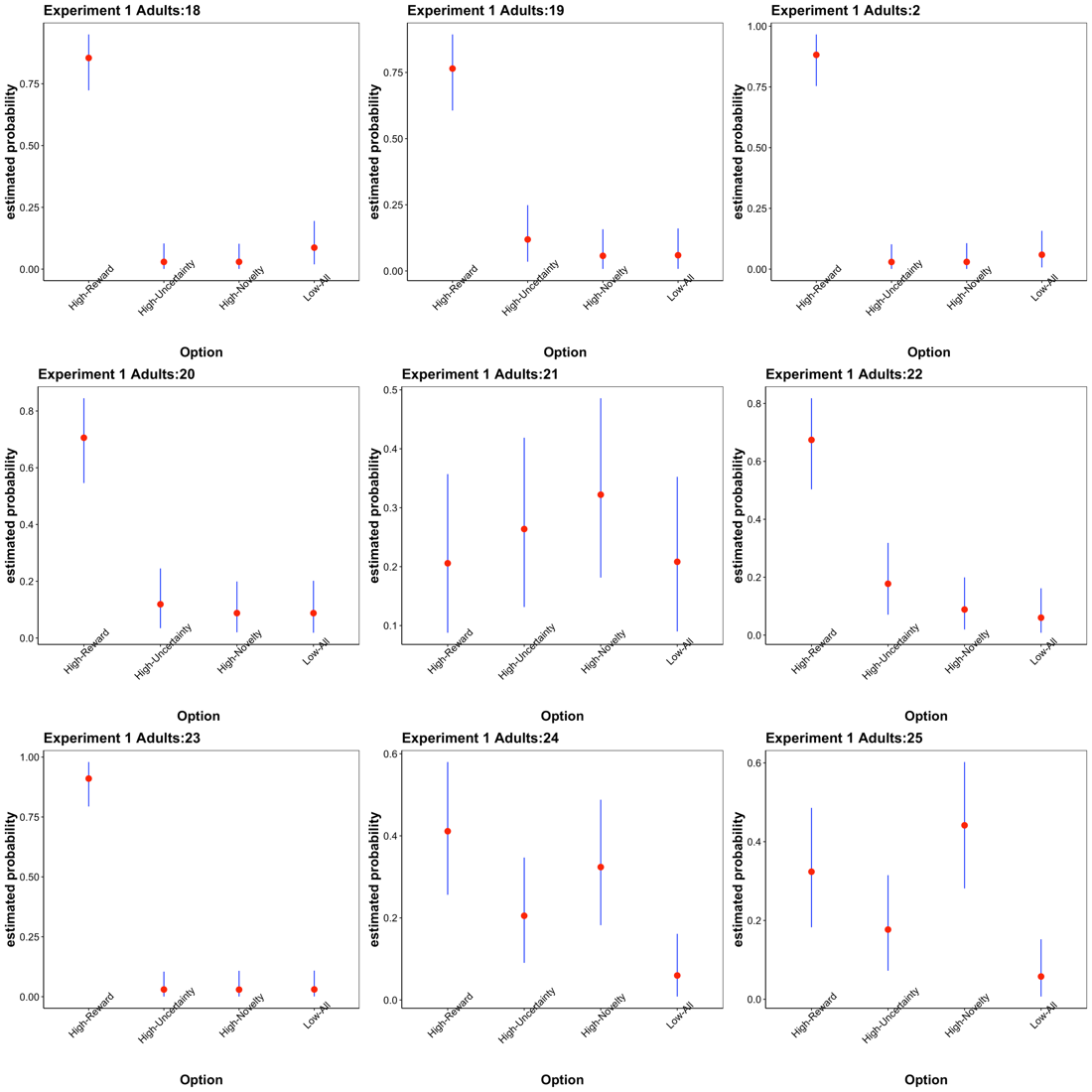

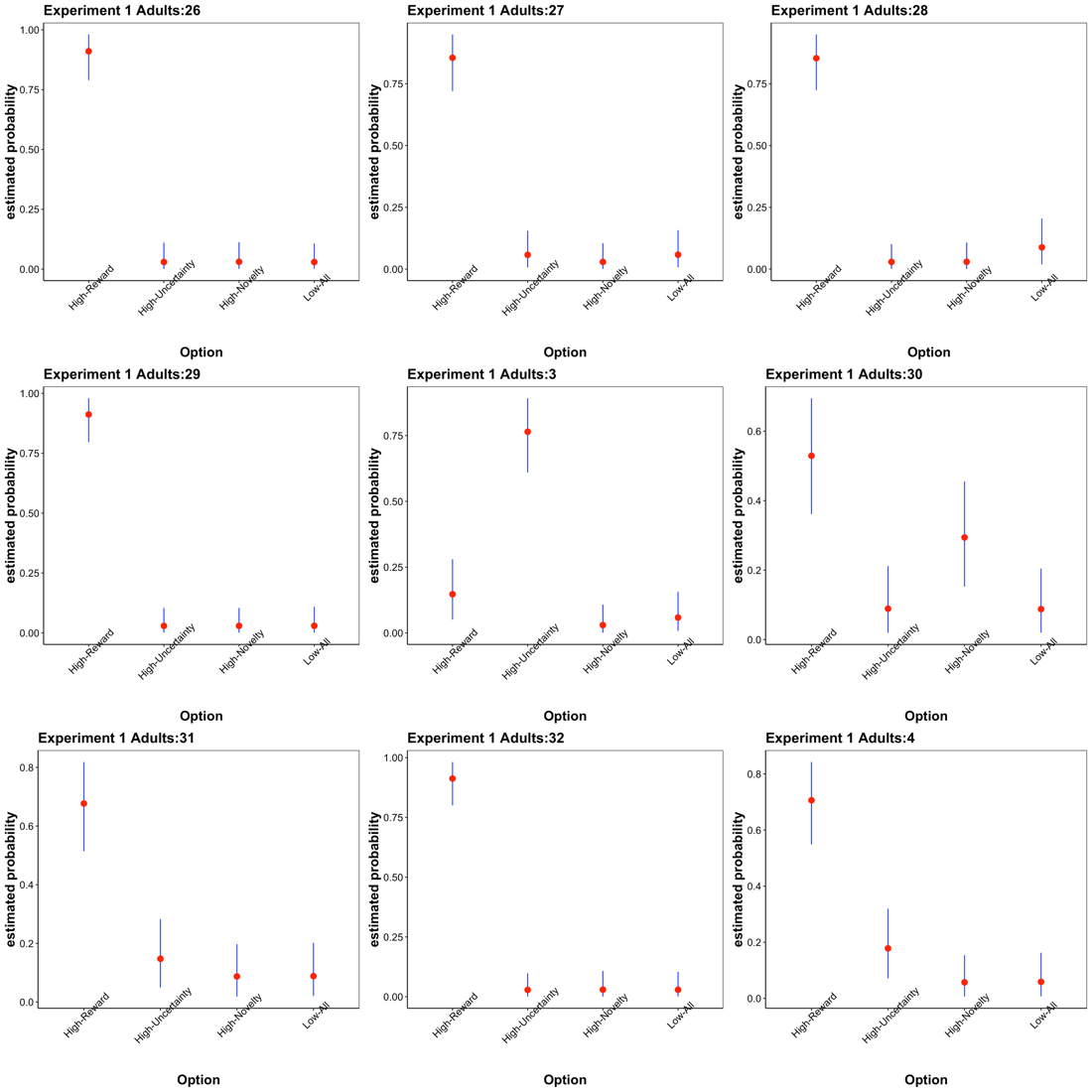

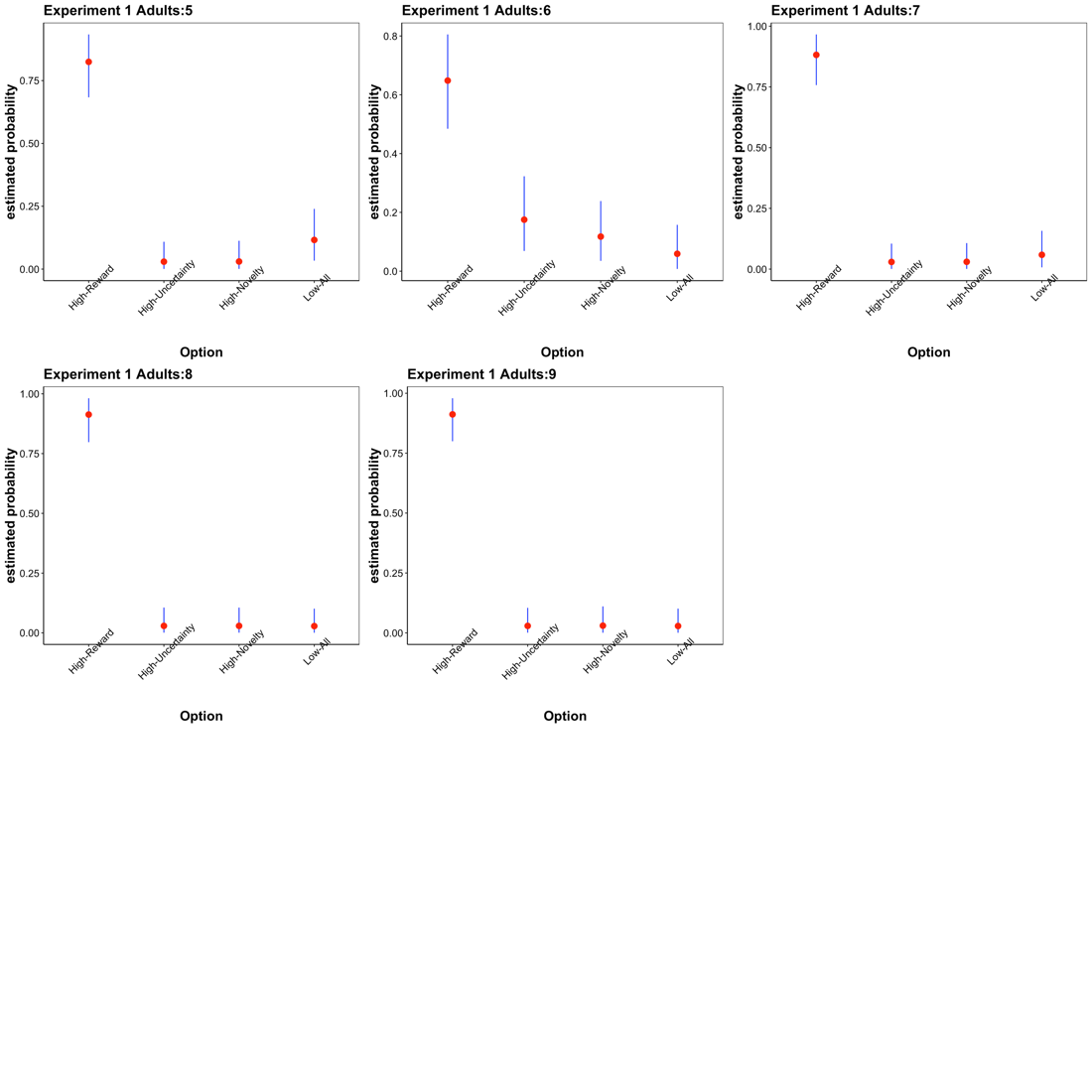


*Supplemental Figure 4*. Posterior Means and 95% credible intervals for the probabilities of choosing each option for adults in Experiment 1 testing.

**Win-stay Lose-shift Strategy**

The finding that both 4-year-olds and 5- to 6-year-olds predominately chose the High-Novelty option in the testing, despite maximizing rewards in the training, suggests that novelty may disrupt exploitation in early development. To further investigate whether the introduction of novelty indeed resulted in young children’s insensitivity to rewards, we examined and compared the use of win-stay lose-shift strategy across age groups. As the name implies, people employing the win-stay lose-shift strategy tend to repeat an option after they gained a high reward (win) while switching to a different option after they received little rewards (lose). The use of win-stay lose-shift strategy indicates that participants were sensitive to rewards and using some form of a reward-seeking strategy.

To examine whether participants followed a win-stay lose-shift strategy, we calculated the mean rewards gained on trials where participants stayed on the next trial versus trials where participants switched, for each age group separately. We then conducted paired *t*-test for each age group. The analysis was only conducted for the testing phase when rewards were hidden from the participants. If participants followed a win-stay lose-shift strategy, they would gain more rewards on trials where they chose to stay than on trials where they chose to switch. The results were shown in Supplemental Figure 5. Specifically, in Experiment 1, both adults and 5- to 6-year-olds gained significantly more rewards on trials they chose to stay than on trials they chose to switch, *p*s < 0.001. However, for 4-year-olds, there was no significant difference, *p* = 0.5226. The results suggested that adults and 5- to 6-year-olds were sensitive to rewards even though 5- to 6-year-olds showed a preference for the High-Novelty option. In contrast, 4-year-olds appeared to be driven solely by the novelty of the option. Furthermore, when analyzing only trials where 4-year-olds chose the High-Novelty option, no significant results were found either, *p* = 0.3051, further demonstrating that 4-year-olds were not sensitive to rewards when a novel option was present.


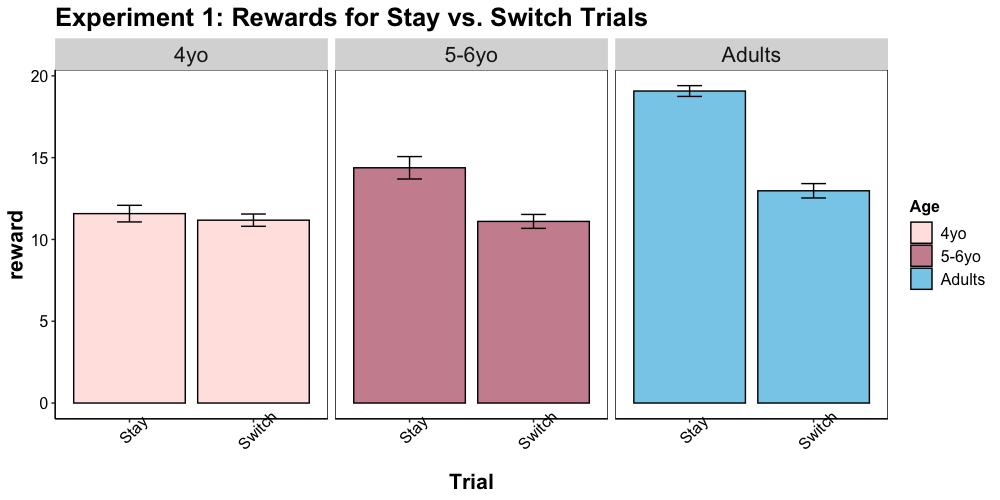


*Supplemental Figure 5*. Mean rewards gained on “stay” trials versus “switch” trials of 4-year-olds (left), 5- to 6-year-olds (middle), and adults (right) in Experiment 1. The error bars represent standard errors.

**Additional Analyses on Estimated Weights**

To investigate age differences in relative influences of factors contributing to participants’ decisions in the training and testing phases, we conducted one-way ANOVAs on best-fitting parameter values of different contributing factors. For training, we found significant age differences in the weight of reward, *F*(2,91) = 8.131, *p* < 0.001, $\eta_{p}^{2}$ = 0.15, objective uncertainty, *F*(2,91) = 3.146, *p* = 0.048, $\eta_{p}^{2}$ = 0.06, and choice lag, *F*(2,91) = 5.175, *p* < 0.01, $\eta_{p}^{2}$= 0.10. Particularly, the weight of reward was higher for adults ($M_{adults}$ = 0.85, ${SD}_{adults}$ = 0.22) than both 4-year-olds ($M_{4yo}$ = 0.54, ${SD}_{4yo}$ = 0.36) and 5- to 6-year-olds ($M_{5-6yo}$= 0.63, ${SD}_{5-6yo}$= 0.33), *p*s *<*  0.05, with no significant differences between two child groups, *p =*  0.50. In addition, the weight of uncertainty was higher for 4-year-old ($M_{4yo}$ = 0.08, ${SD}_{4yo}$ = 0.15) than adults ($M_{adults}$ = 0.02, ${SD}_{adults}$ = 0.04), *p =*  0.047, but there was no significant difference 4-year-olds and 5- to 6-year-olds ($M_{5-6yo}$= 0.06, ${SD}_{5-6yo}$= 0.09) or between 5- to 6-year-olds and adults, *p*s > 0.17. Moreover, the weight of choice lag was higher for 4-year-olds ($M_{4yo}$ = 0.38, ${SD}_{4yo}$ = 0.34) than adults ($M_{adults}$ = 0.13, ${SD}_{adults}$ = 0.22), *p <* 0.01. However, no significant differences were found between 5- to 6-year-olds ($M_{5-6yo}$= 0.31, ${SD}_{5-6yo}$= 0.35) and 4-year-ods, or between 5-to-6-year-olds and adults, *p*s > 0.07.

For testing, we found a significant age difference in value, *F*(2,91) = 23.8, *p* < 0.001, $\eta_{p}^{2}$ = 0.34, novelty, *F*(2,91) = 10.92, *p* < 0.001, $\eta_{p}^{2}$= 0.19, and objective uncertainty, *F*(2,91) = 6.514, *p* < 0.01, $\eta_{p}^{2}$ = 0.13, with no significant age effect on the weight of choice lag, *p* = 0.304. The weight of value was higher for adults ($M_{adults}$ = 0.67, ${SD}_{adults}$ = 0.25), than 5- to 6-year-olds ($M_{5-6yo}$= 0.32, ${SD}_{5-6yo}$= 0.30) and 4-year-olds ($M_{4yo}$ = 0.23, ${SD}_{4yo}$ = 0.25), *p*s *<*  0.001, but the two child age groups did not differ significantly from each other, *p* = 0.423. More importantly, the weight of perceptual novelty was higher for 4-year-olds ($M_{4yo}$ = 0.32, ${SD}_{4yo}$ = 0.27) and 5- to 6-year-olds ($M_{5-6yo}$= 0.24, ${SD}_{5-6yo}$= 0.25) than adults ($M_{adults}$ = 0.06, ${SD}_{adults}$ = 0.10), *p*s *<*  0.01, but the two child age groups did not differ significantly from each other, *p* = 0.341. Similarly, the weight of objective uncertainty was higher for 4-year-olds ($M_{4yo}$ = 0.24, ${SD}_{4yo}$ = 0.17) and 5- to 6-year-olds ($M_{5-6yo}$= 0.19, ${SD}_{5-6yo}$= 0.17) than adults ($M_{adults}$ = 0.10, ${SD}_{adults}$ = 0.11), *p*s *<*  0.05, but the two child age groups did not differ significantly from each other, *p* = 0.509.

**Random Exploration**

To examine age differences in the randomness of their exploration, we analyzed age differences in $\log(\lambda)$ (log-transformed $\lambda$) using the Kruskal-Wallis test because $\lambda$ had extreme values (i.e., outliers) and is not normally distributed. The results revealed a significant age difference in $\lambda$, *H*(2) = 28.797, *p* < 0.0001. The post-hoc analysis revealed that both 4-year-olds ($M_{4yo}$ = 1.21, ${SD}_{4yo}$ = 0.64) and 5- to 6-year-olds ($M_{5-6yo}$= 2.04, ${SD}_{5-6yo}$= 2.46) explored more randomly than adults ($M_{adults}$ = 2.63, ${SD}_{adults}$ = 1.59), *p*s *<*  0.01, but the two child age groups did not significantly differ from each other, *p* = 0.152. The results indicated that children’s choice was more stochastic compared to adults. However, given that only two children in 4-year-old group had a $\lambda$ < 1, and the majority of all age groups had a $\lambda$ > 3, we tend to not over-interpret the age differences as all participants were very deterministic.

**Experiment 2**

**Bayesian Multinomial Test on Choice Proportion**

In Experiment 2, a Bayesian multinomial logistic regression model was fit to each individual participant’s choices during both training and test to estimate the posterior distributions of the probabilities for each option during the two phases. The model fitting results for each participant are shown in Supplemental Figure 6 (4-year-olds at training), Supplemental Figure 7 (5- to 6-year-olds at training), Supplemental Figure 8 (adults at training), Supplemental Figure 9 (4-year-olds at test), Supplement Figure 10 (5- to 6-year-olds at test), and Supplemental Figure 11 (adults at test). The results showed that during training, three different decision patterns could be identified in 4-year-olds, with 14 out of 30 4-year-olds predominately choosing the High-Novelty Option over the other options, 7 out of 30 4-year-olds predominately choosing the High-Reward Option, and remaining showing no clear preferences. Unlike 4-year-olds, most 5- to 6-year-olds (22 out of 30) and adults (31 out of 34) clearly prioritized the High-Reward option over all the other options. However, at test, while all adults and most 5-year-olds (17 out of 30) were highly reward-based, only 4 out of 30 children showed clear preference toward the High-Novelty Option (yet they were not reward-driven either). The results aligned with what we reported in the main text, providing converging evidence that perceptual novelty drives children’s choice decisions via a bottom-up process.

**
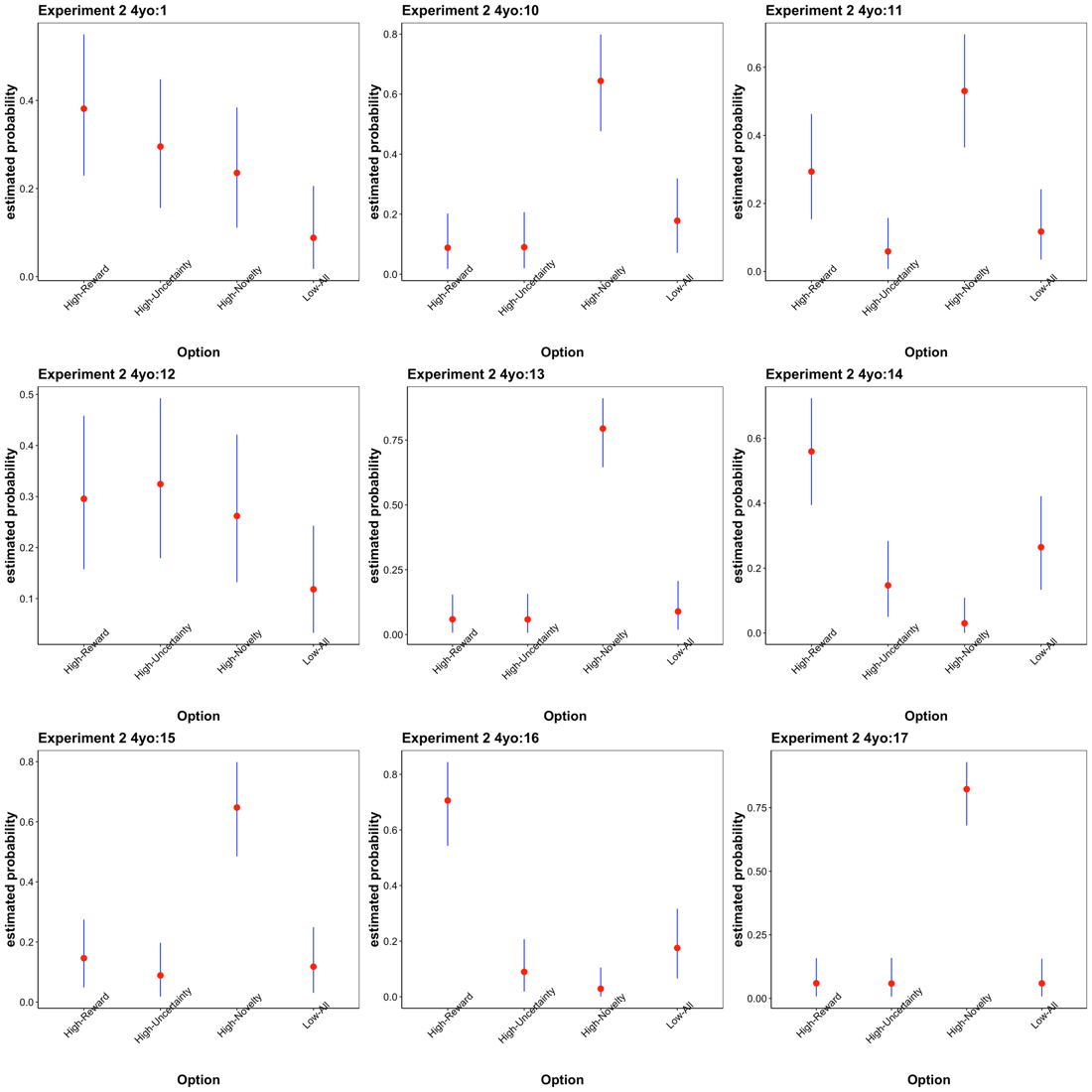

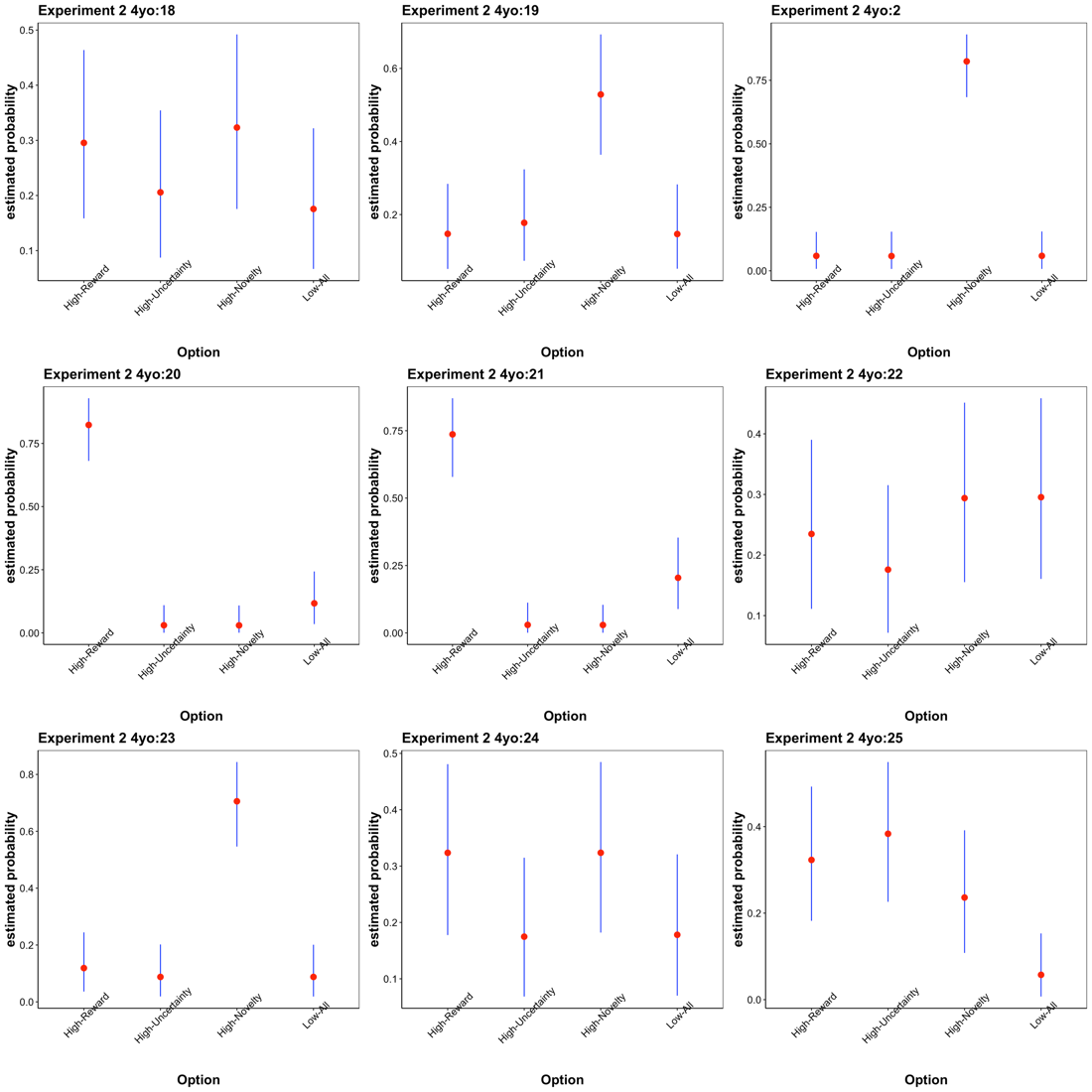

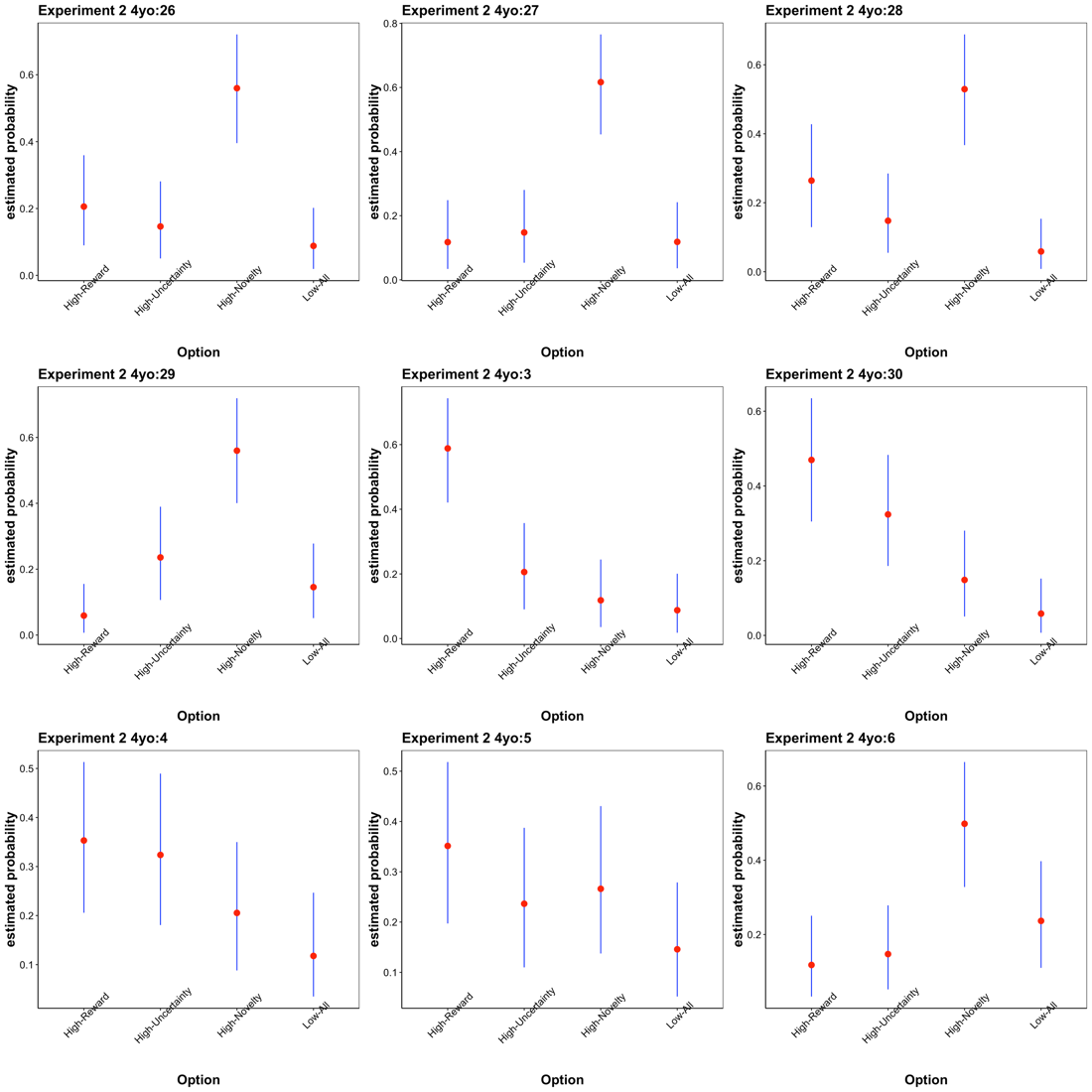

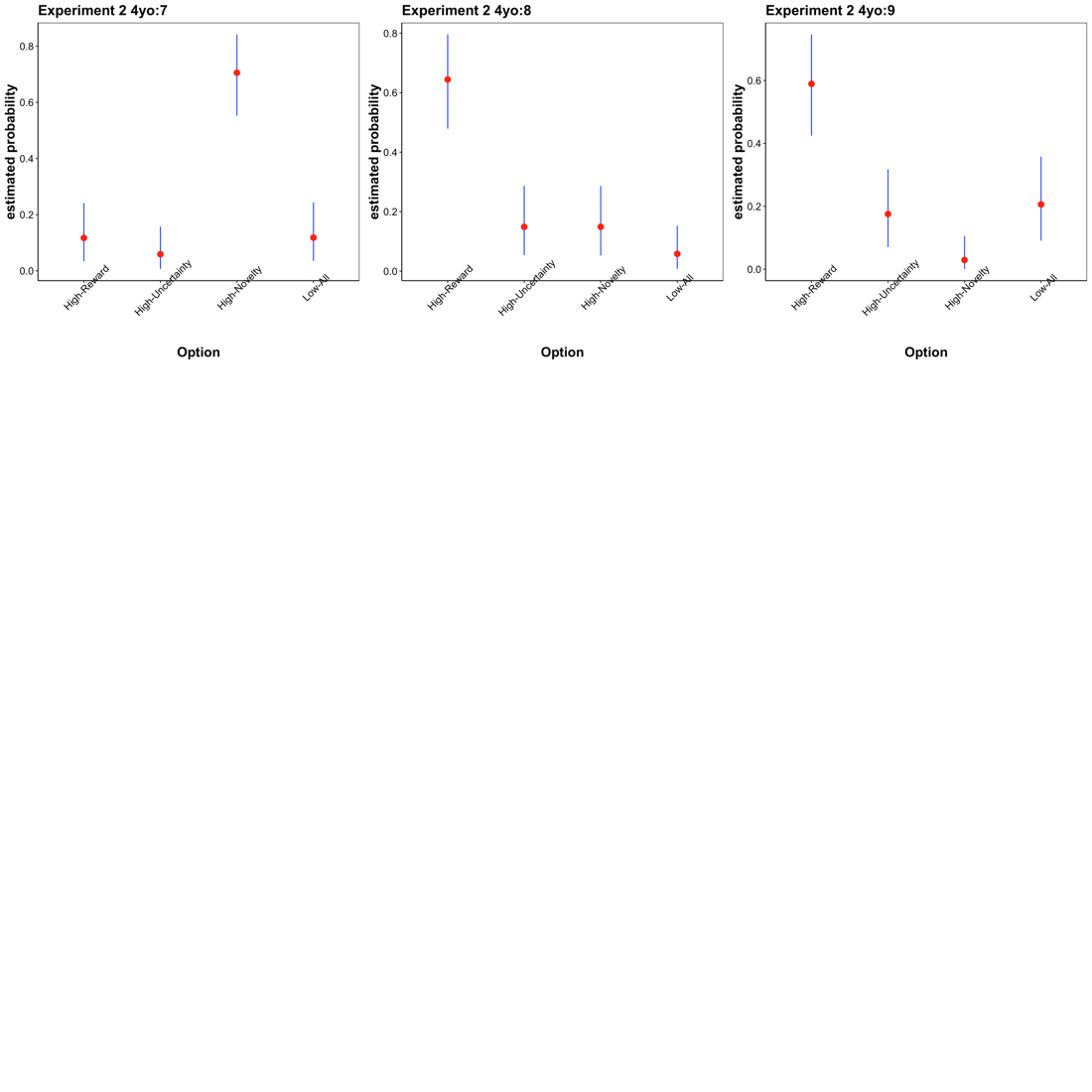
**

*Supplemental Figure 6*. Posterior Means and 95% credible intervals for the probabilities of choosing each option for 4-year-olds in Experiment 2 training.


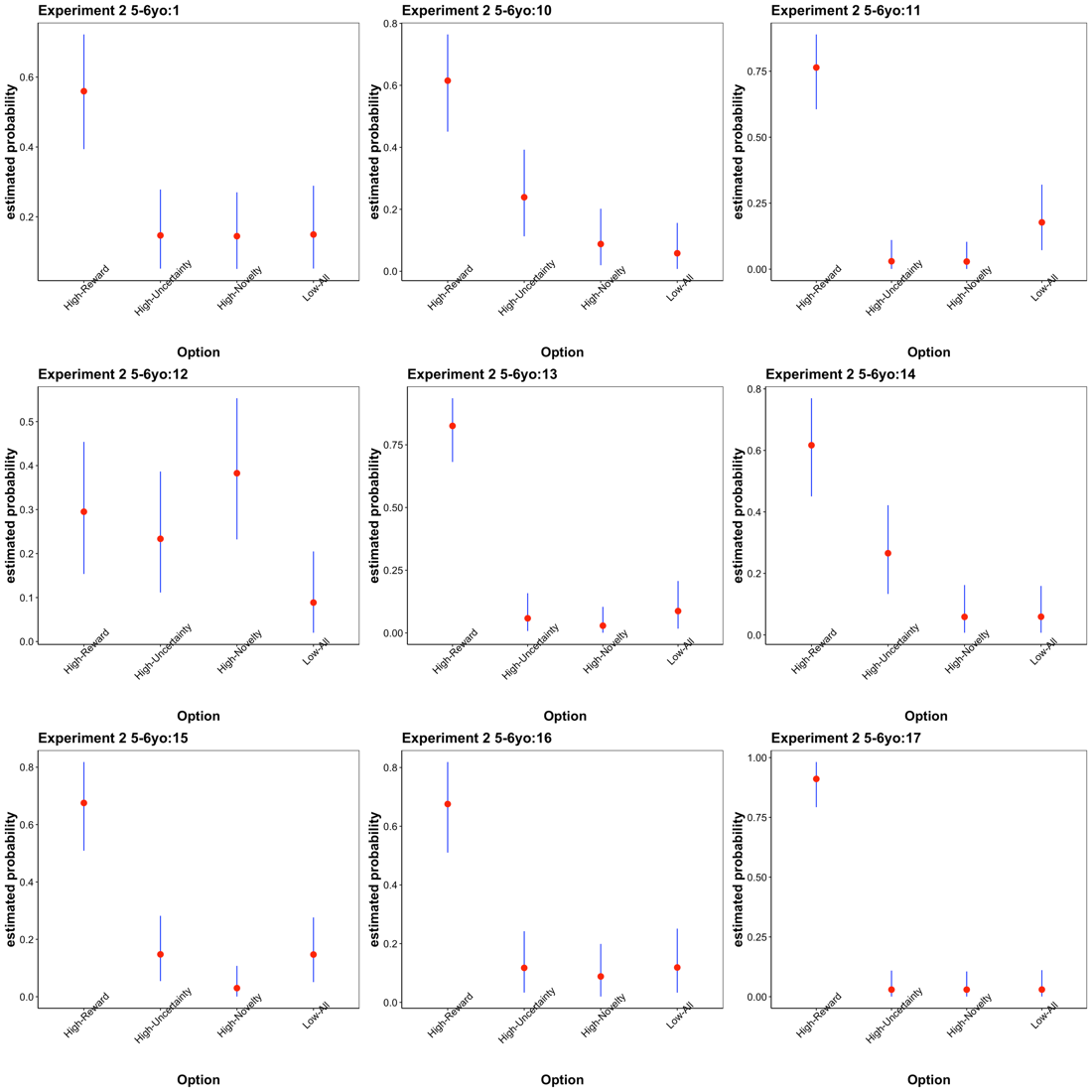

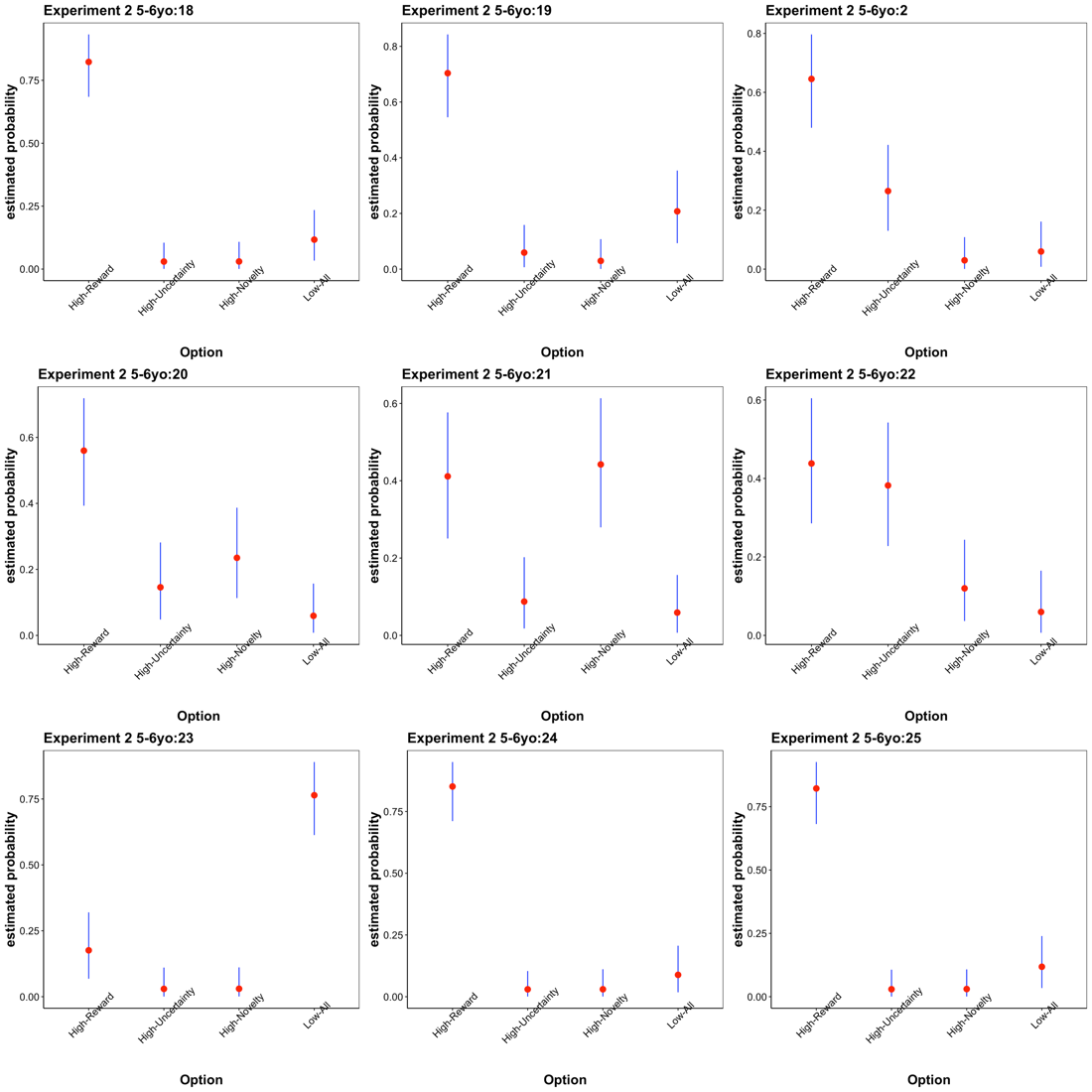

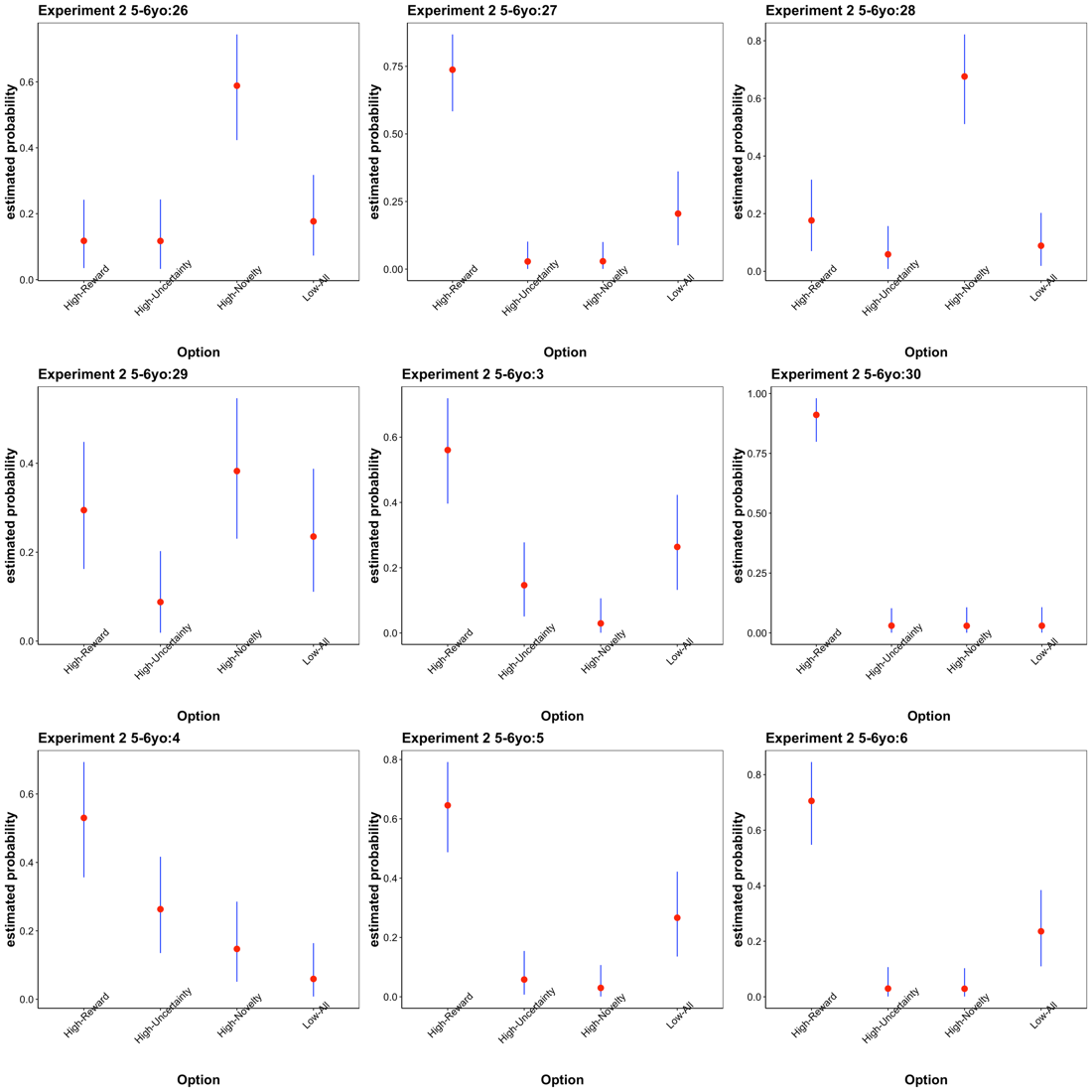

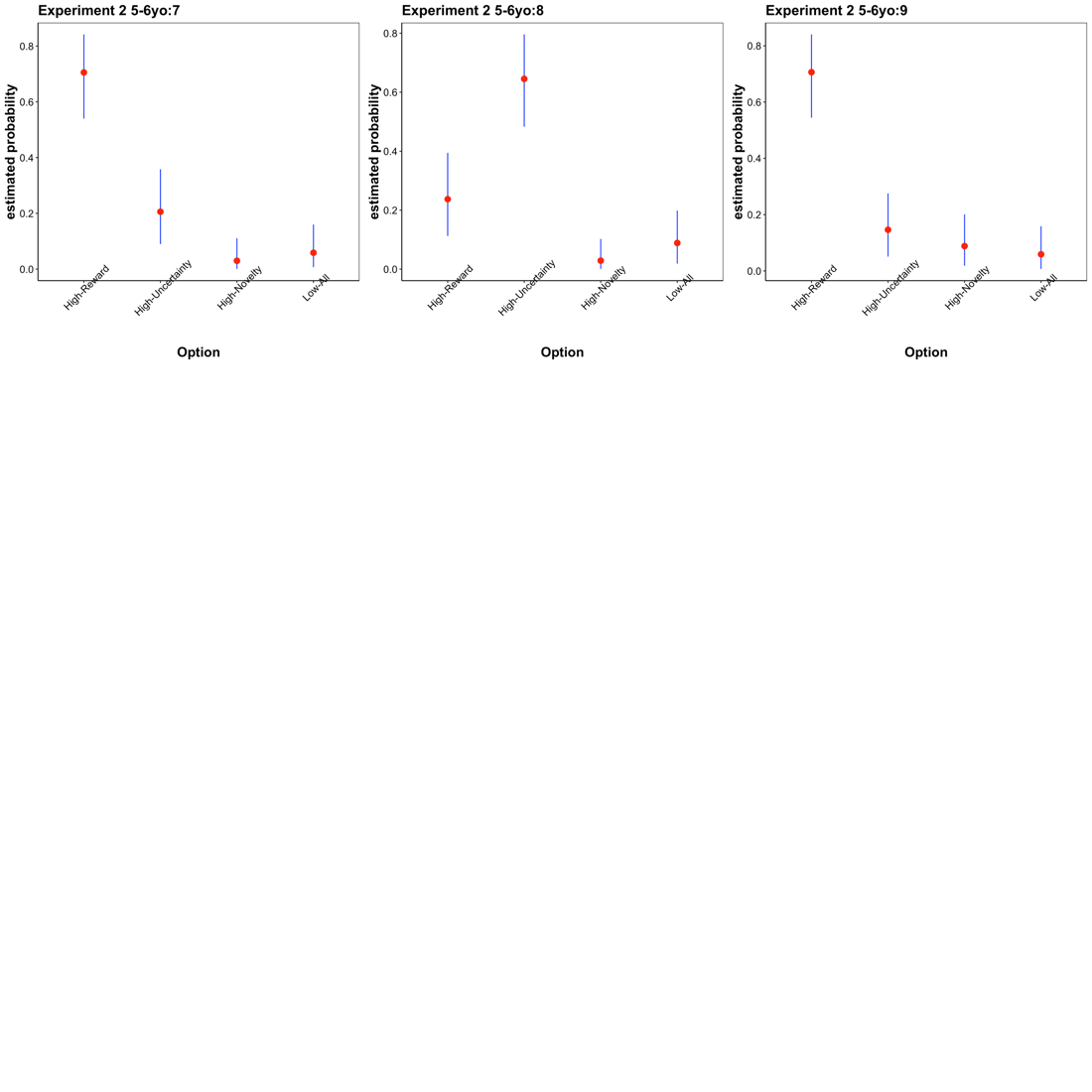


*Supplemental Figure 7*. Posterior Means and 95% credible intervals for the probabilities of choosing each option for 5- to 6-year-olds in Experiment 2 training.


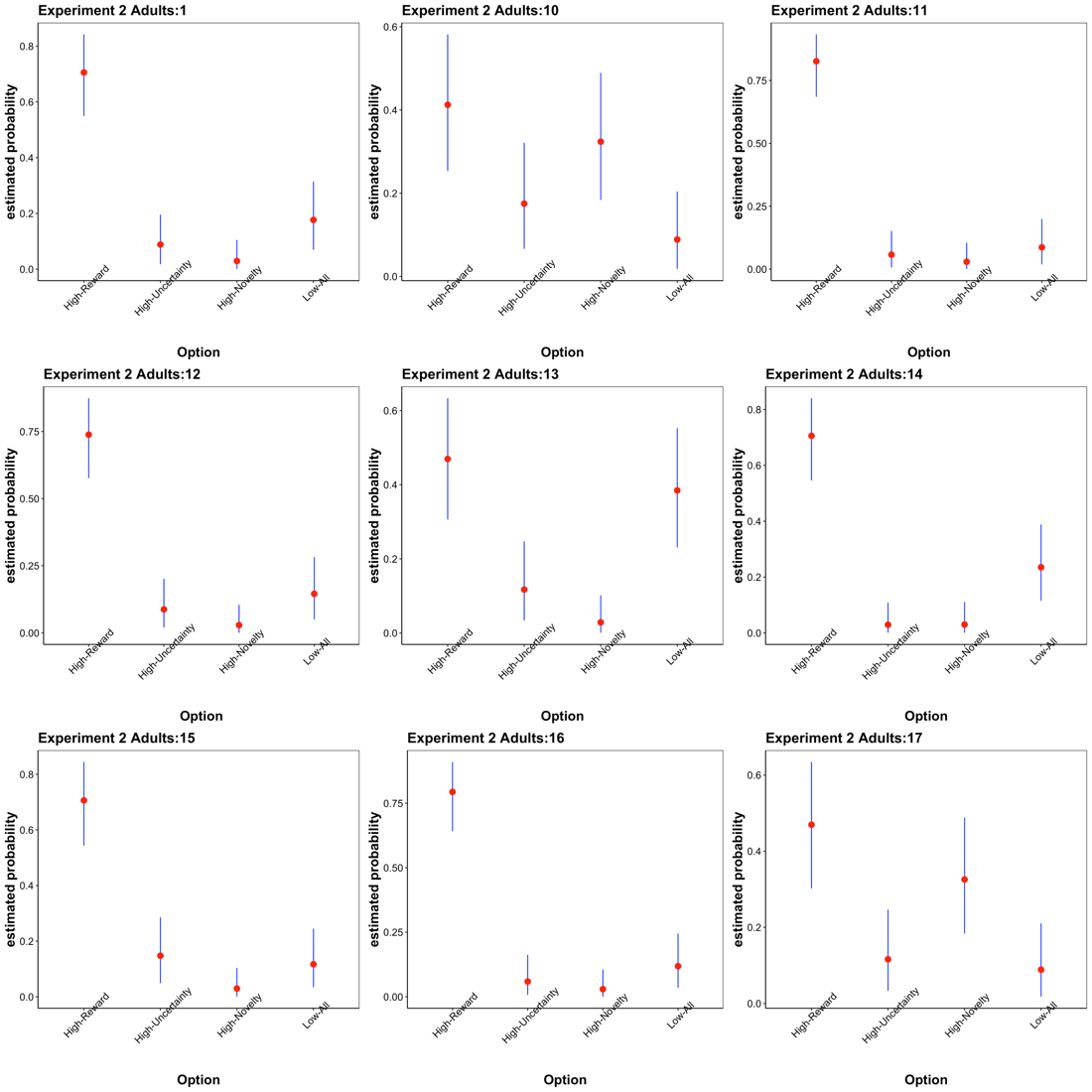

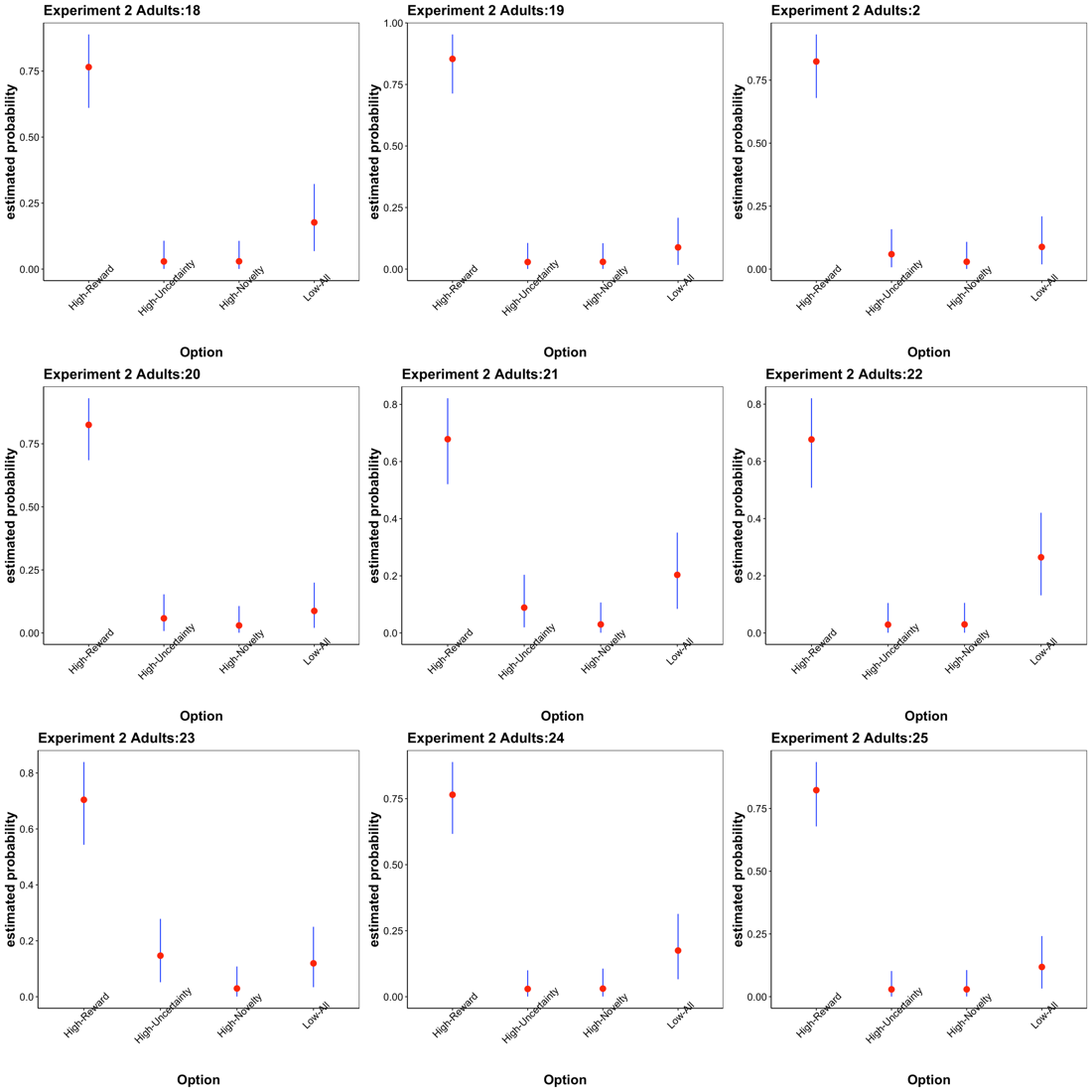

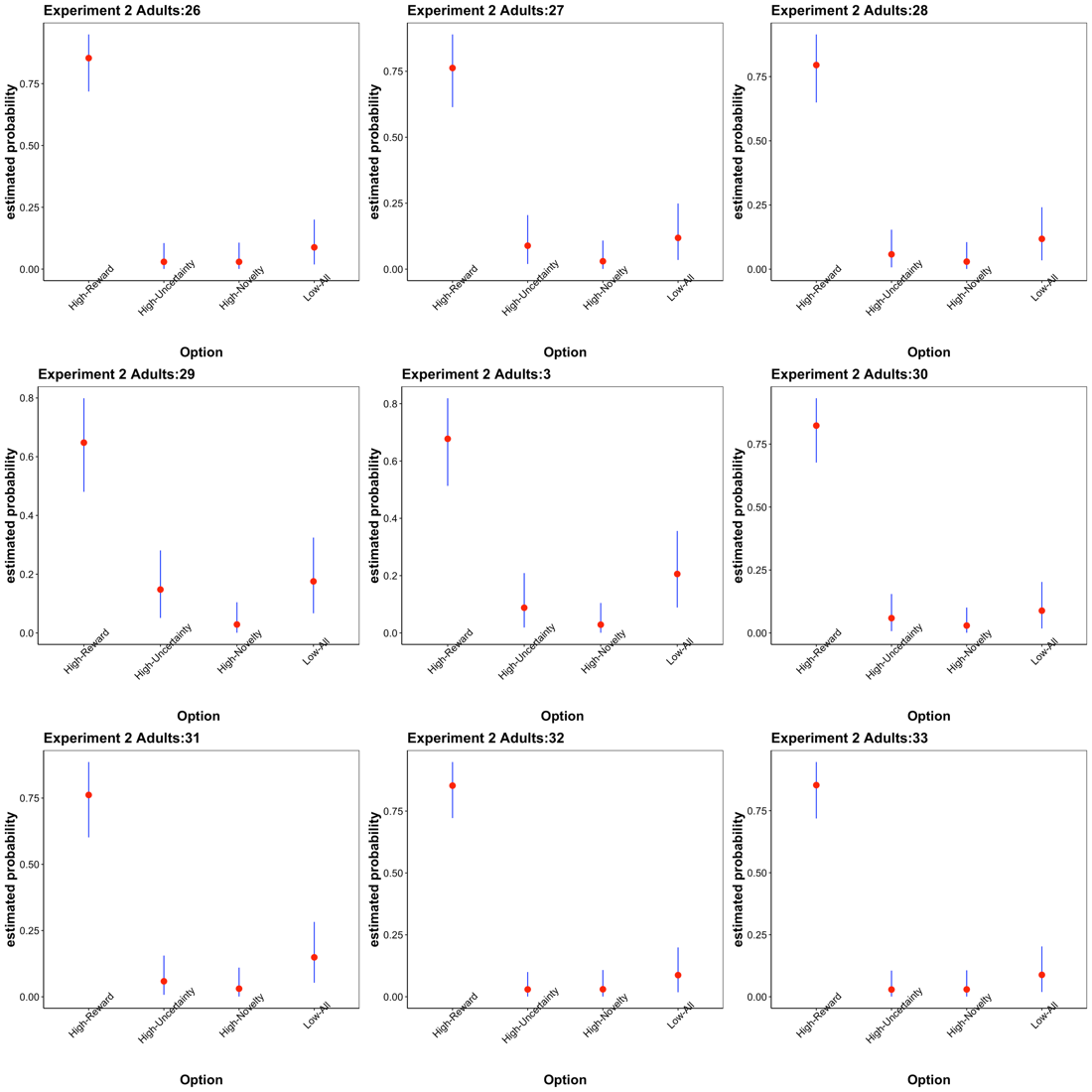

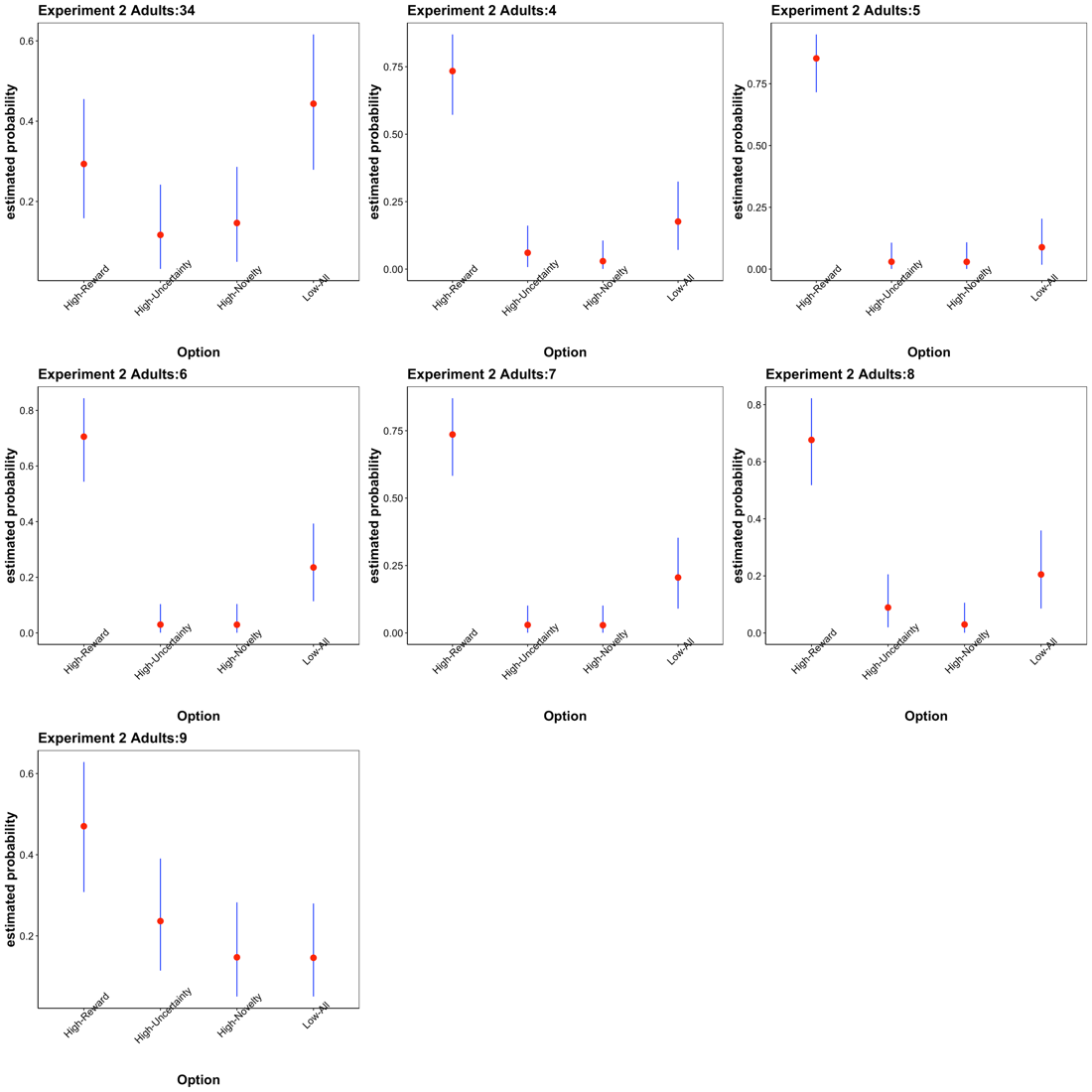


*Supplemental Figure 8*. Posterior Means and 95% credible intervals for the probabilities of choosing each option for adults in Experiment 2 training.

**
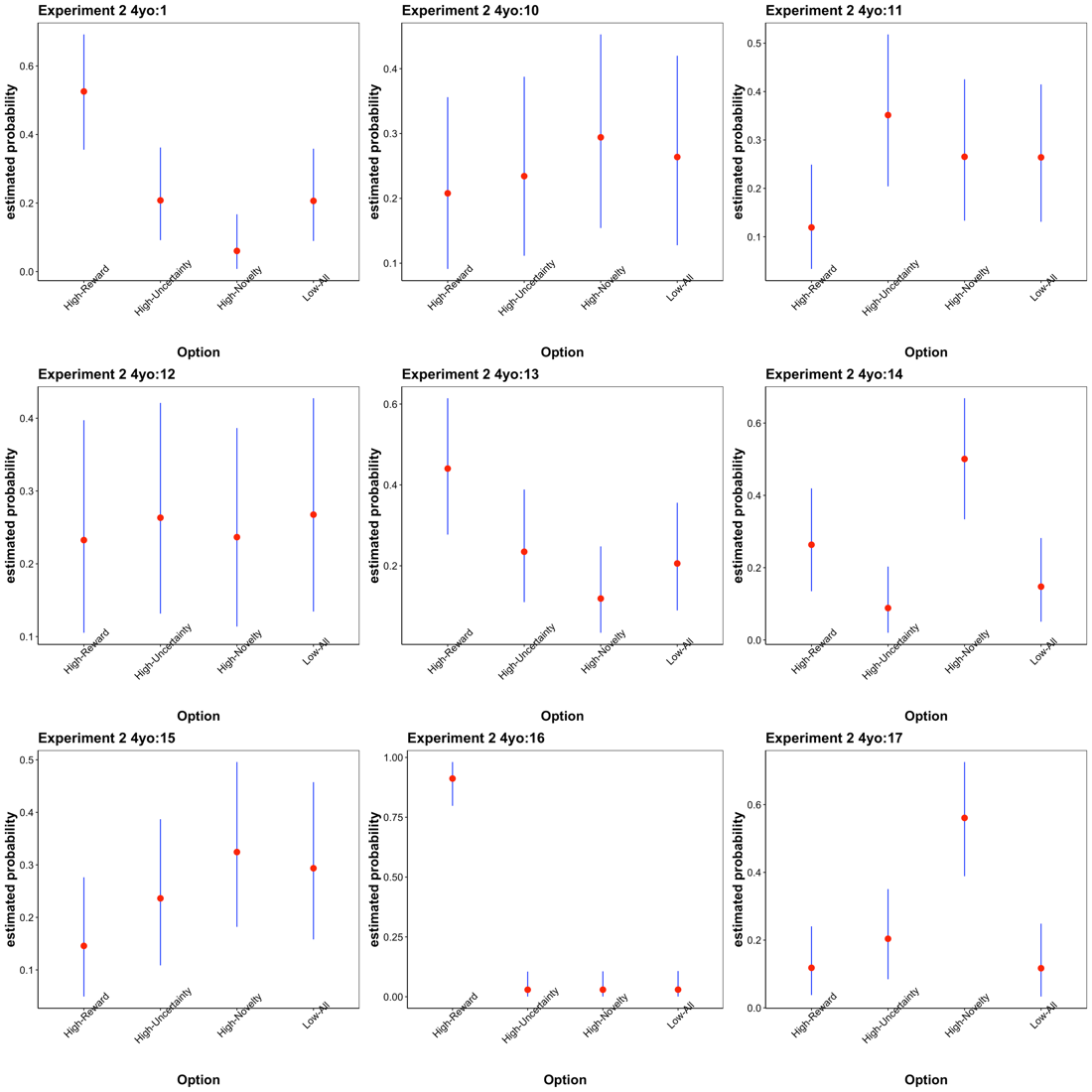

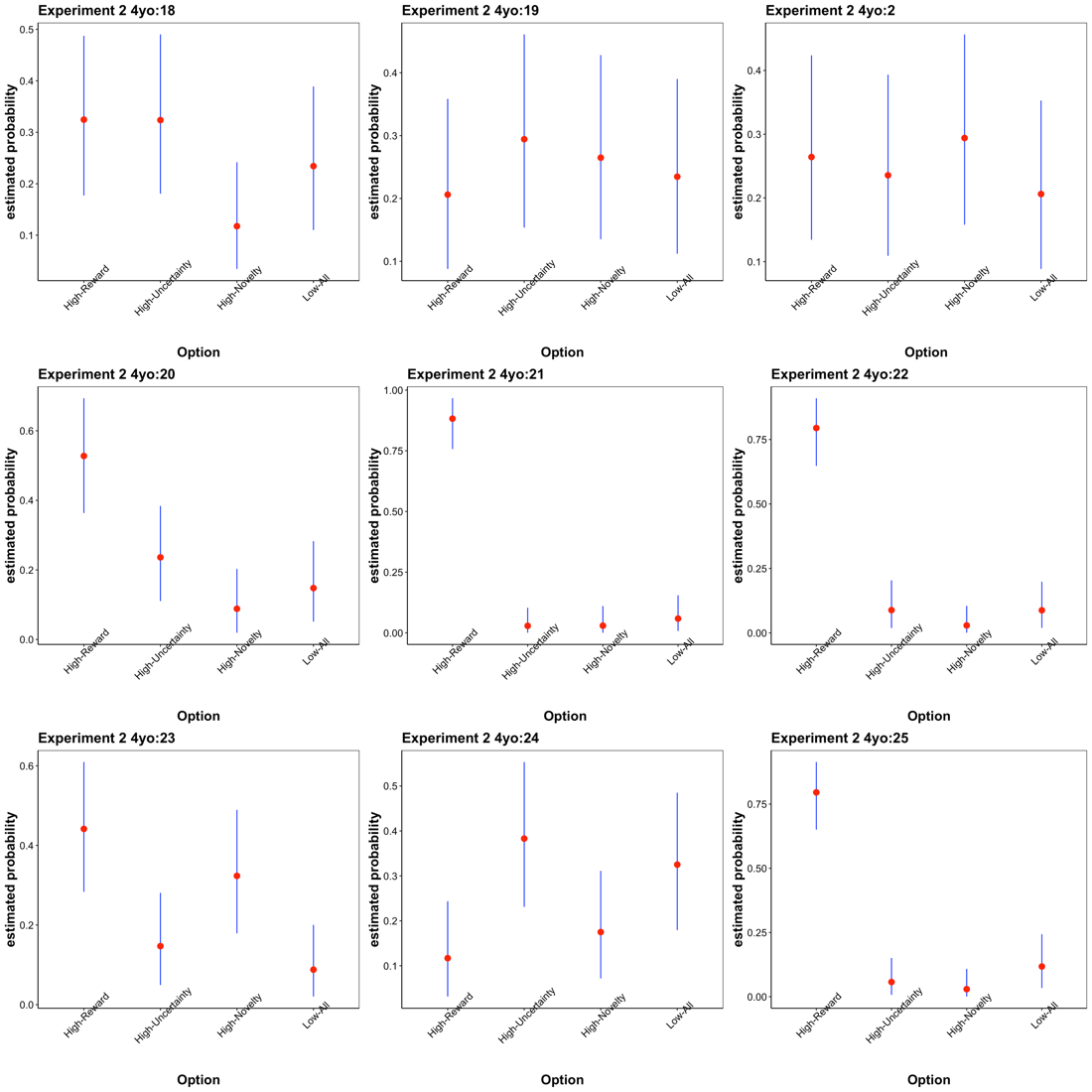

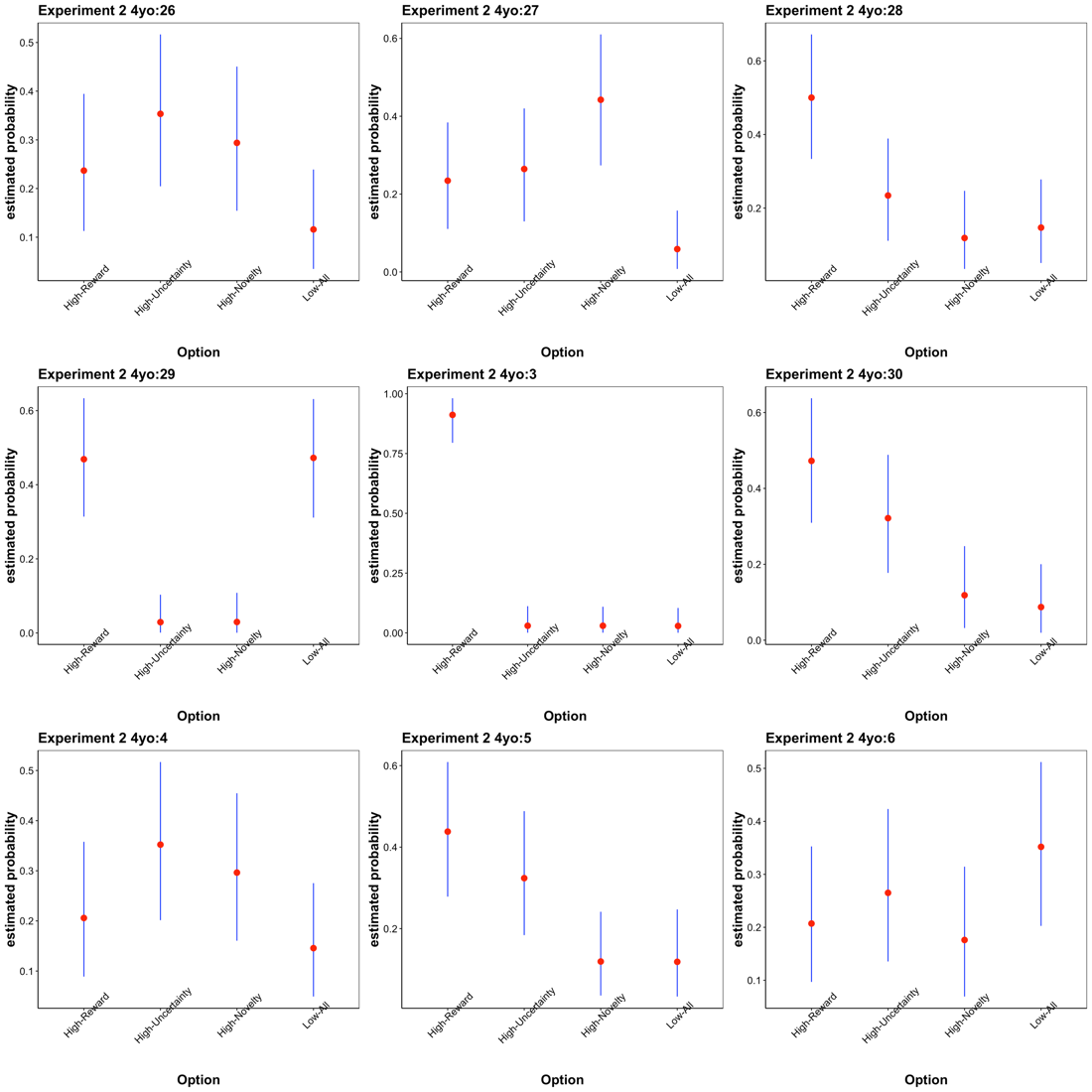

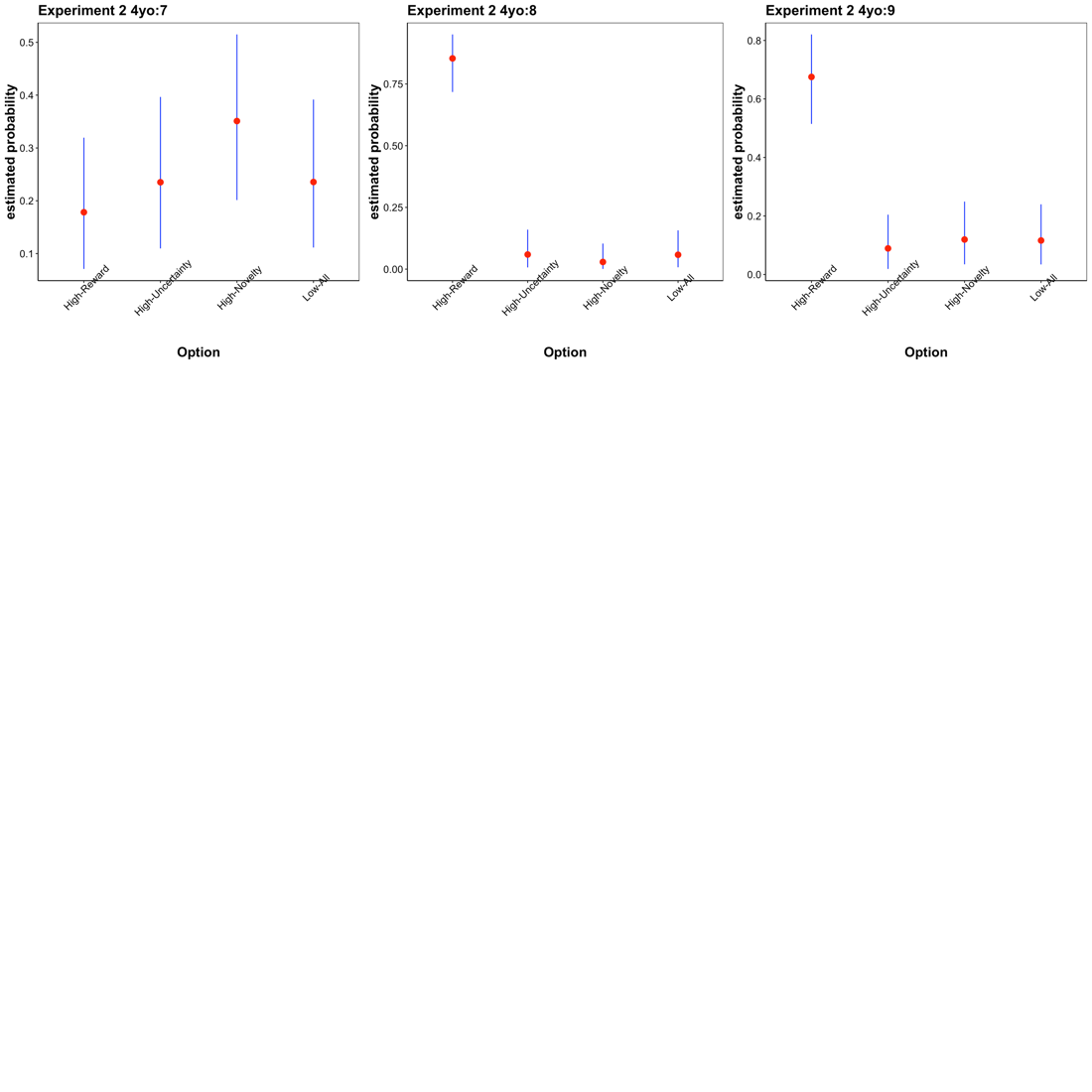
**

*Supplemental Figure 9*. Posterior Means and 95% credible intervals for the probabilities of choosing each option for 4-year-olds in Experiment 2 testing.


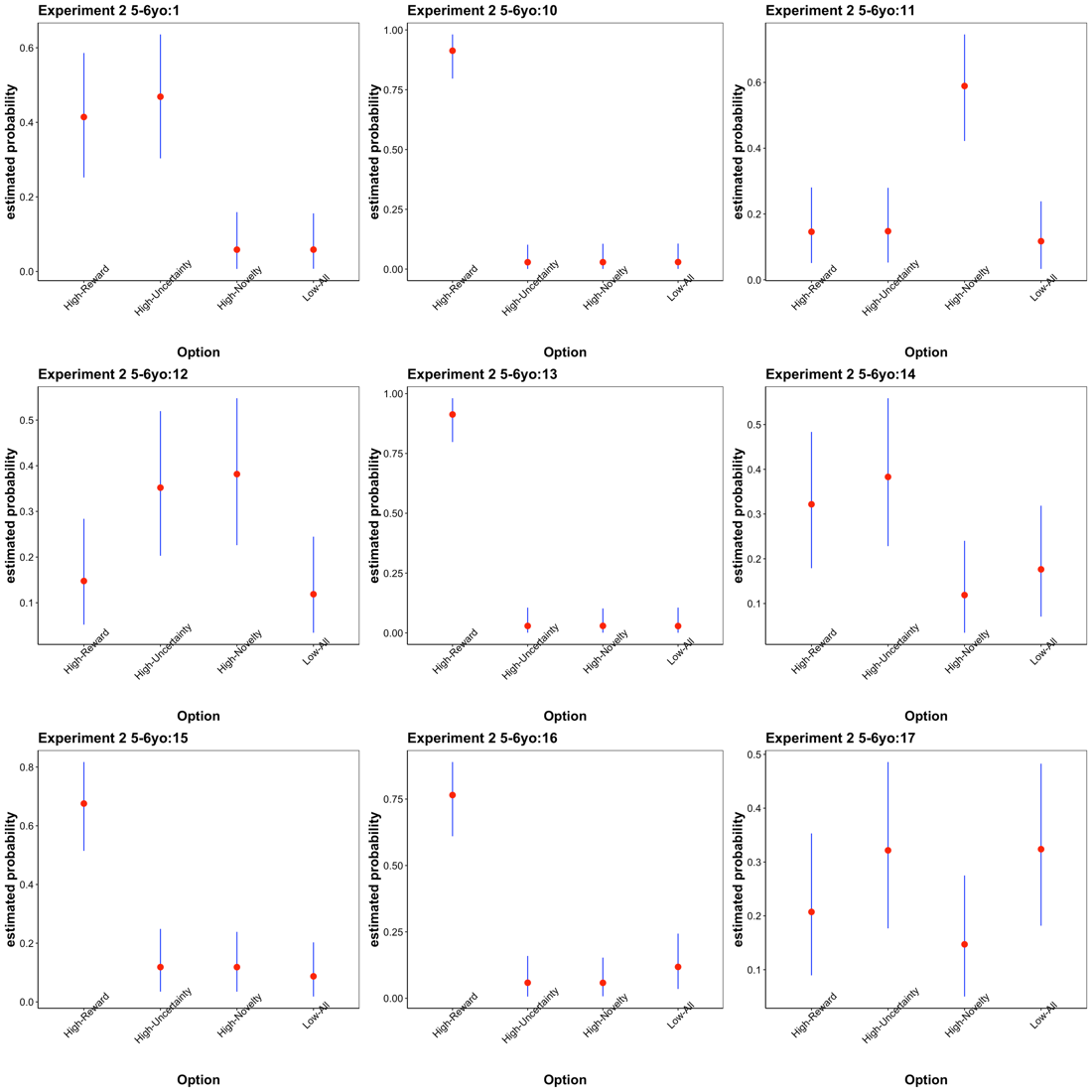

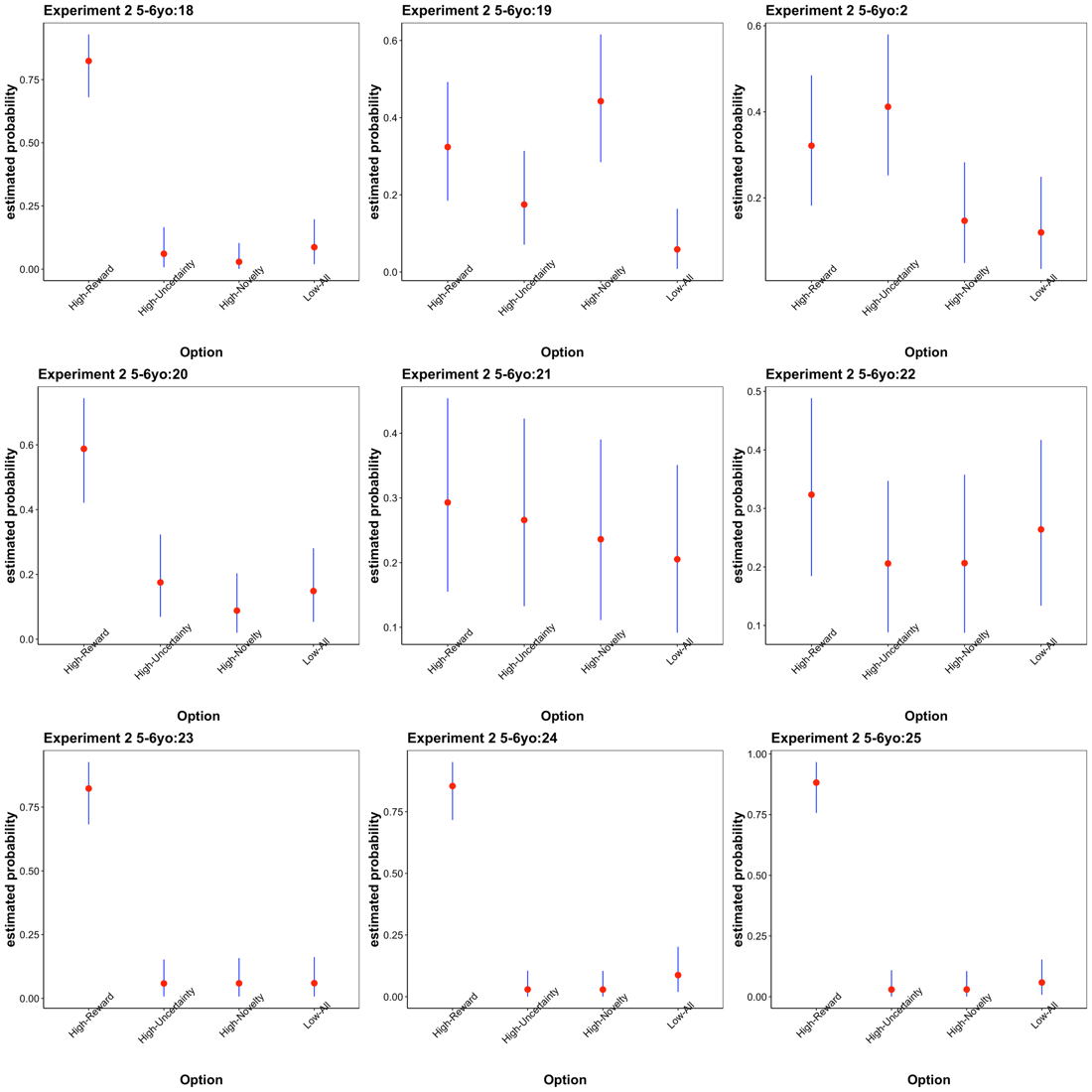

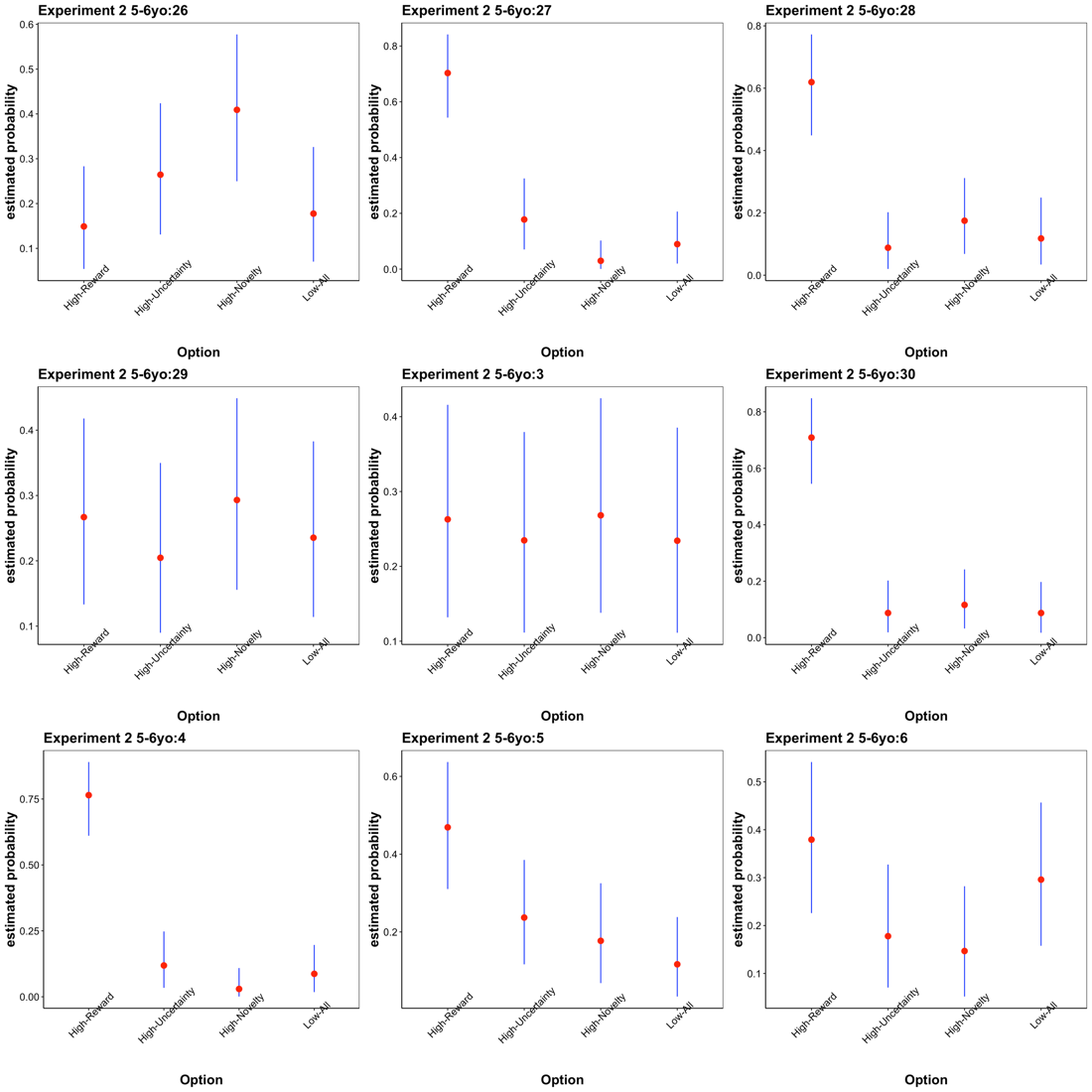

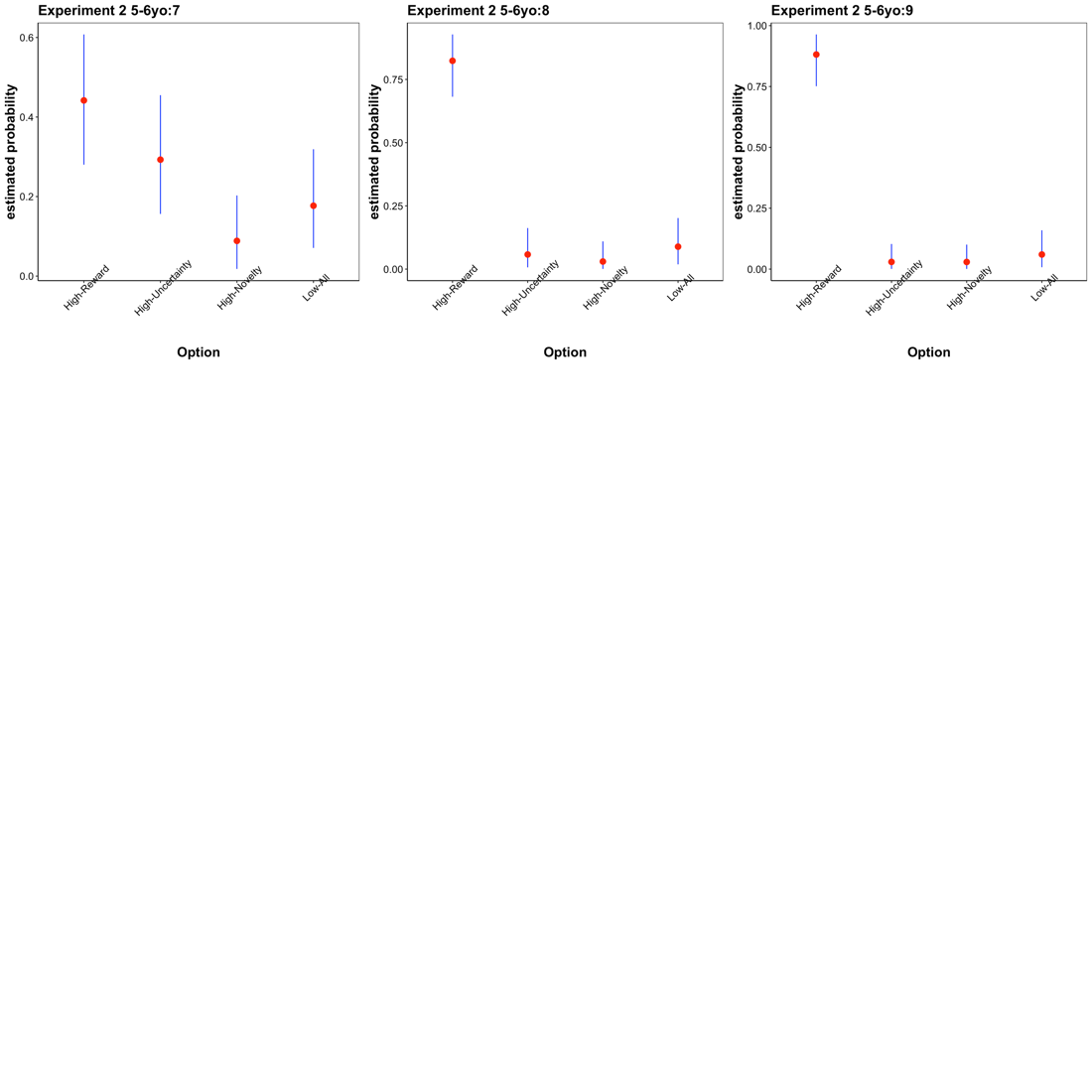


*Supplemental Figure 10*. Posterior Means and 95% credible intervals for the probabilities of choosing each option for 5- to 6-year-olds in Experiment 2 testing.


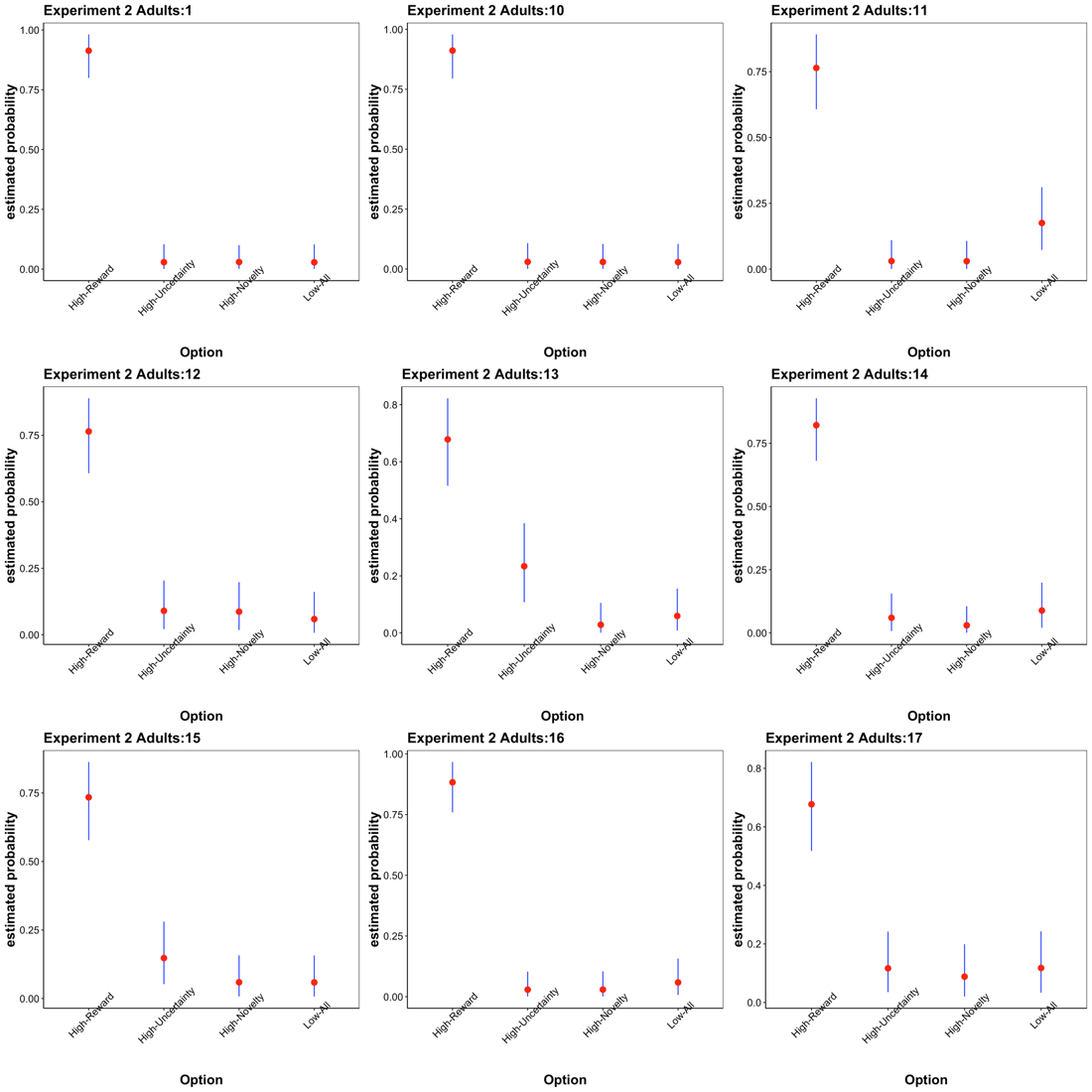

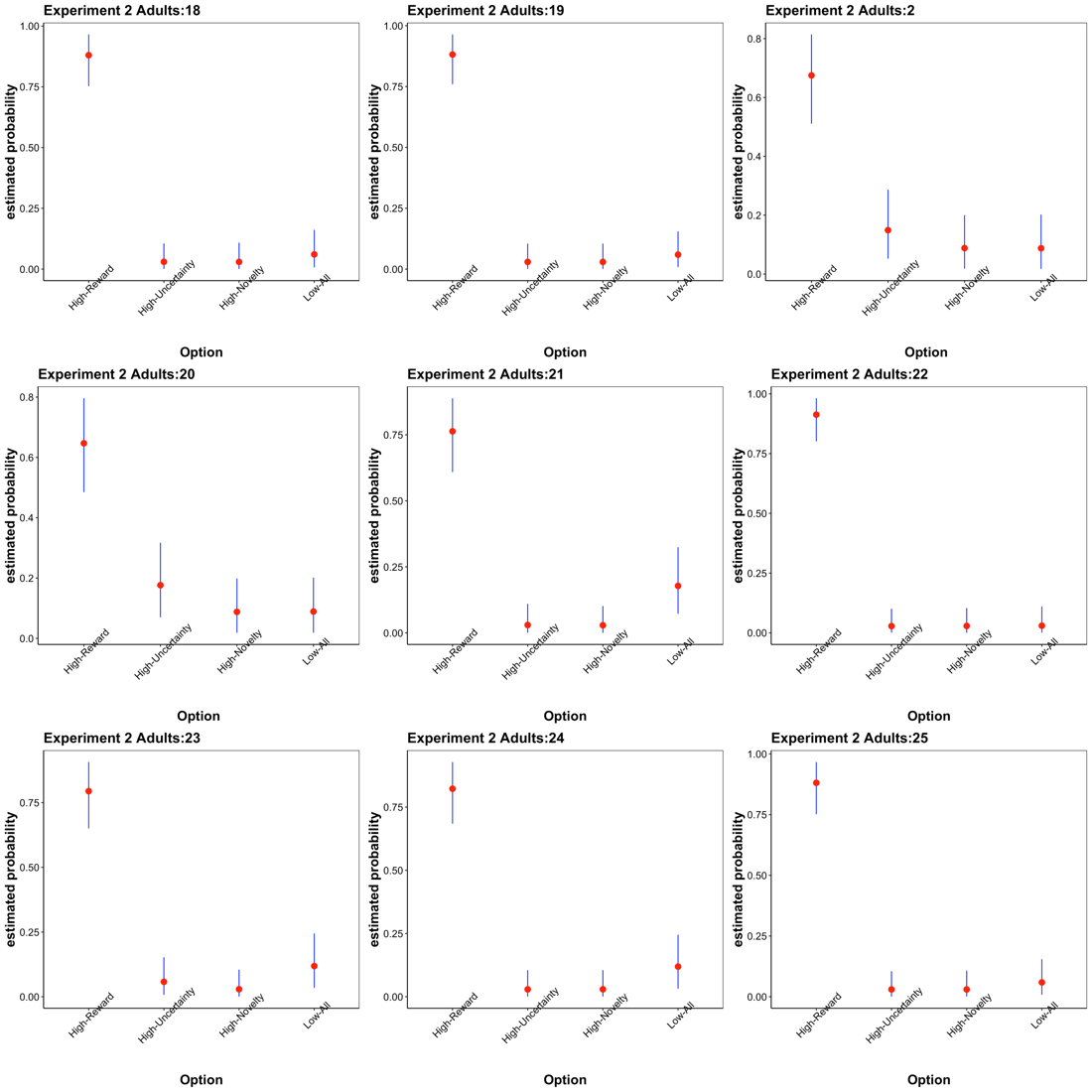

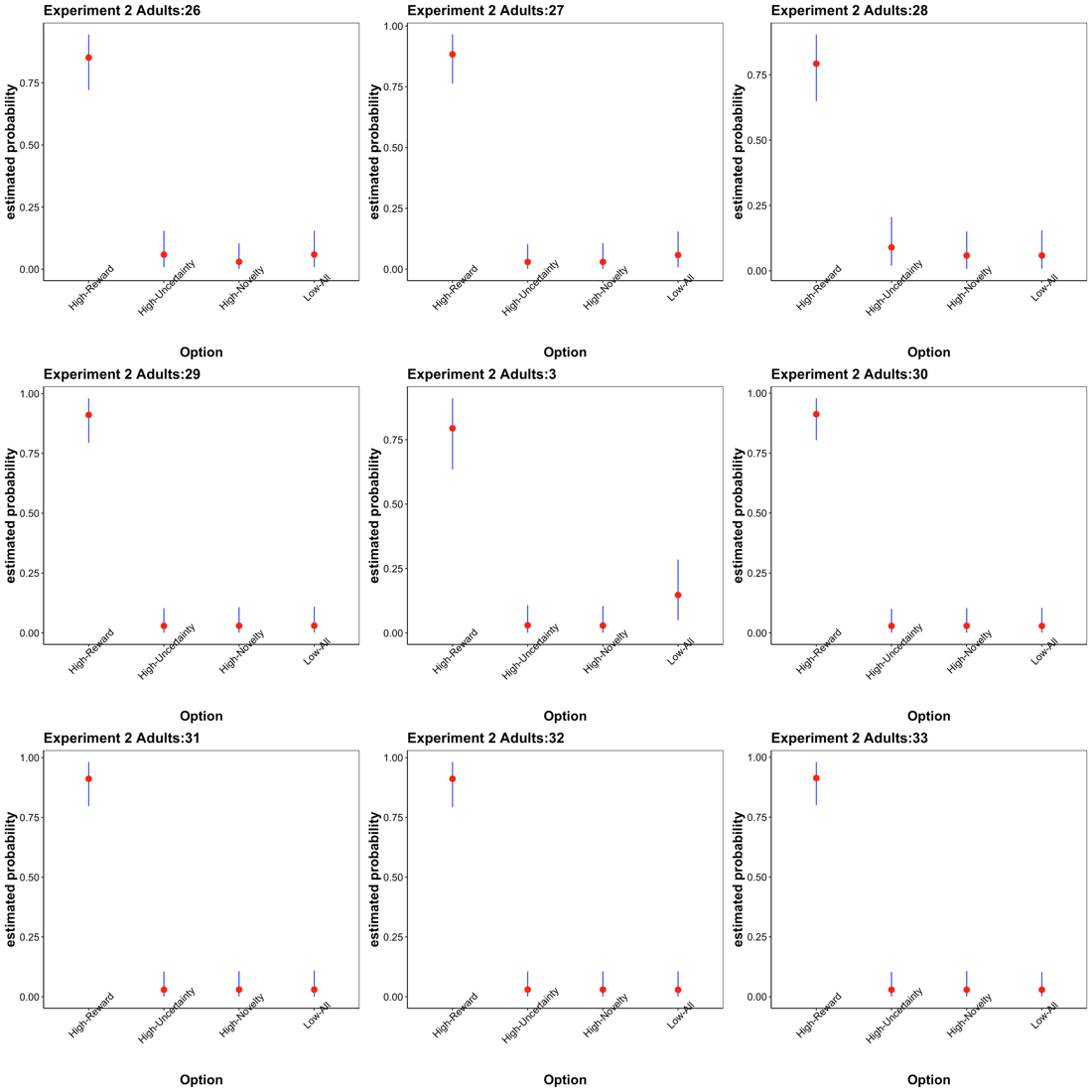

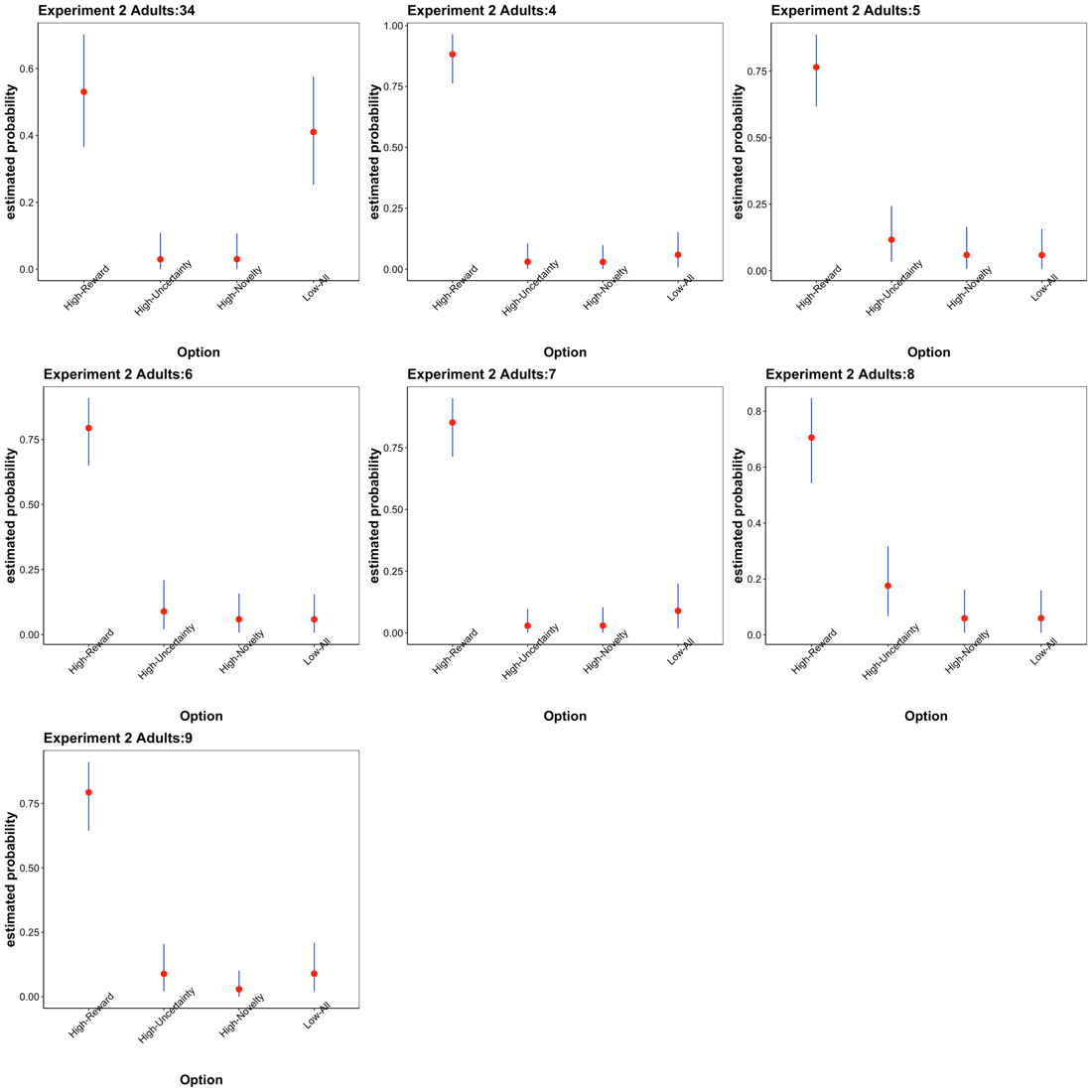


*Supplemental Figure 11*. Posterior Means and 95% credible intervals for the probabilities of choosing each option for adults in Experiment 2 testing.

**Win-stay Lose-shift Strategy**

Same as in Experiment 1, we examined the win-stay lose-shift strategy in the testing across age groups. The results from a paired *t*-test showed that participants of all age groups gained significantly more rewards on trials they chose to stay than trials they chose to switch, *p*s < 0.01. The results indicated that when the perceptual novelty of the High-Novelty option was no longer observable, 4-year-olds became more sensitive to rewards and adopted a reward-seeking strategy. The finding aligns with our other results and provided additional evidence for the bottom-up attentional effects of perceptual novelty.


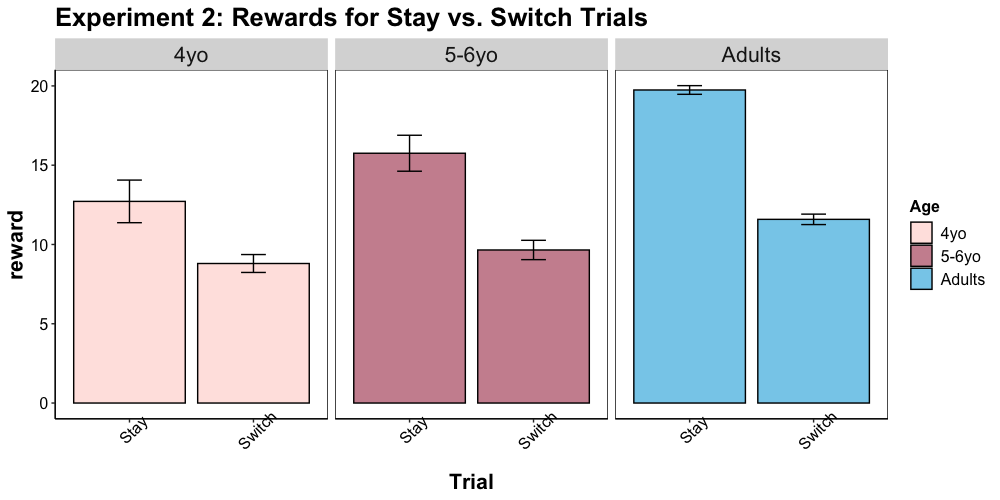


*Supplemental Figure 12*. Mean rewards gained on “stay” trials versus “switch” trials of 4-year-olds (left), 5- to 6-year-olds (middle), and adults (right) in Experiment 2. The error bars represent standard errors.

**Four-year-olds’ Value-Based Decisions Across Experiments**

An interesting and striking behavioral difference was noted in 4-year-olds’ performance during the training of the two experiments. While 4-year-olds were primarily driven by the reward value and maximized the reward gain as instructed in Experiment 1, their choice decisions were predominated by the High-Novelty option when it was introduced in Experiment 2. Such differences indicated that perceptual novelty could disrupt young children’s exploitive behaviors. To provide statistical evidence for this claim, we conducted a chi-square test of independence on aggregated data of 4-year-olds to examine whether there was a significant difference in their preferences towards the High-Reward option during the training phase across Experiments. The results yielded a significant difference between the two experiments, ${}^{2}(1, 1800)$ = 116.51, *p* < 0.001. Specifically, 4-year-olds were more likely to choose the High-Reward option in the training phase of Experiment 1 (N = 523, out of 900) compared to Experiment 2 (N = 294, out of 900). The results provided further evidence that novelty could disrupt young children’s reward-based exploitation.

**The Interaction between Memory and Choice Proportion**

Although 4-year-olds, as a group, demonstrated above-chance accuracy on the memory question for the High-Reward option, half of them failed to identify the High-Reward option at the end of the experiment. This suggests a potential relationship between young children’s choice proportions and their memory for the High-Reward option. Specifically, their choice decisions during the training phase (which presumably reflected their attention allocation) might influence their learning and memory for each option. In turn, their learning of each option likely influenced their performance in the testing. To investigate this interaction between 4-year-olds’ choice proportions and their memory for the High-Reward option, we conducted additional analyses. The results are presented below.

We divided 4-year-olds in Experiment 2 into two groups: the “High-Reward Learner” group (those who correctly answered the High-Reward question, N = 15) and the “High-Reward Non-Learner” group (those who did not, N = 15) and analyzed their data separately. Specifically, we conducted Repeated-measures ANOVAs on the two groups’ choice proportions during the training and testing phases separately, with option type as the within-subject factor.

For the training phase, the results revealed a significant effect of option type for both the “High-Reward Learner” group, *F*(3, 42) = 9.483, *p* < 0.001, $\eta_{p}^{2}$ = 0.40, and the “High-Reward Non-Learner” group, *F*(3, 42) = 28.74, *p* < 0.001, $\eta_{p}^{2}$ = 0.67. As shown in the figure below, 4-year-olds who correctly answered the High-Reward question chose the High-Reward option significantly more than other options, *p*s < 0.05. On the contrary, 4-year-olds who failed to answer the High-Reward question correctly chose the High-Novelty option significantly more than other options, *p*s < 0.001.

For the testing phase, the results yielded a significant effect of option type for the “High-Reward Learner” group, *F*(3, 42) = 24.16, *p* < 0.001, $\eta_{p}^{2}$ = 0.63, but a non-significant result for the “High-Reward Non-Learner” group. Same as in Experiment 1, 4-year-olds who correctly answered the High-Reward question primarily chose the High-Reward option over all other options. However, for those who did not answer the question correctly, there were no significant differences in their choice proportions during the testing phase.

Taken together, the results suggested different behavioral patterns between 4-year-olds who answered the memory question for the High-Reward option correctly and those who did not. Specifically, young children who answered the question correctly were able to maximize rewards during both training and testing phases. In addition, and more importantly, the results indicated that the high proportion of choosing the High-Novelty option in 4-year-olds during the training phase was primarily driven by young children who failed to answer the memory question for the High-Reward option correctly. These findings are not surprising, as young children who predominantly chose the High-Novelty option might have focused exclusively on it during training. As a result, the lack of attention to other options impeded their learning of these options.

**Additional Analyses on Estimated Weights**

Same as in Experiment 1, we first conducted One-way ANOVAs on the weights of all contributing factors to examine age differences in relative influences of factors contributing to participants’ choice decisions in the training and testing phases separately, and all post-hoc pairwise comparison results are annotated in Figure 6 in the main text. Specifically, we found significant age differences for all contributing factors in the training phase: reward value, *F*(2,91) = 16.11, *p* < 0.001, $\eta_{p}^{2}$= 0.26; objective uncertainty, *F*(2,91) = 3.683, *p* = 0.029, $\eta_{p}^{2}$= 0.07; perceptual novelty, *F*(2,91) = 6.77, *p* < 0.01, $\eta_{p}^{2}$= 0.13; and choice lag, *F*(2,91) = 3.562, *p* = 0.0324, $\eta_{p}^{2}$ = 0.07. Specifically, the weight of reward was higher for adults ($M_{adults}$ = 0.68, ${SD}_{adults}$ = 0.23) and 5- to 6-year-olds ($M_{5-6yo}$= 0.62, ${SD}_{5-6yo}$= 0.32) than 4-year-olds ($M_{4yo}$ = 0.31, ${SD}_{4yo}$ = 0.27), *p*s *<*  0.001, with no significant differences between adults and 5- to 6-year-olds, *p =* 0.64. In addition, the weight of uncertainty was higher for 4-year-old ($M_{4yo}$ = 0.051, ${SD}_{4yo}$ = 0.07) than adults ($M_{adults}$ = 0.015, ${SD}_{adults}$ = 0.04), *p =*  0.048, but there was no significant difference between 4-year-olds and 5- to 6-year-olds ($M_{5-6yo}$= 0.048, ${SD}_{5-6yo}$= 0.07) or between 5- to 6-year-olds and adults, *p*s > 0.06. Moreover, the weight of choice lag was higher for 4-year-olds ($M_{4yo}$ = 0.17, ${SD}_{4yo}$ = 0.23) than adults ($M_{adults}$ = 0.05, ${SD}_{adults}$ = 0.19), *p =* 0.0428. However, no significant differences were found between 5- to 6-year-olds ($M_{5-6yo}$= 0.06, ${SD}_{5-6yo}$= 0.11) and 4-year-ods, or between 5-to-6-year-olds and adults, *p*s > 0.08. Most importantly, the weight of perceptual novelty was higher for 4-year-olds ($M_{4yo}$ = 0.47, ${SD}_{4yo}$ = 0.31) than both 5- to 6-year-olds ($M_{5-6yo}$= 0.27, ${SD}_{5-6yo}$= 0.28) and adults ($M_{adults}$ = 0.25, ${SD}_{adults}$ = 0.19), *p*s *<*  0.01, with no significant differences between adults and 5- to 6-year-olds, *p >*  0.95.

For contributing factors in testing, we found a significant main effect of age for the weight of value, *F*(2,91) = 9.81, *p* < 0.001, $\eta_{p}^{2}$ = 0.18, the weight of anticipated perceptual novelty, *F*(2,91) = 6.385, *p* < 0.01, $\eta_{p}^{2}$ = 0.12, and the weight of choice lag, *F*(2,91) = 3.122, *p* = 0.0488, $\eta_{p}^{2}$= 0.06. No significant age effect was found for the weight of objective uncertainty, *p* = 0.485. Particularly, same as in the training phase, the weight of reward was higher for adults ($M_{adults}$ = 0.68, ${SD}_{adults}$ = 0.24) and 5- to 6-year-olds ($M_{5-6yo}$= 0.54, ${SD}_{5-6yo}$= 0.33) than 4-year-olds ($M_{4yo}$ = 0.35, ${SD}_{4yo}$ = 0.33, *p*s *<*  0.05, with no significant differences between adults and 5- to 6-year-olds, *p =*  0.19. Additionally, the weight of choice lag was marginally significantly higher for 4-year-olds ($M_{4yo}$ = 0.36, ${SD}_{4yo}$ = 0.32) than adults ($M_{adults}$ = 0.21, ${SD}_{adults}$ = 0.19), *p =* 0.052. However, no significant differences were found between 5- to 6-year-olds ($M_{5-6yo}$= 0.23, ${SD}_{5-6yo}$= 0.27) and 4-year-olds, or between 5-to-6-year-olds and adults, *p*s > 0.14. Furthermore, the weight of perceptual novelty was higher for 4-year-olds ($M_{4yo}$ = 0.19, ${SD}_{4yo}$ = 0.22) than adults ($M_{adults}$ = 0.05, ${SD}_{adults}$ = 0.10), *p <* 0.01, but there was no significant difference between 4-year-olds and 5- to 6-year-olds ($M_{5-6yo}$= 0.14, ${SD}_{5-6yo}$= 0.16) or between 5- to 6-year-olds and adults, *p*s > 0.06.

Critically, same as for behavioral data, to examine differential impacts of observable vs. anticipated perceptual novelty on participants’ choice decisions, we conducted Mixed ANOVA on the weight of perceptual novelty, with age being a between-subject variable, and phase or type of perceptual novelty (phase 1: observable vs. phase 2: anticipated) being a within-subject variable. The results revealed a significant main effect of age, *F*(2,91) = 10.159, *p* < 0.001, $\eta_{p}^{2}$ = 0.18, and a significant main effect of phase, *F*(1,91) = 48.548, *p* < 0.001, $\eta_{p}^{2}$= 0.35. The interaction did not reach significance, *p* = 0.127. The results demonstrated an overall important role of perceptual novelty in participants’ novelty-based decisions across all age groups.

Furthermore, given the same number of options in the training and testing phase, Experiment 2 provided an opportunity to examine whether choice lag captured participants’ epistemic uncertainty beyond pure anti-preservation switching behaviors. Specifically, given that the only difference between training and testing was the reduced information of all options in testing, if participants had a greater weight on the choice lag in testing than training, the increase could only result from their increased epistemic uncertainty about these options.

To explore this possibility, we conducted Mixed ANOVA on the weight of choice lag, with age being a between-subject variable, and phase (training vs. testing) being a within-subject variable. The results revealed a significant main effect of age, *F*(2,91) = 7.51, *p* < 0.001, $\eta_{p}^{2}$ = 0.14, and a significant main effect of phase, *F*(1,91) = 24.35, *p* < 0.001, $\eta_{p}^{2}$ = 0.21. Importantly, all age groups showed an increased weight of choice lag in the testing phase compared to the training phase, supporting our interpretation of choice lag.

**Random Exploration**

Same as in Experiment 1, we analyzed age differences in $\log(\lambda)$ using the Kruskal-Wallis test instead. The results revealed a significant difference in $\log(\lambda)$ by age, *H*(2) = 35.285, *p* < 0.001. The post-hoc analysis revealed that both child age groups ($M_{4yo}$ = 1.47, ${SD}_{4yo}$ = 0.71; $M_{5-6yo}$= 0.45, ${SD}_{5-6yo}$= 7.31) explored more randomly compared to adults ($M_{adults}$ = 4.23, ${SD}_{adults}$ = 4.41), *p*s < 0.001, but the two child age groups did not differ from each other *p* = 0.66. However, given that only one child in 5- to 6-yea-old group and 1 adult had a $\lambda$ < 1 and the majority of all age groups had a $\lambda$ > 3, we tend to not over-interpret the age differences given all participants are very deterministic.

**Additional Single-Process Model Fitting and Results**

In addition to the analyses results reported in the main text, we also examined and compared the numbers of participants best fit by the Value-based model across three age groups in Experiment 2. Our results found that 11 out of 30 4-year-olds, 22 out of 30 5- to 6-year-olds, and 31 out of 34 adults were best fit by the Value-based model in the training phase. Pairwise Fisher’s Exact tests were conducted on these proportions, and the results revealed significant differences between 4-year-olds and adults, adjusted *p* < 0.001, and between 4-year-olds and 5- to 6-year-olds, adjusted *p* = 0.03. However, 5- to 6-year-olds and adults did not differ from each other, adjusted *p* = 0.08. Similarly, 13 out of 30 4-year-olds, 17 out of 30 5- to 6-year-olds, and all of adults (34 out of 34) were best fit by the Value-based model in the testing phase. Pairwise Fisher’s Exact tests revealed significant differences between 4-year-olds and adults, and between 5- to 6-year-olds and adults, adjusted *p*s < 0.001. However, no significant differences were found between the two child groups, *p* > 0.99.

Reference

Stan Development Team (2020). RStan: The R interface to Stan*. R package version 2.21.2.* Retrieved from http://mc-stan.org/
